# *Caenorhabditis elegans* Lipin 1 moderates the lifespan‐shortening effects of dietary glucose by maintaining ω‐6 polyunsaturated fatty acids

**DOI:** 10.1111/acel.13150

**Published:** 2020-05-31

**Authors:** Yoonji Jung, Sujeong Kwon, Seokjin Ham, Dongyeop Lee, Hae‐Eun H. Park, Yasuyo Yamaoka, Dae‐Eun Jeong, Murat Artan, Ozlem Altintas, Sangsoon Park, Wooseon Hwang, Yujin Lee, Heehwa G. Son, Seon Woo A. An, Eun Ji E. Kim, Mihwa Seo, Seung‐Jae V. Lee

**Affiliations:** ^1^ Department of Biological Sciences Korea Advanced Institute of Science and Technology Daejeon South Korea; ^2^ Department of Life Sciences Pohang University of Science and Technology Pohang South Korea; ^3^ School of Interdisciplinary Bioscience and Bioengineering Pohang University of Science and Technology Pohang South Korea

**Keywords:** aging, *C. elegans*, glucose, LPIN‐1, metabolism, ω‐6 polyunsaturated fatty acids

## Abstract

Excessive glucose causes various diseases and decreases lifespan by altering metabolic processes, but underlying mechanisms remain incompletely understood. Here, we show that Lipin 1/LPIN‐1, a phosphatidic acid phosphatase and a putative transcriptional coregulator, prevents life‐shortening effects of dietary glucose on *Caenorhabditis elegans*. We found that depletion of *lpin‐1* decreased overall lipid levels, despite increasing the expression of genes that promote fat synthesis and desaturation, and downregulation of lipolysis. We then showed that knockdown of *lpin‐1* altered the composition of various fatty acids in the opposite direction of dietary glucose. In particular, the levels of two ω‐6 polyunsaturated fatty acids (PUFAs), linoleic acid and arachidonic acid, were increased by knockdown of *lpin‐1* but decreased by glucose feeding. Importantly, these ω‐6 PUFAs attenuated the short lifespan of glucose‐fed *lpin‐1*‐inhibited animals. Thus, the production of ω‐6 PUFAs is crucial for protecting animals from living very short under glucose‐rich conditions.

## Graphical abstract

LPIN‐1, phosphatidic acid phosphatase and potential transcriptional regulator, ameliorates lifespan‐shortening effects of dietary glucose by maintaining lipidostasis. Genetic inhibition of *lpin*‐*1* causes lipidostasis imbalance, including substantial reduction in the levels of ω‐6 polyunsaturated fatty acids (PUFAs), linoleic acid and arachidonic acid, in glucose‐rich conditions. This leads to substantially decreased lifespan due to increased glucose toxicity.

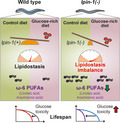

## 1 INTRODUCTION

Diets exert various effects on physiological processes, including aging and lifespan. In many organisms, including *C. elegans*, nutrient‐rich diets, such as high‐glucose‐containing ones, shorten lifespan (Alcantar‐Fernandez, Navarro, Salazar‐Martinez, Perez‐Andrade, & Miranda‐Rios, 2018; Choi, 2011; Gusarov et al., 2017; Lee et al., 2015; Lee, Murphy, & Kenyon, 2009; Schlotterer et al., 2009; Schulz et al., 2007; Seo, Kingsley, Walker, Mondoux, & Tissenbaum, 2018). Glucose‐rich diets are associated with various metabolic diseases, including diabetes, hypertension, and other age‐associated disorders in humans (Reviewed in Prinz, 2019; Stanhope, 2016). High‐glucose diets reduce lifespan by affecting lipid metabolism, and impaired metabolic flow from glucose to lipids shortens lifespan by accumulation of toxic intermediate metabolites in *C. elegans* (Lee et al., 2015). However, the mechanisms by which glucose‐rich diets shorten lifespan by regulating lipid metabolism remain unclear.

Lipin 1/LPIN‐1 is a key factor that regulates lipid metabolism in organisms ranging from *C. elegans* to mammals. Loss‐of‐function mutations in *Lipin 1* in mice cause lipodystrophy (Langner et al., 1989; Peterfy, Phan, Xu, & Reue, 2001). Mammalian Lipin 1 acts both as a phosphatidic acid phosphatase that converts phosphatidic acid to diacylglycerol (DAG) and as a transcriptional coregulator that modulates the expression of lipid consumption and lipid synthesis genes (Reviewed in Chen, Rui, Tang, & Hu, 2015; Reue & Zhang, 2008). Lipin 1 is important for the conversion of glucose to triacylglycerol (TAG), for fat storage, by acting as a phosphatidic acid phosphatase. Genetic inhibition of *lpin‐1*, which encodes the sole *C. elegans Lipin 1* homolog, reduces fat storage and can elicit the activation of sterol regulatory element‐binding protein (SREBP)/SBP‐1 (Golden, Liu, & Cohen‐Fix, 2009; Smulan et al., 2016), a key transcription factor that promotes lipid synthesis (Reviewed in Shimano & Sato, 2017). Inhibition of LPIN‐1 also disrupts the normal structure of the nuclear envelope and endoplasmic reticulum (ER) in *C. elegans* embryos during mitosis (Bahmanyar et al., 2014; Golden et al., 2009; Gorjanacz & Mattaj, 2009).

We previously conducted a genome‐wide RNAi screen using a glucose‐responsive *C. elegans* reporter, *far‐3p::gfp*, and determined the effects of *far‐3p::gfp* suppressor RNAi clones on lifespan under glucose‐rich conditions (Lee et al., 2015). We demonstrated that SREBP and MED15 protect animals against the shortening of lifespan that occurs on glucose‐rich diets by mediating the conversion of saturated fatty acids (SFAs) to unsaturated fatty acids (UFAs). Here, we characterize the functions of *far‐3p::gfp* enhancer RNAi clones, the inhibition of which increased the level of *far‐3p::gfp,* focusing on their effects on lifespan under glucose‐rich conditions. We found that *lpin‐1* was required for protecting worms from life‐shortening effects of glucose‐rich diets. We showed that the genetic inhibition of *lpin‐1* led to the upregulation of fatty acid synthesis/desaturase genes and the downregulation of lipolysis genes. These changes in gene expression appear to be caused by the activation of SBP‐1/SREBP in *lpin‐1* RNAi‐treated animals (Smulan et al., 2016). We found that the levels of several ω‐6 polyunsaturated fatty acids (PUFAs), including linoleic acid and arachidonic acid, were increased by *lpin‐1* RNAi, whereas glucose‐rich diets reversed this increase. Importantly, linoleic acid or arachidonic acid feeding ameliorated the lifespan‐shortening effects of glucose‐rich diets on *lpin‐1(RNAi)* worms. Our study indicates that the metabolic processes that convert glucose to ω‐6 PUFAs, such as linoleic acid and arachidonic acid, are crucial for protecting organisms against the lifespan‐shortening effects of excessive glucose diets in *lpin‐1*‐inhibited animals.

## 2 RESULTS

### 2.1 Lifespan screening using *far‐3p::gfp* enhancer RNAi clones identifies *lpin‐1* that protects worms from glucose toxicity

**FIGURE 1:**
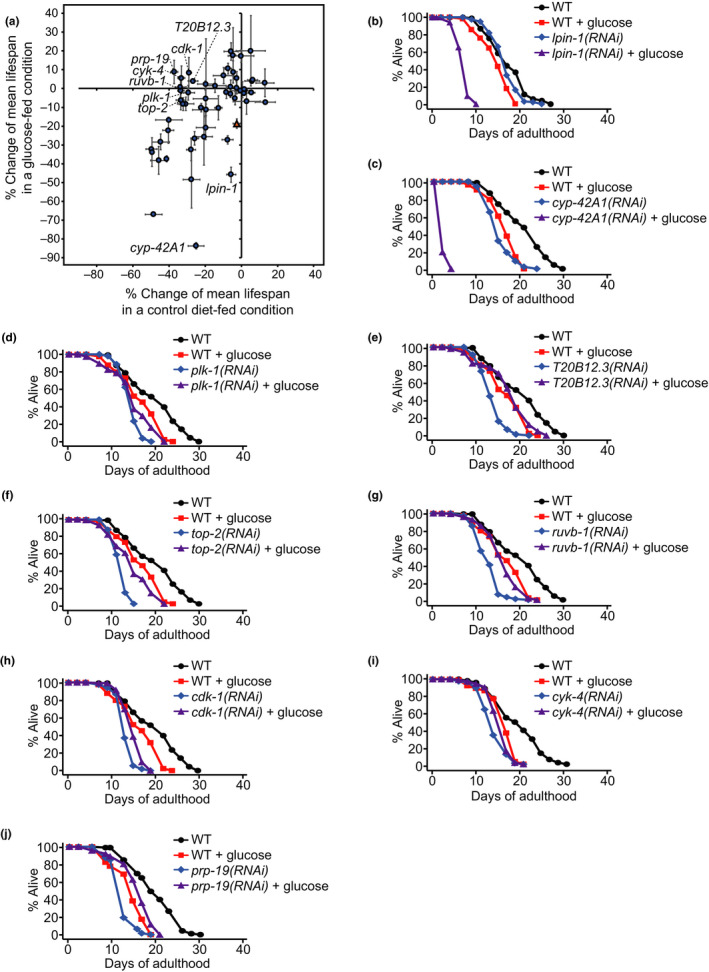
Lifespan screen using the *far‐3p::gfp* enhancer RNAi clones. (a) Percent mean lifespan changes caused by treatment with each of the 56 *far‐3p::gfp* enhancer RNAi clones (Tables S1, S2) from the previous report (Lee et al., 2015) in a control diet‐fed condition (*x‐*axis) and a glucose‐rich diet‐fed condition (*y*‐axis) compared to control RNAi. *pod‐2* RNAi (orange triangle) was used as control for glucose‐specific lifespan‐shortening effects. Error bars represent standard error of the mean (*SEM*) of changes of mean lifespan from at least two independent experiments. (b, c) RNAi targeting *lpin‐1* (b) or *cyp‐42A1* (c) further shortened lifespan on glucose‐enriched diets. A previous study reported that RNAi targeting *cyp‐42A1*, which encodes a cytochrome P450 predicted to contain heme and iron ion binding regions and oxidoreductase activity, slightly decreases the activity of eicosapentaenoic acid epoxygenase and hydroxylase (Kulas, Schmidt, Rothe, Schunck, & Menzel, 2008). (d‐j) RNAi knockdown of *plk‐1* (d), *T20B12.3* (e), *top‐2* (f), *ruvb‐1* (g), *cdk*‐*1* (h), *cyk‐4* (i), or *prp‐19* (j) decreased lifespan on control diets but had small or no effect on glucose‐rich diets. See Tables S1 and S2 for statistical analysis of the lifespan data shown in Figure 1

We chose 56 RNAi clones among the 170 *far‐3p::gfp* enhancers that we previously identified (Lee et al., 2015), for further lifespan analysis (Figure 1a, Tables S1 and S2); these candidate RNAi clones robustly increased GFP expression but did not cause severe developmental defects (Tables S1). RNAi‐mediated knockdown of *lpin‐1* or the cytochrome P450 family 42A1 (*cyp‐42A1*) gene significantly further shortened lifespan on glucose‐rich diets (Figure 1b, c). These data suggest that *lpin‐1* and *cyp‐42A1* are required for protecting worms against glucose toxicity. We also found that RNAi targeting the polo‐like kinase 1 (*plk‐1*), a homolog of human nucleolar complex‐associated 4 (*T20B12.3*), topoisomerase 2 (*top‐2*), RuvB‐like AAA ATPase 1 (*ruvb‐1*), cyclin‐dependent kinase 1 (*cdk‐1*), a homolog of Rac GTPase‐activating protein 1 (*cyk‐4*), or pre‐mRNA processing factor 19 (*prp‐19*) specifically decreased the lifespan of animals on control diets but not on glucose‐rich diets (Figure 1d‐j).

### 2.2 Inhibition of *lpin‐1* in animals on glucose‐rich diets leads to transcriptional changes in multiple biological processes, including lipid metabolism

**FIGURE 2:**
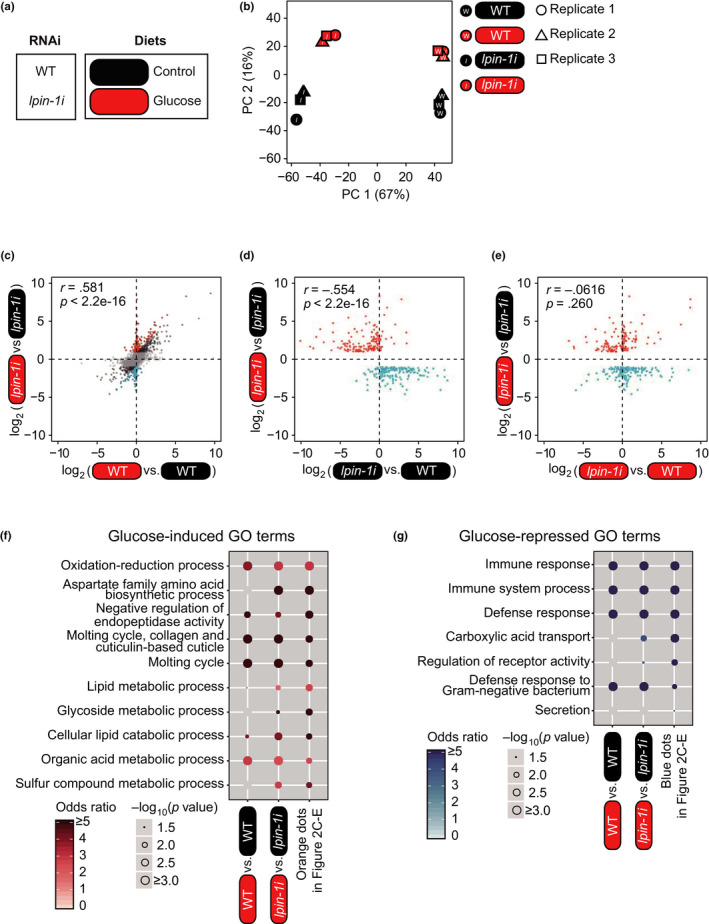
Transcriptomic changes caused by *lpin‐1* RNAi on glucose‐rich diets. (a) Labeling for four different experimental conditions used for RNA seq. WT (wild‐type) and *lpin‐1i* indicate *control(RNAi)* and *lpin‐1(RNAi)*, respectively. Round squares indicate the types of diets (black: control diets, red: glucose‐rich diets). (b) A principal component (PC) analysis showing separation among the four different conditions used for RNA seq. (c) A scatter plot showing the effects of glucose‐rich diet feeding on *lpin‐1(RNAi)* animals and WT animals. Black, orange, and blue dots indicate differentially expressed genes upon feeding glucose‐rich diets under *lpin‐1* RNAi conditions (fold change > 2, Benjamini and Hochberg (BH)‐adjusted *p* value < .05). Orange dots and blue dots indicate 161 upregulated genes and 176 downregulated genes that were changed greater (fold change > 2) in *lpin‐1* RNAi conditions than in control RNAi conditions, respectively. Pearson correlation coefficient (*r*) and its significance (*p*) are marked. We analyzed gene expression changes among specific tissues because of the possibility that mRNA abundance was affected by the smaller body size of *lpin‐1(RNAi)* than that of WT. However, we found that *lpin‐1* RNAi did not alter the impact of glucose‐rich diets on the tissue enrichment of gene expression (See Figure S2). (d, e) Scatter plots showing the effects of RNAi targeting *lpin‐1* on control diets (d) and on glucose‐rich diets (e). Pearson correlation coefficient (*r*) and its significance (*p*) are marked. (f, g) Overrepresented GO terms of genes induced (f) or repressed (g) by glucose‐rich diet feeding in a *lpin‐1*‐dependent manner. Odds ratios indicate the enrichment of genes of interest for specific terms relative to all background genes. The size of each circle is gradually increased with ‐log_10_ (*p* value), and blank indicates *p* value that is not significant (*p* > .05). *p* values were calculated by using hypergeometric test

We focused our functional analysis on LPIN‐1 because the life‐shortening effects of *lpin‐1* RNAi under glucose‐rich conditions were much greater than those under normal diet conditions (Figure 1b); in contrast, *cyp‐42A1* RNAi substantially decreased lifespan under both conditions (Figure 1c). *C. elegans* LPIN‐1 contains a phosphatidic acid phosphatase enzyme active site (DXDXT) and a transcription factor interaction motif (LXXIL) (Figure S1A) (Peterfy et al., 2001). The presence of these motifs suggests that *C. elegans* LPIN‐1 can affect gene expression directly as a transcriptional coregulator and/or indirectly as a phosphatidic acid phosphatase (reviewed in Chen et al., 2015; Reue & Zhang, 2008). We also found that glucose‐rich diets increased the nuclear localization of GFP‐fused LPIN‐1 (Figure S1B, C), raising the possibility that LPIN‐1 alters gene expression in the nucleus under glucose‐rich diet conditions. Therefore, we conducted an mRNA‐sequencing (RNA seq) analysis to determine the transcriptional changes caused by knockdown of *lpin‐1* in animals on control and glucose‐rich diets (Dataset S1); we confirmed the *lpin‐1* RNAi knockdown using qRT‐PCR and *gfp::lpin‐1* transgenic animals (Figure S1D‐F; because viable null or reduction‐of‐function *lpin‐1* mutants are currently not available, we used only RNAi for genetic inhibition of *lpin‐1* in this work, as previous studies did (Golden et al., 2009; Gorjanacz & Mattaj, 2009; Smulan et al., 2016)). A principal component analysis revealed a clear separation of transcriptomes in accordance with treatment with glucose‐rich diets and *lpin‐1* RNAi (Figure 2a, b). As dietary glucose greatly shortened the lifespan of *lpin‐1(RNAi)* animals, in contrast with that observed for wild‐type [*control(RNAi)*] worms, we first compared the genes that exhibited expression changes by glucose‐rich diets in wild‐type versus *lpin‐1(RNAi)* animals (Figure 2c; Dataset S1). We identified 350 genes that were upregulated and 327 genes that were downregulated by glucose‐rich diet feeding under *lpin‐1* RNAi conditions (Figure 2c: black, orange, and blue dots, fold change > 2, Benjamini and Hochberg (BH)‐adjusted *p* value < .05 with 17,874 genes; Dataset S1). Among them, 161 upregulated genes and 176 downregulated genes exhibited greater changes (fold change > 2) in *lpin‐1* RNAi conditions than those in control RNAi (WT) conditions (Figure 2c: orange and blue dots, respectively; Dataset S1). Interestingly, the gene expression changes caused by treatment with glucose‐rich diets in *lpin‐1(RNAi)* animals presented a negative correlation with the changes caused by *lpin‐1* RNAi in animals on control diets (Figure 2d,* r* = −0.554, *p* < 2.2e‐16; Dataset S1). In contrast, the expression of the same gene sets that were altered by treatment with glucose‐rich diets in *lpin‐1(RNAi)* animals did not correlate with the changes caused by *lpin‐1* RNAi in animals on glucose‐rich diets (Figure 2e, *r* = −0.0616, *p* = .260; Dataset S1). These results suggest that knockdown of *lpin‐1* altered the expression of a subset of genes and that this effect was partially reversed by the administration of glucose‐rich diets.

Next, we performed a gene ontology (GO) enrichment analysis of genes that exhibited expression changes after glucose‐rich diet treatment in a *lpin‐1*‐dependent manner (orange dots and blue dots in Figure 2c‐g). Among the multiple biological processes that we identified, lipid and glycoside metabolic processes were specifically enriched in the *lpin‐1*‐dependently upregulated genes under glucose‐rich conditions (orange dots in Figure 2c‐f). In contrast, carboxylic acid transport was specifically enriched in the genes downregulated under glucose‐rich conditions in a *lpin‐1*‐dependent manner (blue dots in Figure 2c‐e, g). These results suggest that LPIN‐1‐dependent responsiveness to glucose‐rich diets is associated with lipid and glycoside metabolism and carboxylic acid transport.

### 2.3 The transcriptomic changes caused by LPIN‐1 correlate positively with those by SBP‐1/SREBP and MDT‐15/MED15

**FIGURE 3:**
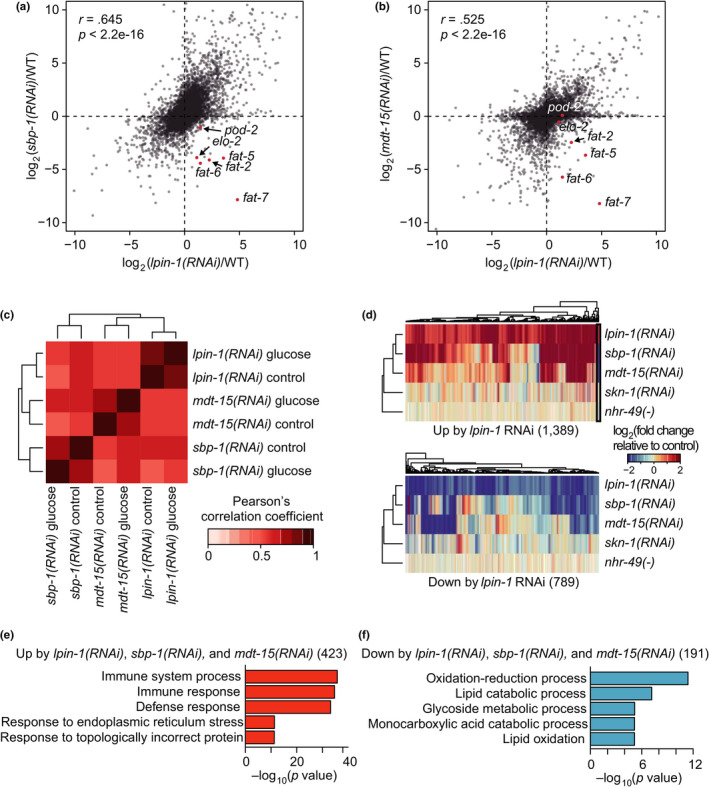
Transcriptomic changes regulated by LPIN‐1 positively correlate with those by SBP‐1 and MDT‐15. (a, b) Scatter plots showing correlation between gene expression changes caused by *lpin‐1(RNAi)* and those by *sbp‐1(RNAi)* (a) and by *mdt‐15(RNAi)* (b). Red dots indicate known SBP‐1/SREBP targets that regulate lipid metabolism. Pearson correlation coefficient (*r*) and its significance (*p*) are marked. (c) Positive correlation among gene expression changes caused by RNAi targeting *lpin‐1*, *sbp‐1,* and *mdt‐15* persisted regardless of treatment with glucose‐rich diets. (d) Heatmaps showing expression changes of genes whose expression was altered by *lpin‐1(RNAi)* compared with those by *sbp‐1(RNAi)* (Lee et al., 2015), *mdt‐15(RNAi)* (Lee et al., 2015), *skn‐1(RNAi)* (Steinbaugh et al., 2015), and *nhr‐49(nr2041)* [*nhr‐49(‐)*] mutation (Pathare et al., 2012). All these genetic perturbation conditions were compared with their relevant controls: *lpin‐1(RNAi)* versus. WT, *sbp‐1(RNAi)* versus. WT, *mdt‐15(RNAi)* versus. WT, *skn‐1(RNAi)* versus. WT, and *nhr‐49(‐)* versus. WT. Black box indicates a group of known SBP‐1/SREBP targets that regulate lipid metabolism. (e, f) Overrepresented GO terms of genes commonly up‐ (e) and down‐ (f) regulated by RNAi targeting *lpin‐1*, *sbp‐1,* and *mdt‐15*. *p* values were calculated by using hypergeometric test

We then asked whether LPIN‐1 regulated transcription by acting through SBP‐1/SREBP, a key transcription factor that regulates lipid metabolism, as *lpin‐1* RNAi elicited a compensatory activation of SREBP (Smulan et al., 2016). Consistent with the results of previous reports, *lpin‐1* RNAi upregulated several established SBP‐1/SREBP targets that mediate lipid synthesis and desaturation; these included *elo‐2*, *pod‐2*, *fat‐2*, *fat‐5*, *fat‐6*, and *fat‐7* (Figure 3a). Surprisingly, however, the overall gene expression changes caused by *lpin‐1* RNAi positively correlated with those caused by *sbp‐1* RNAi (Lee et al., 2015) (*r* = 0.645, *p* < 2.2e‐16) (Figure 3a, d). The gene expression changes caused by RNAi knockdown of *lpin‐1* also presented a positive correlation with those by RNAi knockdown of *mdt‐15* (Lee et al., 2015) (*r* = 0.525, *p* < 2.2e‐16) (Figure 3b, d), which encodes a transcriptional coregulator of SBP‐1/SREBP (Yang et al., 2006). The positive correlations observed between the gene expression changes caused by RNAi targeting *lpin‐1*, *sbp‐1*, or *mdt‐15* were preserved under glucose‐rich diet conditions (Figure 3c). In contrast, we did not observe a correlation between the gene expression changes caused by *lpin‐1* RNAi and those by genetic inhibition of SKN‐1 (Steinbaugh et al., 2015) or NHR‐49 (Pathare, Lin, Bornfeldt, Taubert, & Van Gilst, 2012) (Figure S3A, B and Figure 3d), other transcription factors that act together with MDT‐15 (Goh et al., 2014; Pang, Lynn, Lo, Paek, & Curran, 2014; Taubert, Van Gilst, Hansen, & Yamamoto, 2006). A GO enrichment analysis revealed that genes that were commonly upregulated by RNAi targeting *lpin‐1*, *sbp‐1*, or *mdt‐15* were enriched for immune and ER stress responses (Figure 3e). In contrast, genes that were commonly downregulated were enriched for oxidation–reduction, lipid catabolic, and glycoside metabolic processes (Figure 3f). These data suggest that LPIN‐1 and SBP‐1/MDT‐15 affect gene expression in the opposite direction for a small subset of lipid synthesis/desaturation genes but in the same direction for many other genes.

Next, we sought to identify SBP‐1‐independent, LPIN‐1‐specific target genes. Among 1,389 and 789 genes that were up‐ and downregulated by *lpin‐1* RNAi, respectively (fold change > 2, Benjamini and Hochberg (BH)‐adjusted *p* value < .05 with 17,874 genes), 288 upregulated and 244 downregulated genes exhibited small changes (absolute fold change < 1.5) by *sbp‐1* RNAi (Figure S3C). GO enrichment analysis revealed that genes upregulated by *lpin‐1* RNAi independently of SBP‐1 were associated with molting cycle and oxidation–reduction process (Figure S3D). Genes downregulated by *lpin‐1* RNAi independently of SBP‐1 were associated with immune response (Figure S3E). However, lipid metabolic term was not enriched in SBP‐1‐independent, LPIN‐1‐specific target genes (Figure S3D,E). Thus, consistent with a previous report (Smulan et al., 2016), *lpin‐1* RNAi appears to alter the expression of major lipid metabolic genes in an SBP‐1‐dependent manner.

### 2.4 Glucose‐rich diets and *lpin‐1* RNAi differentially alter the expression of fatty acid metabolism genes

**FIGURE 4:**
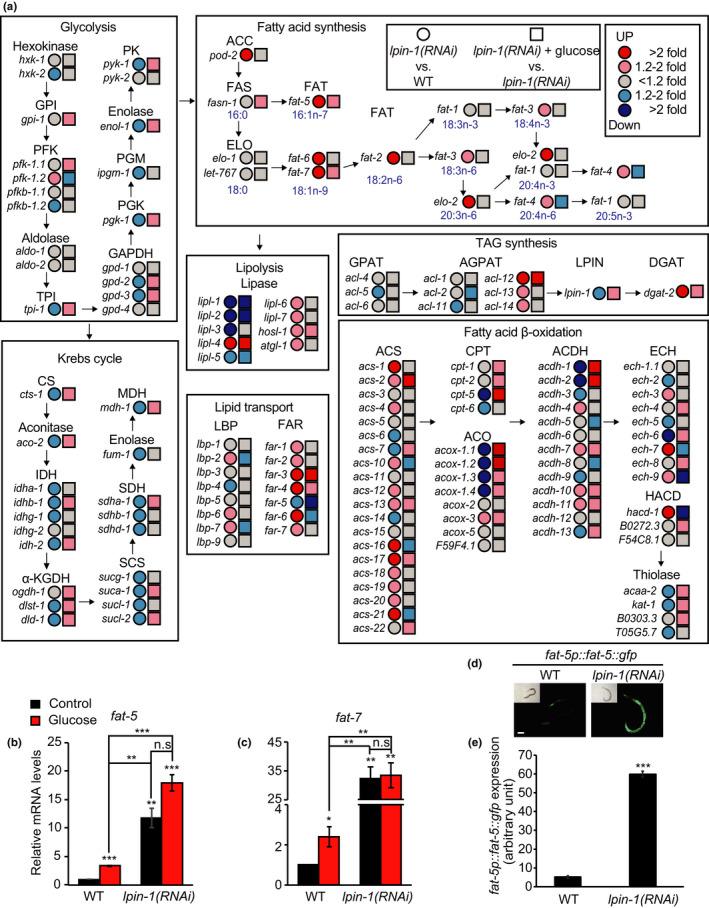
Glucose‐rich diets and *lpin‐1* RNAi differentially affect lipid metabolism‐regulating genes. (a) RNAi targeting *lpin‐1* and additional dietary glucose feeding changed the expression of genes encoding proteins crucial for glucose and lipid metabolism. The diagram was drawn similarly as described in a previous report (Lee et al., 2015). Letters in blue in the box of “Fatty acid synthesis” indicate molecular formula of fatty acids. Abbreviations are as follows: *hxk*, hexokinase; GPI (*gpi*), glucose‐6‐phosphate isomerase; PFK (*pfk*), phosphofructo‐kinase; *pfkb*, 6‐phosphofructo‐2‐kinase/fructose‐2,6‐biphosphatase; *aldo*, aldolase; TPI (*tpi*), triosephosphate isomerase; GAPDH (*gpd*), glyceraldehyde 3‐phosphate dehydrogenase; PGK, phosphoglycerate kinase; PGM (*ipgm*), phosphoglycerate mutase; *enol*, enolase; PK (*pyk*), pyruvate kinase; CS (*cts*), citrate synthase; *aco*, aconitase; IDH (*idh*), isocitrate dehydrogenase; α‐KGDH, α‐ketoglutarate dehydrogenase; *ogdh*, oxoglutarate dehydrogenase; *dlst,* dihydrolipoamide S‐succinyltransferase; *dld*, dihydrolipoamide dehydrogenase; SCS (*suc*), succinyl‐CoA synthetase; SDH (*sdh*), succinate dehydrogenase; *fum*, fumarase; MDH (*mdh*), malate dehydrogenase; ACC (*pod‐2*), acetyl‐CoA carboxylase; FAS (*fasn*), fatty acid synthase; ELO (*elo*), fatty acid elongase; *let*, lethal; FAT (*fat*), fatty acid desaturase; *lipl*, lipase‐like; *hosl*, hormone‐sensitive lipase; *atgl*, adipose triglyceride lipase; GPAT, glycerol‐3‐phosphate acyltransferases; AGPAT (*acl*), 1‐acylglycerol‐3‐phosphate O‐acyltransferase; LPIN (*lpin*), phosphatidic acid phosphatase; DGAT, diacylglycerol O‐acyltransferase; LBP, lipid‐binding protein; FAR (*far*), fatty acid and retinol‐binding protein; ACS (*acs*), acyl‐CoA synthetase; CPT (*cpt*), carnitine palmitoyltransferase; ACO (*acox*), acyl‐CoA oxidase; ACDH (*acdh*), acyl‐CoA dehydrogenase; ECH (*ech*), enoyl‐CoA hydratase; HACD (*hacd*), hydroxyacyl‐CoA dehydrogenase; *acaa*, acetyl‐CoA acyltransferase; *kat*, 3‐ketoacyl‐CoA thiolase. (b, c) *lpin‐1* RNAi increased the relative mRNA levels of *fat‐5* (b) and *fat‐7* (c) in control and glucose‐rich diet conditions (*n* = 3, two‐tailed Student's *t* test, * *p* < .05, ** *p* < .01, *** *p* < .001, n.s.: not significant). Primers targeting the coding region of *fat‐7* were used for detecting *fat‐7* mRNA. Error bars represent standard error of the mean (*SEM*). (d, e) *lpin‐1(RNAi)* increased the fluorescence intensity of FAT‐5::GFP that was expressed under a *fat‐5* promoter (*fat‐5p::fat‐5::gfp*). Representative images (d) and quantification (e) of the GFP intensity in panel d (*n* ≥ 24 from three independent experimental sets, two‐tailed Student's *t* test, *** *p* < .001). Error bars represent *SEM*. Scale bar: 100 μm. The GFP intensity values in panel d were also included in Figure S4D

We further analyzed the transcriptional changes of genes functioning in glucose and lipid metabolic pathways (Figure 4a). Consistent with our initial genome‐wide RNAi screen results using *far‐3p::gfp*, *lpin‐1* RNAi increased the mRNA level of *far‐3* (Figure 4a). Moreover, the expression of many genes that encode components in glycolysis, Krebs cycle and fatty acid β‐oxidation pathways was altered by *lpin‐1* knockdown in animals on control diets, and these changes were at least partly reversed by glucose‐rich diet feeding (Figure 4a); this result is consistent with those in Figure 2c‐d. We also noticed that *lpin‐1* knockdown increased the expression of many genes that encode lipid synthesis enzymes and lipid transporters (Figure 4a). In addition, this upregulation was further increased by glucose‐rich diets; for instance, fatty acid desaturase genes *fat‐5* and *fat‐7* were upregulated by *lpin‐1* RNAi and were further increased by glucose‐rich diets (Figure 4a). We confirmed the induction of *fat‐5* and *fat‐7* by *lpin‐1* RNAi using quantitative RT‐PCR (Figure 4b, c) and a genome‐wide RNAi screen using GFP‐fused *fat‐5* transgenic animals (*fat‐5p::fat‐5::gfp*) (Figure 4d, e, Figure S4, Tables S3 and S4). In contrast, several lipolysis‐related genes, including *lipl‐1*, *lipl‐2*, and *lipl‐5*, were downregulated by *lpin‐1* RNAi under glucose‐rich conditions (Figure 4a). Thus, inhibition of *lpin‐1* in animals on glucose‐rich diets tended to upregulate lipid synthesis/desaturation genes and to downregulate lipolysis genes, suggesting the alteration of the levels and/or the composition of lipids.

### 2.5 LPIN‐1 is required for increasing lipid levels under glucose‐rich conditions

**FIGURE 5:**
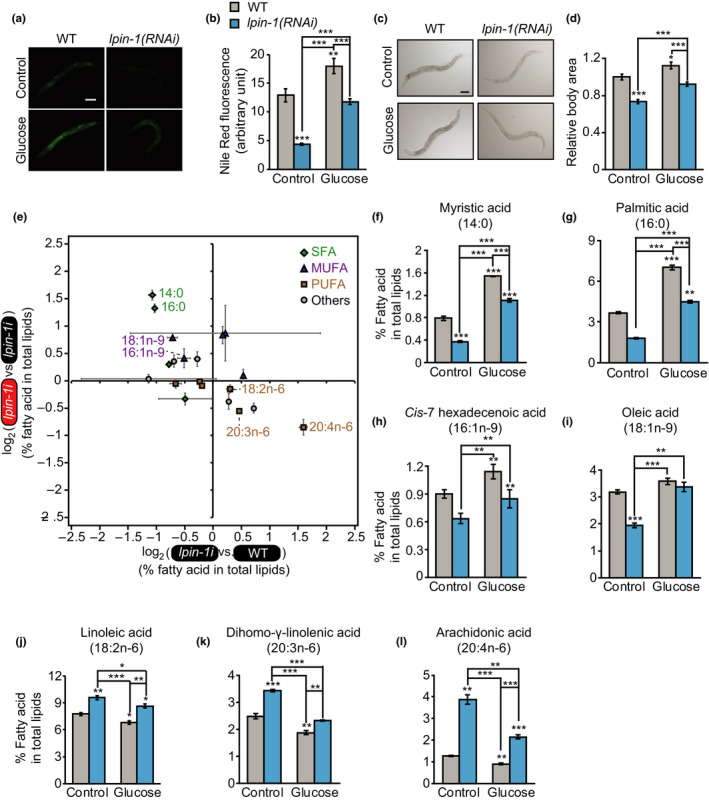
Inhibition of LPIN‐1 and glucose‐rich diet feeding affects the level and the composition of lipids in an opposite direction. (a, b) *lpin‐1* RNAi decreased lipid levels on control and glucose‐rich diets. Representative images of Nile red‐stained worms upon treating with control or *lpin‐1* RNAi with or without additional 2% glucose (a) and quantification (b) of the data shown in panel a (*n* ≥ 26 from three independent experimental sets, two‐tailed Student's *t* test, **p < .01, ***p < .001). Error bars represent standard error of the mean (*SEM*). Scale bar: 100 μm. (c, d) *lpin‐1(RNAi)* reduced the body area of worms on control and glucose‐rich diets. The relative ratio of body sizes was calculated by dividing body area of each condition to that of WT worms on control diets. Representative images (c) and the quantification (d) of the data shown in panel c (*n* ≥ 28 from three independent experimental sets). Error bars represent *SEM*. Scale bar: 100 μm. (e) The ratio of fatty acid levels in total lipids (%) (*n* = 3). X‐axis shows the ratio of percent fatty acids in *lpin‐1(RNAi)* animals to those in WT animals on control diets, indicating the effects of *lpin‐1* RNAi on changes in specific fatty acid levels. Y‐axis shows the ratio of percent fatty acids of *lpin‐1(RNAi)* worms on glucose‐rich diets versus those on control diets, indicating the effects of glucose‐rich diets on changes in specific fatty acid levels in *lpin‐1(RNAi)* worms. Green diamond, saturated fatty acids (SFAs); purple triangle, monounsaturated fatty acids (MUFAs); ocher square, polyunsaturated fatty acids (PUFAs); gray circles, others (iso‐C15 fatty acid (15:iso), iso‐C17 fatty acid (17:iso), cyclic C17 fatty acid (17:cyc), ricinoleic acid (18:1OH), and cyclic C19 fatty acid (19:cyc)). Error bars represent *SEM*. (f‐l) Percentage of specific fatty acid in total lipids. The levels of SFAs, myristic acid (14:0) (f) and palmitic acid (16:0) (g), and MUFAs, *cis*‐7 hexadecenoic acid (16:1n‐9) (h) and oleic acid (18:1n‐9) (i) were decreased by *lpin‐1* RNAi but increased by glucose‐rich diet feeding. In contrast, the levels of ω‐6 PUFAs, linoleic acid (18:2n‐6) (j) dihomo‐γ‐linolenic acid (20:3n‐6) (k) and arachidonic acid (20:4n‐6) (l), were increased by *lpin‐1* RNAi but decreased by glucose‐rich diet feeding. Two‐tailed Student's *t* test was used for statistics for this figure (*n* = 3, * *p* < .05, ** *p* < .01, *** *p* < .001). Error bars represent *SEM*. See Figure S5D‐P for the levels of the other fatty acids that we measured and Table S5 for statistical analysis

Having established that a subset of lipid synthesis/desaturation and lipolysis‐regulatory genes were respectively up‐ and downregulated by *lpin‐1* RNAi in animals on glucose‐rich diets, we tested whether these expression changes caused increases in lipid content or resulted from compensatory upregulation of SBP‐1/SREBP. Consistent with previous reports (Golden et al., 2009; Smulan et al., 2016), knockdown of *lpin‐1* decreased lipid content and lipid droplet numbers on control diets, as measured by using Nile red and Oil red O staining assays (Figure 5a, b, and Figure S5A‐C). *lpin‐1* RNAi also decreased the accumulation of lipids upon feeding with glucose‐rich diets (Figure 5a, b, and Figure S5A‐C). In addition, *lpin‐1(RNAi)* worms displayed pale and thin body phenotypes on both control and glucose‐rich diets (Figure 5c, d). Based on these data, we concluded that genetic inhibition of *lpin‐1* disrupts normal lipid accumulation in glucose‐rich diet conditions and that the decrease in lipid levels leads to a compensatory transcriptional response by SBP‐1/SREBP toward increasing lipid content.

### 2.6 Glucose‐rich diet feeding and inhibition of LPIN‐1 alter the levels of various fatty acids in an opposite direction

We then determined whether genetic inhibition of *lpin‐1* affected the levels of individual fatty acids in animals on glucose‐rich diets by performing gas chromatography and mass spectrometry (GC/MS) analyses. Interestingly, glucose‐rich diet feeding altered the levels of various fatty acids in an opposite direction to the changes caused by *lpin‐1* RNAi (Figure 5e‐l, Figure S5D‐P and Table S5). Specifically, the levels of several SFAs (myristic acid (14:0) and palmitic acid (16:0)) were decreased by *lpin‐1* RNAi but were increased by glucose‐rich diets (Figure 5f, g). In addition, the levels of several monounsaturated fatty acids (MUFAs) (*cis*‐7 hexadecenoic acid (16:1n‐9) and oleic acid (18:1n‐9)) were decreased by *lpin‐1* RNAi while being increased by glucose‐rich diet feeding (Figure 5h, i). In contrast, the levels of several ω‐6 PUFAs (linoleic acid (18:2n‐6), dihomo‐γ‐linolenic acid (20:3n‐6), and arachidonic acid (20:4n‐6)) were increased by *lpin‐1* RNAi in animals on control diets but were reduced by glucose‐rich diets (Figure 5j‐l). Overall, these data indicate that *lpin‐1* RNAi and glucose‐rich diets elicit opposite effects on the levels of various SFAs, MUFAs, and PUFAs.

### 2.7 Supplementation with ω‐6 PUFAs, linoleic acid and arachidonic acid, suppresses the shortened lifespan of *lpin‐1(RNAi)* worms on glucose‐rich diets

**FIGURE 6:**
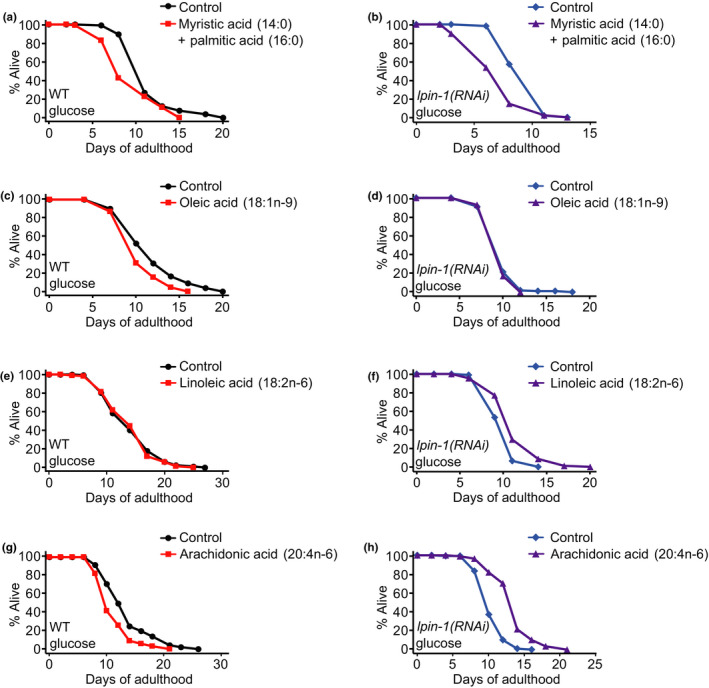
Supplementation with ω‐6 polyunsaturated fatty acids suppresses the short lifespan of glucose‐fed *lpin‐1(RNAi)* worms. (a, b) Supplementation of saturated fatty acid (SFA) mixture of myristic acid (14:0) and palmitic acid (16:0) did not prevent the life‐shortening effects of glucose‐rich diets in *control(RNAi)* (WT) (a) or *lpin‐1(RNAi)* worms (b). (c, d) Oleic acid (18:1n‐9) feeding decreased the lifespan of glucose‐fed WT (c) and did not affect that of *lpin‐1(RNAi)* worms on glucose diets (d). (e, f) Supplementation with linoleic acid (18:2n‐6) did not affect the lifespan of WT worms on glucose‐rich diets (e) (one out of two trials), while increasing the shortened lifespan of *lpin‐1(RNAi)* worms on glucose‐rich diets (f) (both trials). (g, h) Arachidonic acid (20:4n‐6) feeding did not increase the lifespan of WT worms on glucose‐rich diets (g), but lengthened the reduced lifespan of *lpin‐1(RNAi)* worms on glucose‐rich diets (h). Supplementation of each of various fatty acids that we used in this study did not appear to affect the growth of *E. coli* in general (Figure S6A). We further examined the causal role of ω‐6 PUFA metabolism in short lifespan of *lpin‐1(RNAi)* under glucose‐rich conditions by performing lifespan assays using RNAi targeting each of *fat‐1, fat‐2, fat‐4* and *fat‐6*, and overexpression of *fat‐2* in combination with *lpin‐1* RNAi (Figures S7 and S8). Among them RNAi targeting *fat‐6* decreased the lifespan of *control(RNAi)* worms on glucose‐rich diets, while not further reducing that of *lpin‐1(RNAi)* animals under glucose‐rich conditions (Figures S7A‐C and S8A‐B). Because *fat‐6* encodes a fatty acid desaturase that is crucial for the synthesis of various MUFAs and PUFAs (Figure 4a), including linoleic acid (18:2n‐6) and arachidonic acid (20:4n‐6), these results are consistent with the idea that ω‐6 PUFAs are crucial for maintaining lifespan on glucose‐rich diets. See Tables S6 and S7 for statistical analysis and additional repeats of the lifespan data shown in Figure 6 and Figure S8, respectively

We then asked whether changes in the levels of various SFAs, MUFAs, or PUFAs were responsible for the very short lifespan of *lpin‐1(RNAi)* animals on glucose‐rich diets. We measured the lifespan of wild‐type and *lpin‐1(RNAi)* worms on glucose‐rich diets upon treatment with SFAs (myristic acid (14:0) and palmitic acid (16:0)) or a MUFA (oleic acid (18:1n‐9)), the levels of which were decreased and increased by *lpin‐1* RNAi and glucose‐rich diet feeding, respectively (Figure 5e‐g, i). Treatment with SFAs (myristic acid (14:0) and palmitic acid (16:0)) further decreased the lifespan of wild‐type (Figure 6a) (Lee et al., 2015) and *lpin‐1(RNAi)* worms (Figure 6b) on glucose‐rich diets. In addition, oleic acid (18:1n‐9) feeding decreased the lifespan of wild‐type worms (Figure 6c) and did not affect the lifespan of *lpin‐1(RNAi)* worms (Figure 6d) on glucose‐rich diets. Therefore, deficiency in these SFAs or MUFAs does not appear to be responsible for the very short lifespan of *lpin‐1(RNAi)* worms on glucose‐rich diets. In contrast, the shortened lifespan of glucose‐fed *lpin‐1(RNAi)* worms was substantially restored by treatment with each of two ω‐6 PUFAs, linoleic acid (18:2n‐6) and arachidonic acid (20:4n‐6) (Figure 6f, h), whose levels were increased by *lpin‐1* RNAi while being decreased by glucose‐rich diets (Figure 5e, j, l). In contrast, the lifespan of wild‐type worms that were fed glucose‐rich diets was not increased by linoleic acid (18:2n‐6) or arachidonic acid (20:4n‐6) feeding (Figure 6e, g). Together, these data suggest that *lpin‐1* RNAi increases the levels of several ω‐6 PUFAs, including linoleic acid (18:2n‐6) and arachidonic acid (20:4n‐6), as a compensatory response to altered metabolism and that glucose‐rich diets impair this compensatory response, leading to further shortening of lifespan.

## 3 DISCUSSION

### 3.1 LPIN‐1 prevents the glucose‐induced shortening of lifespan in *C. elegans*

Although glucose is an essential nutrient, the excessive intake of glucose causes many diseases and reduces lifespan in various species (Reviewed in Lee, Son, Jung, & Lee, 2017). In the current work, we showed that LPIN‐1 protected *C. elegans* against the lifespan‐shortening effects of dietary glucose by maintaining proper lipid homeostasis. Genetic inhibition of LPIN‐1 altered the transcriptome under glucose‐rich conditions. RNAi targeting *lpin‐1* upregulated and downregulated genes encoding lipid synthesis/desaturation and lipolysis enzymes, respectively. Furthermore, the levels of several SFAs and MUFAs were reduced by *lpin‐1* RNAi, whereas they were increased by glucose‐rich diets. In contrast, the levels of several ω‐6 PUFAs, including linoleic acid (18:2n‐6) and arachidonic acid (20:4n‐6), were increased by *lpin‐1* RNAi but decreased by glucose‐rich diet feeding. We found that supplementation with linoleic acid (18:2n‐6) or arachidonic acid (20:4n‐6) substantially reversed the very short lifespan of *lpin‐1* *(* *RNAi)* worms under glucose‐rich conditions. These data suggest that the proper synthesis of ω‐6 PUFAs in worms on glucose‐rich diets is required for the maintenance of a normal lifespan. Our study highlights the importance of the orchestrated coregulation of carbohydrates and lipids for maintaining animal health under constantly changing nutrient conditions.

### 3.2 *lpin‐1* RNAi exerts opposite effects on the expression of lipid synthetic genes and lipid content

Our RNA seq analysis indicated that *lpin‐1* RNAi elicited transcriptomic changes that are predicted to increase lipid synthesis and to decrease lipolysis, which may lead to a high lipid content. This is likely in line with the role of LPIN‐1 as a transcriptional co‐activator, as the activation of mammalian Lipin 1 downregulates lipogenic enzyme‐encoding genes and upregulates fatty acid oxidation component genes by inducing PPARα (Finck et al., 2006). Contrary to this prediction, *lpin‐1*‐depleted worms display reduced lipid levels; this is likely because *lpin‐1* RNAi decreases TAG levels as a phosphatidic acid phosphatase and activates SBP‐1/SREBP, leading to increases in the expression of factors that promote lipid synthesis on normal (Smulan et al., 2016) and on glucose‐rich diets. Therefore, *lpin‐1* RNAi‐induced changes in the expression of genes that participate in lipid metabolism appear to occur as a compensatory activation of SBP‐1/SREBP under control and glucose‐rich conditions.

### 3.3 ω‐6 PUFAs protect *lpin‐1‐*deficient worms against the effects of glucose‐rich diets

In *C. elegans*, various UFAs exert beneficial effects on lifespan in a context‐dependent manner. Here, we showed that dietary ω‐6 PUFAs, linoleic acid (18:2n‐6) and arachidonic acid (20:4n‐6), the levels of which were upregulated by *lpin‐1* RNAi but downregulated by glucose feeding elicited protective effects against glucose toxicity in *lpin‐1(RNAi)* worms. Consistent with this result, the mRNA level of *fat‐4*, which encodes an enzyme that synthesizes arachidonic acid, was increased by *lpin‐1* RNAi but decreased by glucose‐rich diet feeding (Figure 4a). Previous studies have indicated that supplementation with the ω‐6 PUFAs, such as arachidonic acid (20:4n‐6) and dihomo‐γ‐linolenic acid (20:3n‐6), increases the lifespan of *C. elegans* (O'Rourke, Kuballa, Xavier, & Ruvkun, 2013). In addition, dietary MUFAs, including oleic acid (18:1n‐9), palmitoleic acid (16:1n‐7), and *cis*‐vaccenic acid (18:1n‐7), increase the lifespan of *C. elegans* at 20°C (Han et al., 2017), which is a standard laboratory culture temperature. Moreover, oleic acid (18:1n‐9) suppresses the shortening of lifespan caused by reduced UFA:SFA ratios at a low temperature (15°C) (Lee et al., 2019). However, oleic acid (18:1n‐9) did not extend the lifespan of glucose‐fed *lpin‐1(RNAi)* animals; therefore, ω‐6 PUFAs that are specifically upregulated by *lpin‐1* RNAi seem to act as key metabolites for lifespan maintenance under glucose‐rich conditions.

### 3.4 LPIN‐1 affects gene expression by acting with SREBP and MDT‐15 in the same, as well as the opposite, directions

A previous study indicated that inhibition of LPIN‐1 activates SREBP by increasing its nuclear localization (Smulan et al., 2016). Therefore, we expected that the transcriptomic changes detected in *lpin‐1(RNAi)* worms may display an overall negative correlation with those in *sbp‐1(RNAi)* worms. Surprisingly, the transcriptomic changes caused by *lpin‐1* RNAi positively correlated with those caused by the genetic inhibition of SBP‐1/SREBP, although *lpin‐1* RNAi upregulated established SBP‐1/SREBP targets that mediate lipid synthesis and desaturation. The positive correlation in transcriptomic changes between *lpin‐1* and *sbp‐1* RNAi‐treated worms is likely due to a general response to lipid disruption, or these gene sets may have similar effects on lifespan under glucose‐rich conditions. Consistent with this idea, lipid‐catabolic process term was enriched in genes commonly downregulated by *lpin‐1, sbp‐1,* and *mdt‐15* RNAi. It will be important to investigate the relationship between SBP‐1/SREBP and LPIN‐1 in the context of lifespan‐affecting roles in future research.

### 3.5 Several aspects of lipid metabolism regulated by Lipin 1 appear to be conserved between *C. elegans* and mammals

The physiological role of Lipin 1 in metabolic regulation has been relatively well established in mammals. *Lipin 1* mutant mice display defects in adipocyte function and development as well as metabolic lipodystrophy phenotypes (Chen et al., 2015; Koh et al., 2008; Langner et al., 1989; Peterfy et al., 2001). Conversely, overexpression of *Lipin 1* accelerates adipocyte differentiation and leads to obesity (Koh et al., 2008; Phan & Reue, 2005). These findings imply that mouse *Lipin 1* is crucial for maintaining fat storage and promoting lipid synthesis, similar to that observed for *C. elegans lpin‐1*. In addition, several fatty acid synthesis‐promoting genes are upregulated in *Lipin 1* knockout mice (Xu et al., 2006), and linoleic acid (18:2n‐6) and arachidonic acid (20:4n‐6) levels are increased in muscle‐specific *Lipin 1* knockout mice (Rashid et al., 2019), which is consistent with our *C. elegans* data. Therefore, similar to *C. elegans* where excessive glucose feeding accelerates age‐dependent declines in muscle function, leading to decreased healthspan and lifespan (Lee et al., 2015; Seo et al., 2018), lipotoxicity caused by *Lipin 1* knockout in mammals may be aggravated on high‐carbohydrate diets, resulting in very short lifespan.

In contrast to *Lipin 1* deficiency in mice (Langner et al., 1989; Peterfy et al., 2001), human *Lipin 1* mutations cause muscle pain and weakness without lipodystrophy (Fawcett et al., 2008; Reue & Dwyer, 2009; Zeharia et al., 2008). Nevertheless, a *Lipin 1*‐deficient person displays lower levels of plasma fatty acids during exercise than controls (Raaschou‐Pedersen et al., 2019). In addition, impaired glucose tolerance correlates with low expression levels of *Lipin 1* in people (Suviolahti et al., 2006; Yao‐Borengasser et al., 2006). These data suggest that mutations in human *Lipin 1* underlie defects in lipid and glucose metabolism. Therefore, based on these evolutionarily conserved functions of Lipin 1, it would not be surprising to find that mammalian Lipin 1 plays protective roles against the effects of glucose toxicity on lifespan and/or age‐related diseases. Moreover, it will be interesting to test whether ω‐6 PUFAs play protective roles in mammals deficient in *Lipin1*, in particular against glucose toxicity.

## 4 EXPERIMENTAL PROCEDURES

### 4.1 Strains

All strains were maintained at 20°C on nematode growth media (NGM) seeded on *E. coli* OP50 (Stiernagle, 2006). The list of strains used in this study and details are described in the Supporting Experimental Procedures.

### 4.2 Lifespan screen using *far‐3p::gfp* enhancer RNAi clones

Lifespan screen assays were performed as previously described with slight modifications (Lee et al., 2015). Detailed information is described in the Supporting Experimental Procedures.

### 4.3 Conservation of LPIN‐1 motifs in several species

Homologs of LPIN‐1 were compared to one another. Detailed information is described in the Supporting Experimental Procedures.

### 4.4 Fluorescence imaging of worms

Fluorescence imaging was performed as described previously with modifications (Lee et al., 2015). Detailed information is described in the Supporting Experimental Procedures.

### 4.5 Lifespan assays

Lifespan assays were performed as described previously with minor modifications (Lee et al., 2015). Detailed information is described in the Supporting Experimental Procedures.

### 4.6 RNA seq analysis

RNA was extracted as described previously with minor modifications (Lee et al., 2019). Detailed information is described in the Supporting Experimental Procedures.

### 4.7 A genome‐wide RNAi screen using *fat‐5p::fat‐5::gfp*

A genome‐wide RNAi screen using *fat‐5p::fat‐5::gfp* transgenic worms was performed in liquid culture systems. Detailed information is described in the Supporting Experimental Procedures.

### 4.8 Confirmation of the genome‐wide RNAi screen using *fat‐5p::fat‐5::gfp* on solid media

We confirmed the whole‐genome RNAi screen using *fat‐5p::fat‐5::gfp* on solid NGM plate. Detailed information is described in the Supporting Experimental Procedures.

### 4.9 Nile red staining

Nile red staining was performed as described previously with modification (Ashrafi et al., 2003; Pino, Webster, Carr, & Soukas, 2013). Detailed information is described in the Supporting Experimental Procedures.

### 4.10 Oil red O staining

Oil red O staining was performed as described previously with modifications (O'Rourke et al., 2013). Detailed information is described in the Supporting Experimental Procedures.

### 4.11 Body size measurement assays

*C. elegans* body sizes were measured as described previously with slight modification (Lee et al., 2019). Detailed information is described in the Supporting Experimental Procedures.

### 4.12 Quantitative RT‐PCR

Quantitative RT‐PCR was performed as described previously with minor modifications (Lee et al., 2015). Detailed information is described in the Supporting Experimental Procedures.

### 4.13 Gas chromatography and mass spectrometry (GC/MS)

GC/MS was performed as previously described with slight modifications (Lee et al., 2015). Detailed information is described in the Supporting Experimental Procedures.

### 4.14 Images of bacterial lawn on plates containing various fatty acids

Images of control or *lpin‐1* RNAi bacterial lawns on fatty acid‐containing plates were captured by using a DIMIS‐M camera (Siwon Optical Technology, Anyang, Korea). Detailed information is described in the Supporting Experimental Procedures.

## CONFLICT OF INTEREST

The authors declare no competing interests.

## AUTHOR CONTRIBUTIONS

YJ contributed to designing and performing the majority of experiments described in the manuscript, data analysis, and writing manuscript; SK contributed to survival assays, imaging, genetic screens, data analysis, and writing manuscript; SH contributed to data analysis and writing manuscript; DL contributed to performing survival assays, genetic screens, and lipid composition analysis; YY contributed to lipid composition analysis; DEJ, MA, OA, SP, WH, YL, HGS, SWAA, EJEK, HEHP, and MS participated in genetic screens; S‐JVL contributed to designing all the experiments, data analysis, and writing manuscript.

## Supporting information

Appendix S1

Dataset S1

## Data Availability

Raw data and processed data are available at Gene Expression Omnibus (https://www.ncbi.nlm.nih.gov/geo, GSE138035).
