# Supplementary material for: Caenorhabditis elegans Lipin 1 moderates the lifespan‐shortening effects of dietary glucose by maintaining ω‐6 polyunsaturated fatty acids
Source: Aging Cell. 2020 May 31;19(6):e13150. doi: 10.1111/acel.13150 (PMC7294780; doi:10.1111/acel.13150)
Supplement: Supplementary file 1 — Appendix S1 [file ACEL-19-e13150-s001.pdf]

Supporting Information

Supporting Experimental Procedures

Strains. All strains were maintained at 20°C on nematode growth media (NGM) seeded on *E. coli* strain OP50 (Stiernagle, 2006). Following strains were used in this study: N2 wild-type, IJ1618 *yhIs95[fat-5p::fat-5::gfp; odr-1p::rfp]* outcrossed 6 times with Lee laboratory N2 after integrating extrachromosomal arrays of IJ588 *yhEx144[fat-5p::fat-5::gfp; odr-1p::rfp]* with ultraviolet light (Mariol, Walter, Bellemin, & Gieseler, 2013), AG175 *unc-119(ed3) III; avEx122[lpin-1p::gfp::lpin-1::lpin-1 3'utr + unc-119(+)]*, IJ530 *yhEx121[odr-1p::RFP]*, IJ585 *yhEx141[fat-2p::fat-2::gfp; odr-1p::RFP]*. 'Lee laboratory N2' was used for lifespan assays, because wild-type N2 cultured by different laboratories displayed different lifespan (Gems & Riddle, 2000), and the 'Lee laboratory N2' was used for outcrossing mutant and transgenic strains for this study.

Lifespan screen using *far-3p::gfp* enhancer RNAi clones. Lifespan screen assays were performed as previously described with modifications (Lee et al., 2015). The number of enhancer RNAi clones was first narrowed down to 56, which elicited robust increases in GFP fluorescence (Table S1): arbitrary brightness score > 4.8 (sum of scores judged by three researchers, with the score range from -3 to +3). All the RNAi clones were verified by sequencing. dsRNA-expressing bacteria were cultured in liquid LB containing 50 μg/mL ampicillin (USB, Cleveland, OH, USA) at 37°C overnight. Cultured dsRNA-expressing bacteria were seeded on 50 μg/mL ampicillin-containing NGM with or without 2% glucose (Junsei, Tokyo, Japan; 111 mM) and cultured at 37°C overnight. The bacteria were treated with 1 mM isopropyl β-D-thiogalactoside (IPTG;

GoldBio, St. Louis, MO, USA) for induction of dsRNA overnight at room temperature. Wild-type worms were fed with dsRNA-expressing bacteria from eggs. Synchronized worms at young (day 1) adult stage were transferred onto plates with freshly seeded dsRNA-expressing bacteria. A lifespan screen was performed under 10 μM 5-fluoro-2'-deoxyuridine (FUDR; Sigma, St Louis, MO, USA)-treated conditions to prevent progeny from hatching. Deaths were determined by counting worms that did not respond to gentle touch with a sterilized platinum wire. Worms that burrowed, ruptured, or crawled off the plates were censored but included in subsequent statistical analysis. The lifespan assays for all the tested RNAi clones were performed by at least two researchers independently and semi-blindly; researchers were unaware of which gene was targeted by each of RNAi clones, except controls. Statistical analysis was performed using open-access websites, OASIS (http://sbi.postech.ac.kr/oasis) (J. S. Yang et al., 2011) and OASIS 2 (https://sbi.postech.ac.kr/oasis2/) (Han et al., 2016).

Conservation of LPIN-1 motifs in several species. Domain and motif information regarding *Caenorhabditis elegans* (worm) LPIN-1, *Homo sapiens* (human) Lipin 1, 2 and 3 and *Mus musculus* (mouse) Lipin 1, 2 and 3, *Drosophila melanogaster* (fly) Lipin, and *Saccharomyces cerevisiae* (yeast) Pah1 was obtained from open access website UniProt (https://www.uniprot.org/) (Consortium, 2019). Amino-terminal and carboxy-terminal regions of fly, yeast, and worm Lipin proteins were predicted as homologs to corresponding domains of human Lipin 1 via Basic Local Alignment Search Tool (BLAST, https://blast.ncbi.nlm.nih.gov/Blast.cgi). Nuclear localization signal (NLS) was predicted by using cNLS mapper (http://nls-mapper.iab.keio.ac.jp/cgi-bin/NLS_Mapper_form.cgi) (Kosugi,

Hasebe, Tomita, & Yanagawa, 2009). The graphical representation of sequence was illustrated by IBS 3.0 (http://ibs.biocuckoo.org/) (Liu et al., 2015). The amino-terminal and carboxy-terminal regions of worm LPIN-1, human Lipin 1, 2 and 3, mouse Lipin 1, 2 and 3, fly Lipin, and yeast Pah1 were visualized separately by employing ClustalX (v. 2.1) (iterate each alignment step) (Larkin et al., 2007).

Fluorescence imaging of worms. Fluorescence imaging was performed as described previously with modifications (Lee et al., 2015). For measuring the levels and the subcellular localization of GFP::LPIN-1, transgenic worms were fed with control or *lpin-1* RNAi bacteria, or OP50 with or without additional 2% glucose from hatching. Synchronized young (day 1) adult worms were then anesthetized with 5 mM levamisole on a 2% agarose pad on a slide glass before imaging. The images of the worms were captured by using an AxioCam HRc (Zeiss Corporation, Oberkochen, Germany) camera attached to a Zeiss Axioscope A.1 microscope (Zeiss Corporation, Oberkochen, Germany). The fluorescence intensity of worms was quantified by using ImageJ (https://imagej.nih.gov/ij/) (Schneider, Rasband, & Eliceiri, 2012). Background fluorescence signals were subtracted.

Lifespan assays. Lifespan assays were performed as described previously with slight modifications (Lee et al., 2015). After dsRNA-expressing bacteria were cultured in liquid LB containing 50 μg/mL ampicillin at 37°C overnight, the bacteria were seeded on NGM plates and incubated at 37°C overnight. One mM IPTG was added on the bacteria-seeded NGM plates for the induction of dsRNA. Briefly, worms that were grown on dsRNA-expressing bacteria-seeded

NGM plates from eggs were transferred to 10 μM FUDR-containing plates to inhibit progeny hatching with or without additional 2% glucose at young (day 1) adult stage. For double RNAi treatment, two different types of dsRNA-expressing bacteria were separately cultured using liquid LB media containing 50 μg/ml ampicillin (USB, Santa Clara, CA, USA) at 37°C overnight until the optical density (OD) value at 600 nm reached 0.9. The bacteria were then mixed to 1:1 ratio. The mixed bacteria were seeded on NGM plates and incubated at 37°C overnight. Ten mM IPTG was added on the bacteria-seeded NGM plates for the induction of dsRNA. Dead worms that did not show any movement upon gentle touch with a platinum wire were counted. The lifespan assays for each experiment were performed by at least two researchers independently for reproducibility. Worms that crawled off the plates, burrowed, or displayed ruptured vulvae or internal hatching were classified as censored worms but were included in subsequent statistical analysis. Fatty acid-treated plates were prepared as described previously with modifications (Lee et al., 2015; F. Yang et al., 2006). Specifically, 600 μM of linoleic acids (18:2n-6, Sigma, St Louis, MO, USA), arachidonic acids (20:4n-6, Sigma), or oleic acids (18:1n-9, Sigma, St Louis, MO, USA) dissolved in ethanol were mixed with NGM containing 0.1% NP-40 (Sigma, St Louis, MO, USA) and 50 μg/mL ampicillin for specific fatty acid feeding assays. For saturated fatty acid (SFA) feeding, 600 μM of myristic acid (14:0, Sigma, St Louis, MO, USA) and palmitic acid (16:0, Sigma, St Louis, MO, USA) dissolved in ethanol were mixed with NGM containing 0.1% NP-40 and 50 μg/mL ampicillin. For control plates for fatty acid feeding assays, NGM was mixed with ethanol, 0.1% NP-40 and 50 μg/mL ampicillin. Statistical analysis was performed by using OASIS2 (https://sbi.postech.ac.kr/oasis2) (Han et al., 2016), which calculates p values using log-rank (Mantel-Cox method) test.

RNA seq analysis. RNA was extracted as described previously with minor modifications (Lee et al., 2019). Worms were fed with control or *lpin-1* RNAi bacteria on control or 2% glucose-containing NGM plates from hatching. Synchronized worms were harvested when worms reached young (day 1) adult stage. RNA was extracted using RNAiso plus (Takara Bio Inc., Shiga, Japan). TruSeq (unstranded) mRNA libraries (Illumina, CA, USA) were constructed and paired-end sequencing of Illumina platform was performed by Macrogen (Seoul, South Korea). Sequencing pairs were aligned to the *C. elegans* genome WBcel235 (ce11) and Ensembl transcriptome (release 95) by using STAR (v.2.7.0e) (Dobin et al., 2013). Aligned pairs on genes or transcripts were quantified by using RSEM (v.1.3.1) (Li & Dewey, 2011). Detailed parameters of alignment and quantification referred to guidelines of ENCODE long RNA-Seq processing pipeline (https://www.encodeproject.org/pipelines/ENCPL002LPE/). Differentially expressed genes (fold change > 2 and adjusted *p* value < 0.05) were identified by using DESeq2 (v.1.22.2) (Love, Huber, & Anders, 2014). Wald test *p* values were adjusted for multiple testing using the procedure of Benjamini and Hochberg (Love et al., 2014). Gene expression changes that were greater with glucose-rich diet feeding (fold change > 2) in *lpin-1* RNAi conditions than in control RNAi conditions were defined as *lpin-1*-dependent glucose-responsive genes. GO terms enriched in certain genes were identified by GOstats (v.2.48.0) (Falcon & Gentleman, 2007) and summarized by Revigo (Supek, Bosnjak, Skunca, & Smuc, 2011). For the tissue enrichment analysis, genes of interest were compared with genes expressed in different tissues by using ‘Worm tissue’ (http://worm.princeton.edu/predictions/batch/) (Kaletsky et al., 2018). R (v.3.5.3, http://www.r-project.org) was used for plotting results. Raw data and processed data are available at Gene Expression Omnibus (https://www.ncbi.nlm.nih.gov/geo, GSE138035).

A genome-wide RNAi screen using *fat-5p::fat-5::gfp*. A genome-wide RNAi screen using a liquid culture system was performed as described previously with modifications (Lehner, Tischler, & Fraser, 2006). Each of 19,213 RNAi bacteria from commercially available *C. elegans* RNAi library (Source BioScience, Nottingham, UK) was cultured in 200 μL liquid LB media with 50 μg/mL ampicillin using 96 well plates overnight at 37°C. RNAi bacteria were treated with 4 mM IPTG to induce dsRNA at 37°C for 1 hr, and the bacteria were spun down by centrifugation (870 *g* for 10 min) and the supernatant was then discarded. Pellet was resuspended by adding liquid nematode growth media (NGM) with 4 mM IPTG and 50 μg/mL ampicillin. Worms were synchronized by bleaching gravid adult worms cultured on OP50-seeded high growth (HG) plates with bleach (mix 5 N NaOH and household bleach to 1:2 ratio) solution (Stiernagle, 2006), and the eggs were allowed to hatch to become L1 larvae in 15 mL tube with M9 buffer for synchronization at 20°C. The synchronized L1 larvae in 15 μL M9 buffer drops were counted to calculate the number of worms per volume. Approximately 20 L1 worms were then transferred into each well of RNAi bacteria-containing 96 well plates by using a micropipette. After three days of culture, day 1 adult worms in each well were scored for GFP intensity by four researchers, with a semi-quantitative range from -3 (dimmest) to +3 (brightest). Control RNAi (L4440) and *gfp* RNAi were used as control scores 0 and -3, respectively. Cut-off scores were arbitrarily set to include 1% of the total RNAi clones that were examined; RNAi clones that displayed GFP intensity scores greater and less than the arbitrary cut-off scores +2.75 and -2.50 respectively were selected as enhancer and suppressor candidate RNAi clones from the primary screen. The liquid-based screen was subsequently repeated six times using a sub-library that included the candidate RNAi clones from the primary screen.

Confirmation of the genome-wide RNAi screen using *fat-5p::fat-5::gfp* on solid media. For confirmation of the whole-genome RNAi screen using *fat-5p::fat-5::gfp* on solid NGM plates, 56 RNAi clones that substantially and reproducibly altered the intensity of GFP fluorescence from the liquid-based RNAi screen were chosen; cut-off values from 6 repeats were arbitrarily set to -1.1 and +0.7 for suppressor and enhancer RNAi clones, respectively. RNAi clones that oppositely ($\geq$ +2 for suppressors and $\leq$ -2 for enhancers) altered GFP intensity at least one of 6 sets were excluded. The 56 RNAi clones were cultured in LB media with 50 μg/mL ampicillin overnight at 37°C. Subsequently, 100 μL of RNAi bacteria was seeded and grown on solid NGM plates containing 50 μg/mL ampicillin overnight at 37°C. One mM IPTG was added on the RNAi bacteria-seeded NGM plates for the induction of dsRNA for 24 hrs at room temperature. *fat-5p::fat-5::gfp* transgenic worms at gravid adult stages were transferred onto the dsRNA-expressing bacteria seeded-plates to allow to lay eggs. The worms were anesthetized with 100 mM sodium azide and subsequently used for imaging at young (day 1) adult stage.

Nile red staining. Nile red staining was performed as described previously with modification (Ashrafi et al., 2003; Pino, Webster, Carr, & Soukas, 2013). Nile red powder (Sigma, St Louis, MO, USA) was dissolved in acetone to 0.5 mg/mL (stock solution) and stored at -20°C. Gravid adults were allowed to lay eggs overnight on control or *lpin-1* RNAi bacteria-seeded NGM plates containing 50 μg/mL ampicillin with or without 2% glucose. Synchronized worms at young adult stage (day 1) were harvested and washed once using M9 buffer with 0.01% triton X-100 (Daejung, Siheung, South Korea). Worms were fixed using 500 μL of 40% isopropanol for 3 min at room temperature. Worms were collected by spinning down briefly and the supernatant was

discarded. Fixed worms were stained using 500 μL of 3 μg/mL Nile red solution (3 μL stock solution to 500 μL of 40% isopropanol) for 2 hrs at room temperature in the dark. By adding 500 μL of M9 buffer with 0.01% Triton X-100, worms were destained for 30 min. GFP emission filter was used to detect Nile red signals. Images of worms that were placed on a 2% agarose pad on a slide glass were captured by using a camera (AxioCam HRc, Zeiss Corporation, Oberkochen, Germany) attached to a Zeiss Axioscope A.1 microscope (Zeiss Corporation, Oberkochen, Germany). For quantification, ImageJ (https://imagej.nih.gov/ij/) (Schneider, Rasband, & Eliceiri, 2012) was used. Circular region of interest (ROI) covering first two anterior intestinal cells was used for detecting Nile red fluorescence. Background signals were subtracted. For confocal imaging, images of fixed and stained worms that were placed on a 2% agarose pad on a slide glass were captured by using a confocal microscope (LSM880, Zeiss Corporation, Oberkochen, Germany). FITC emission filter was used to detect Nile red signals under the confocal microscope. Exposure time and gain of fluorescence laser were set by using Zen software (black edition v2.3; Zeiss Corporation, Oberkochen, Germany).

Oil red O staining. Oil red O staining was performed as described previously with modifications (O'Rourke, Kuballa, Xavier, & Ruvkun, 2013). Oil red O powder (Sigma, St Louis, MO, USA) was dissolved in 100% isopropanol to 0.5% (w/v) by rocking at room temperature for one day. After filtering with 0.45 μm minisart filter (Sartorius stedim, Göttingen, Germany), the 0.5% Oil red O solution was diluted to 0.3% with double distilled water (ddH2O) and placed on a shaker at room temperature for two days. During the two-day shaking, precipitated aggregates from the 0.3% Oil red O were filtered with 0.45 μm minisart filter two

more times. Prior to staining experiments, the 0.3% Oil red O was freshly prepared by filtering with 0.45 μm minisart filter once more. Worms were fed with control RNAi and *lpin-1* RNAi bacteria on control or 2% glucose-containing NGM plates with 50 μg/mL ampicillin from hatching. Worms at young (day 1) adult stage were harvested with M9 buffer in microtubes and were fixed with 60% isopropanol for two min. Worms were stained with the 0.3% Oil red O in an airtight plastic box with wet paper at 25°C over 18 hrs. After staining, worms were washed once and de-stained with M9 buffer containing 0.01% Triton X-100 (Daejung, Siheung, South Korea). The samples were stored at 4°C until microphotographs were captured. The differential interference contrast (DIC) images of worms that were placed on a 2% agarose pad on a slide glass were captured by using a camera (AxioCam HRc, Zeiss Corporation, Oberkochen, Germany) attached to a Zeiss Axioscope A.1 microscope (Zeiss Corporation, Oberkochen, Germany). The Oil red O intensity of worms was quantified by using ImageJ (https://imagej.nih.gov/ij/) (Schneider et al., 2012). The backgrounds of the images were subtracted and the images were converted to 8-bit grayscale images. By using circle tools in ImageJ (https://imagej.nih.gov/ij/) (Schneider et al., 2012), the areas that had intensities over 142 (arbitrary threshold) in first two anterior intestinal cells were measured for detecting the Oil red O signals.

Body size measurement assays. Gravid wild-type worms were allowed to lay eggs on 50 μg/mL ampicillin-containing NGM plates seeded with control or *lpin-1* RNAi bacteria under control or 2% glucose-diet conditions. Progeny were anesthetized with 5 mM levamisole at young (day 1) adult stage. Anesthetized worms were placed on a 2% agarose pad and bright field images were

captured by using a camera (AxioCam HRc, Zeiss Corporation, Oberkochen, Germany) attached to a Zeiss Axioscope A.1 microscope (Zeiss Corporation, Oberkochen, Germany). The area of worms was quantified by using ImageJ (https://imagej.nih.gov/ij/) (Schneider et al., 2012).

Quantitative RT-PCR. Quantitative RT-PCR was performed as described previously with slight modifications (Lee et al., 2015). Worms were fed with control RNAi or *lpin-1* RNAi bacteria on control or 2% glucose-containing diets for entire life. Synchronized worms were harvested at young (day 1) adult stage and washed at least twice with M9 buffer. RNA was extracted using RNAiso plus (Takara, Japan) and phase lock gel (VWR, PA, USA). For reverse transcription, random primers (6 mers, Cosmogenetech, South Korea) were used and reverse transcription was performed using ImProm-II™ Reverse Transcriptase kit (Promega, Madison, WI, USA). Quantitative real time PCR was performed by using StepOne and StepOnePlus Real-Time PCR systems (Applied Biosystems, Foster City, CA, USA) and using SYBR Green PCR Master Mix (Applied Biosystems, Foster City, CA, USA). Relative quantity of the mRNA was calculated by employing comparative Ct methods described in the manufacturer’s manual. *ama-1*, an RNA polymerase II large subunit, *tba-1*, tubulin α, and *pmp-3*, ATP binding cassette subfamily, was used as endogenous reference genes for normalization (Hoogewijs, Houthoofd, Matthijssens, Vandesompele, & Vanfleteren, 2008; Zhang, Chen, Smith, Zhang, & Pan, 2012). The levels of mRNA were normalized by multiple controls (*ama-1*, *tba-1* and *pmp-3*). Sequences of primers that were used in this study are as follows.

ama-1-F: TGGAACTCTGGAGTCACACC

ama-1-R: CATCCTCCTTCATTGAACGG

pmp-3-F: GTTCCCGTGTTC ATCACTCAT

pmp-3-R: ACACCGTCGAGAAGCTGTAGA

tba-1-F: GTACACTCCACTGATCTCTGCTGACAAG

tba-1-R: CTCTGTACAAGAGGCAAACAGCCATG

lpin-1 3’ UTR-F: GTACACGATGATGAGCTCCTAG

lpin-1 3’ UTR-R: GAATGTGATTGTTGCTGGCATC

lpin-1 5’ UTR-F: GTGACATTCGGATGTGTTAATTGG

lpin-1 5’ UTR-R: CGATTGCTCCTGAAAGTGTGG

fat-1-F: TTCACCATGCTTTCACCAACCAC

fat-1-R: GTGTACACTGGGAACCATTTAAGCC

fat-2 3’ UTR-F: CGTTACCCTCGACTATTTGA

fat-2 3’UTR-R: CACGTGTCAGGAGATTTTG

fat-4-F: GGTCTTAACTATCAGATTGAGCACC

fat-4-R: CGGAATTGCTCAATTTCAAGCC

fat-5-F: GTTCCAGAGGAAGAACTACCTCCCC

fat-5-R: GGGTGAAGCAGTAACGGAAGAGGGC

fat-6-F: CAAGAGGAGAGCAAGAAGATCC

fat-6-R: CACGGTTTGCCATTTTGCCTCG

fat-7-F: CTGCACGTCGCCGCAGCCATTG

fat-7-R: GAGAGCAAATGAGAAGACGGCC

fat-7 3’ UTR-F: CAATTGCCGATGAGTTTATCAGC

fat-7 3’ UTR -R: CTTTTATGGACAACCAACGCG

Gas chromatography and mass spectrometry (GC/MS). GC/MS was performed as previously described with slight modifications (D. Lee et al., 2015). Worms treated with *lpin-1* RNAi with or without additional 2% glucose were synchronized by using a bleach method (Stiernagle, 2006). Young (day 1) adult worms were collected using ddH_2O. Approximately 400 µL of wet worm pellet was frozen in liquid nitrogen and stored at -80°C until use. The worms were sonicated on ice (30 amplitude, 10 sec on, 2 sec off, 6 cycles) and 10 µL of total worm lysate was used for total protein quantification. Total protein amount was measured by using bicinchoninic acid (BCA) protein assay kit (Thermo Scientific, MA, USA). Three hundred µL of worm lysate was then used for the GC/MS analysis. Five mL ice-cold chloroform/methanol (1:1 ratio) was added to a glass tube. The worm lysates mixed with chloroform/methanol were vortexed and incubated for approximately 2 hrs at room temperature. The samples were then treated with 2 mL Hajra’s solution (0.2 M H_3PO_4, 1 M KCl) and mixed thoroughly by shaking. The glass tubes were centrifuged (870 *g*, 10 min) to separate lipid-containing chloroform from aqueous phase liquids. By using glass Pasteur pipettes, lipid-containing chloroform layer was separated and transferred into new glass tubes. Three mL chloroform was added to the remaining aqueous phase solution and centrifuged to further extract residual lipids. The combined lipid-containing chloroform layers were evaporated to 120 µL, and 20 µL of the solution was

subsequently used for analyzing total lipid extract.

Images of bacterial lawns on plates containing various fatty acids. Plates containing saturated fatty acid (SFA) mixture of myristic acid (14:0) and palmitic acid (16:0), oleic acid (18:1n-9), linoleic acid (18:2n-6), or arachidonic acid (20:4n-6) were prepared as described in Experimental Procedures. Control or *lpin-1* RNAi bacteria were seeded on plates containing indicated fatty acids and cultured overnight. The images of bacterial lawns were captured by using a DIMIS-M camera (Siwon Optical Technology, Anyang, Korea).

Figure S1

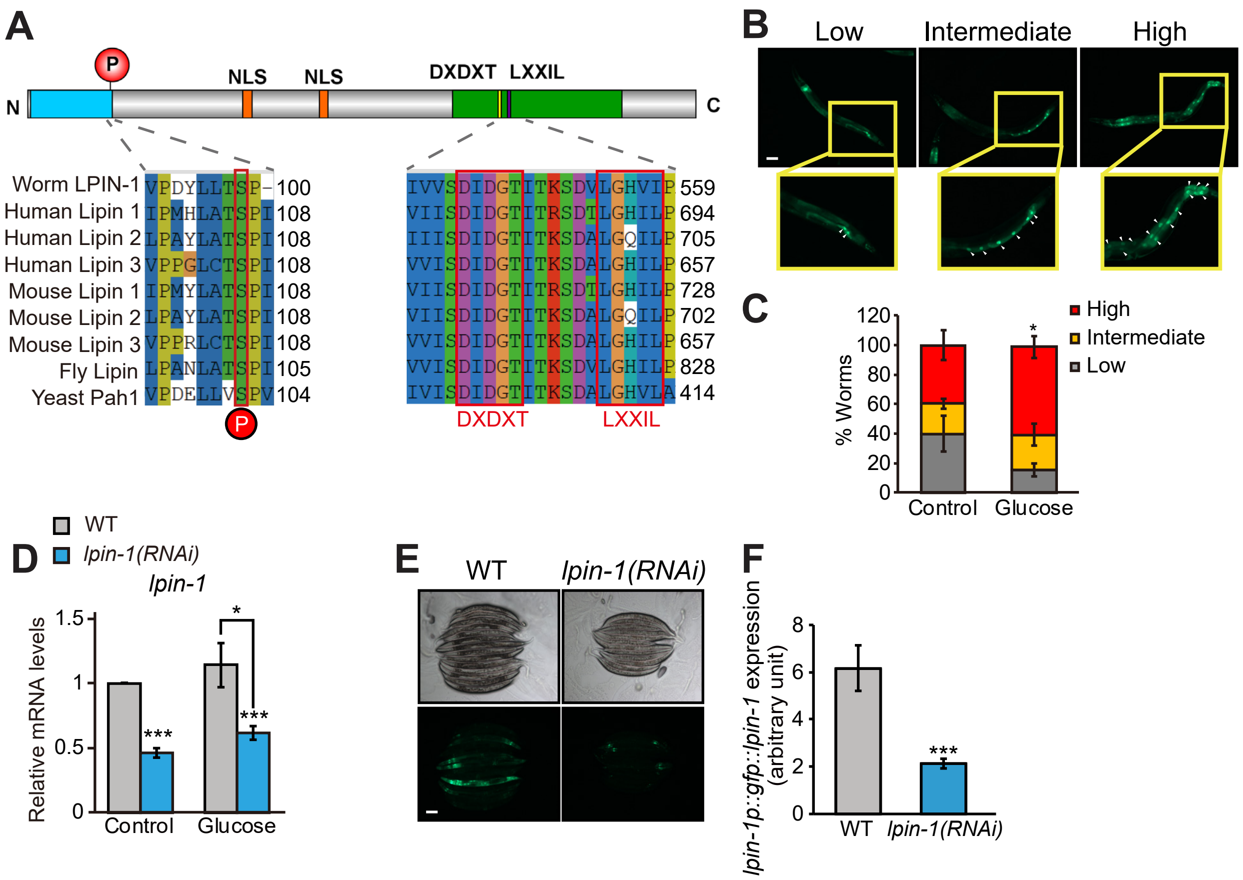
A
N
C
P
NLS
NLS
DXDXT
LXXIL
Worm LPIN-1
Human Lipin 1
Human Lipin 2
Human Lipin 3
Mouse Lipin 1
Mouse Lipin 2
Mouse Lipin 3
Fly Lipin
Yeast Pah1
DXDXT
LXXIL
B
Low
Intermediate
High
C
High
Intermediate
Low
% Worms
Control
Glucose
D
WT
lpin-1(RNAi)
lpin-1
Relative mRNA levels
Control
Glucose
E
WT
lpin-1(RNAi)
F
lpin-1p::gfp::lpin-1 expression (arbitrary unit)
WT
lpin-1(RNAi)

Supporting Figure Legends

Figure S1. Lipin 1/LPIN-1 is conserved among various species. (**A**) Conservation of amino acid sequences in Lipin 1/LPIN-1 among different species. Upper part: the domain structure of LPIN-1 that is modified from a previous paper (Reviewed in Reue & Zhang, 2008; Peterfy, Phan, Xu, & Reue, 2001). P: S99 phosphorylation site (a conserved residue with human Lipin 1 S106), NLS: nuclear localization signal, DXDXT: Asp-Xaa-Asp-Xaa-Thr catalytic motif, LXXIL: Leu-Xaa-Xaa-Ile-Leu transcriptional binding motif, blue box: a highly conserved domain near the amino-terminus of LPIN-1, green box: a highly conserved domain near the carboxy-terminus of LPIN-1. Bottom part: the comparison of worm LPIN-1, human Lipin 1, 2 and 3, mouse Lipin 1, 2 and 3, fly Lipin, and yeast Pah1. (**B, C**) Glucose feeding increased the nuclear localization of LPIN-1. Representative images of *gfp::lpin-1*-transgenic animals (**B**) and the semi-quantification of GFP::LPIN-1 nuclear localization (**C**) ($n \geq 88$ from four experimental repeats, chi-squared test, * $p < 0.05$). Arrowheads indicate nuclear localized GFP::LPIN-1 signals. Scale bar: 100 μm. Error bars represent standard error of the mean (SEM). The nuclear localization of GFP::LPIN-1 was classified into three groups (low: nuclear localization in less than 5 intestinal nuclei, intermediate: nuclear localization in more than 5 and less than 10 intestinal nuclei, high: nuclear localization in more than 10 intestinal nuclei). (**D**) Relative mRNA levels of *lpin-1* in *lpin-1(RNAi)* animals on control or glucose-rich diets. *lpin-1* RNAi significantly decreased the relative mRNA levels of *lpin-1*. Primers targeting *lpin-1* 3' UTR were used for measuring *lpin-1* mRNA levels. Error bars represent SEM ($n = 4$, two-tailed Student's *t*-test, *** $p < 0.001$, * $p < 0.05$). (**E, F**) *lpin-1* RNAi substantially decreased the level of GFP::LPIN-1. Representative images of *gfp::lpin-1* (**E**) and the quantification of GFP::LPIN-1 levels (**F**) ($n \geq 38$ from 4 experimental repeats, two-tailed Student's *t*-test, *** $p < 0.001$). Error bars represent SEM. Scale

bar: 100 μm.

Figure S2

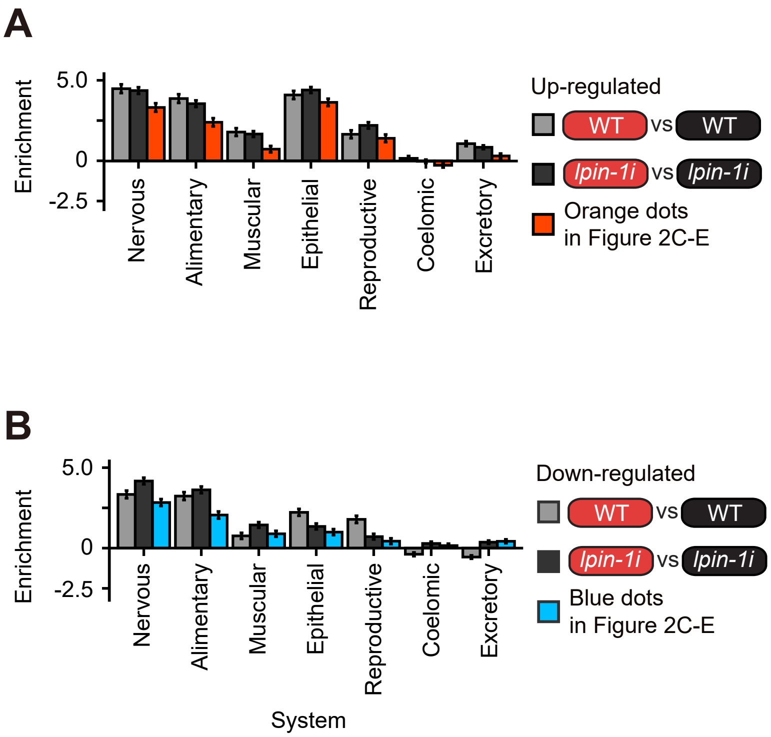
A
Enrichment
5.0
0
-2.5
Nervous
Alimentary
Muscular
Epithelial
Reproductive
Coelomic
Excretory
Up-regulated
WT vs WT
lpin-1i vs lpin-1i
Orange dots
in Figure 2C-E
B
Enrichment
5.0
0
-2.5
Nervous
Alimentary
Muscular
Epithelial
Reproductive
Coelomic
Excretory
System
Down-regulated
WT vs WT
lpin-1i vs lpin-1i
Blue dots
in Figure 2C-E

Figure S2. *lpin-1* RNAi does not alter the effects of glucose-rich diets on the tissue enrichment of gene expression. (**A, B**) Tissue-specific gene enrichment analysis among genes whose expression was up- (**A**) and down- (**B**) regulated by glucose-rich diet feeding in a *lpin-1*-dependent manner (orange dots and blue dots in Figure 2C-E, respectively) displayed similar results among various tissues. Enrichments were calculated by using hypergeometric test.

Figure S3

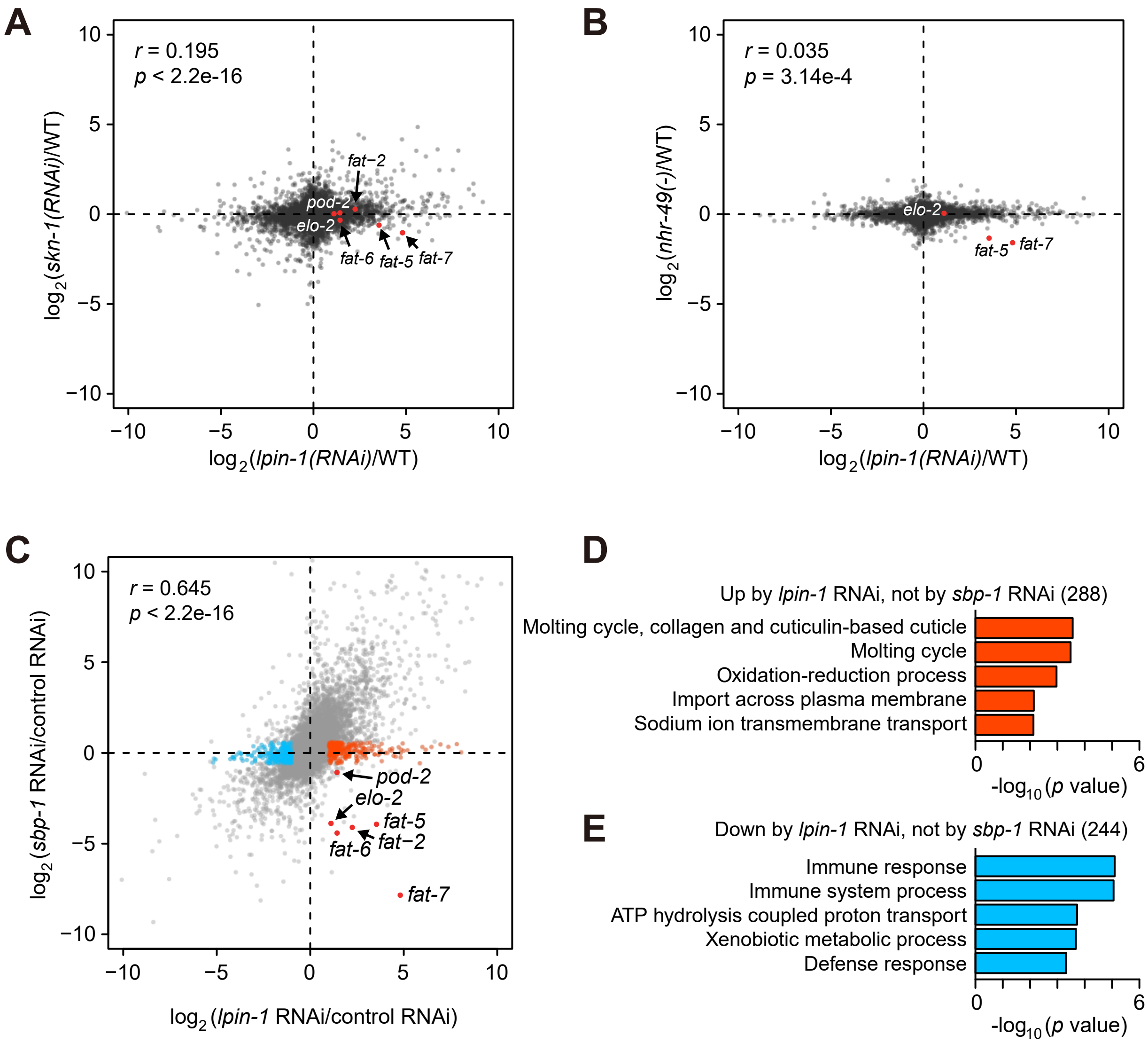

A
r = 0.195
p < 2.2e-16
fat−2
pod-2
elo-2
fat-6
fat-5
fat-7
log2(skn-1(RNAi)/WT)
log2(lpin-1(RNAi)/WT)
B
r = 0.035
p = 3.14e-4
elo-2
fat-5
fat-7
log2(nhr-49(-)/WT)
log2(lpin-1(RNAi)/WT)
C
r = 0.645
p < 2.2e-16
pod-2
elo-2
fat-5
fat−2
fat-6
fat-7
log2 (sbp-1 RNAi/control RNAi)
log2 (lpin-1 RNAi/control RNAi)
D
Up by lpin-1 RNAi, not by sbp-1 RNAi (288)
Molting cycle, collagen and cuticulin-based cuticle
Molting cycle
Oxidation-reduction process
Import across plasma membrane
Sodium ion transmembrane transport
-log10 (p value)
E
Down by lpin-1 RNAi, not by sbp-1 RNAi (244)
Immune response
Immune system process
ATP hydrolysis coupled proton transport
Xenobiotic metabolic process
Defense response
-log10 (p value)

Figure S3. Transcriptomic changes caused by *lpin-1* RNAi does not correlate with those caused by *skn-1* RNAi or *nhr-49* mutation. (**A, B**) Scatter plots showing relationship between gene expression changes caused by *lpin-1(RNAi)* and those by *skn-1(RNAi)* (**A**) (Steinbaugh et al., 2015) and *nhr-49(nr2041)* [*nhr-49(-)*] mutations (**B**) (Pathare, Lin, Bornfeldt, Taubert, & Van Gilst, 2012). Red dots indicate known SBP-1/SREBP targets that regulate lipid metabolism. Pearson correlation coefficient (*r*) and its significance (*p*) are marked. (**C**) A scatter plot showing gene expression changes caused by *lpin-1(RNAi)*, independently of *sbp-1(RNAi)* (Lee et al., 2015). Orange and blue dots indicate 288 genes up-regulated and 244 genes down-regulated by *lpin-1* RNAi (fold change > 2, Benjamini and Hochberg (BH)-adjusted *p* value < 0.05), respectively. These genes display small expression changes (absolute fold change < 1.5) in *sbp-1* RNAi-treated conditions. Pearson correlation coefficient (*r*) and its significance (*p*) are marked. (**D, E**) Overrepresented GO terms of the 288 up-regulated genes (**D**) and the 244 down-regulated genes (**E**) by *lpin-1* RNAi in an SBP-1-independent manner.

Figure S4

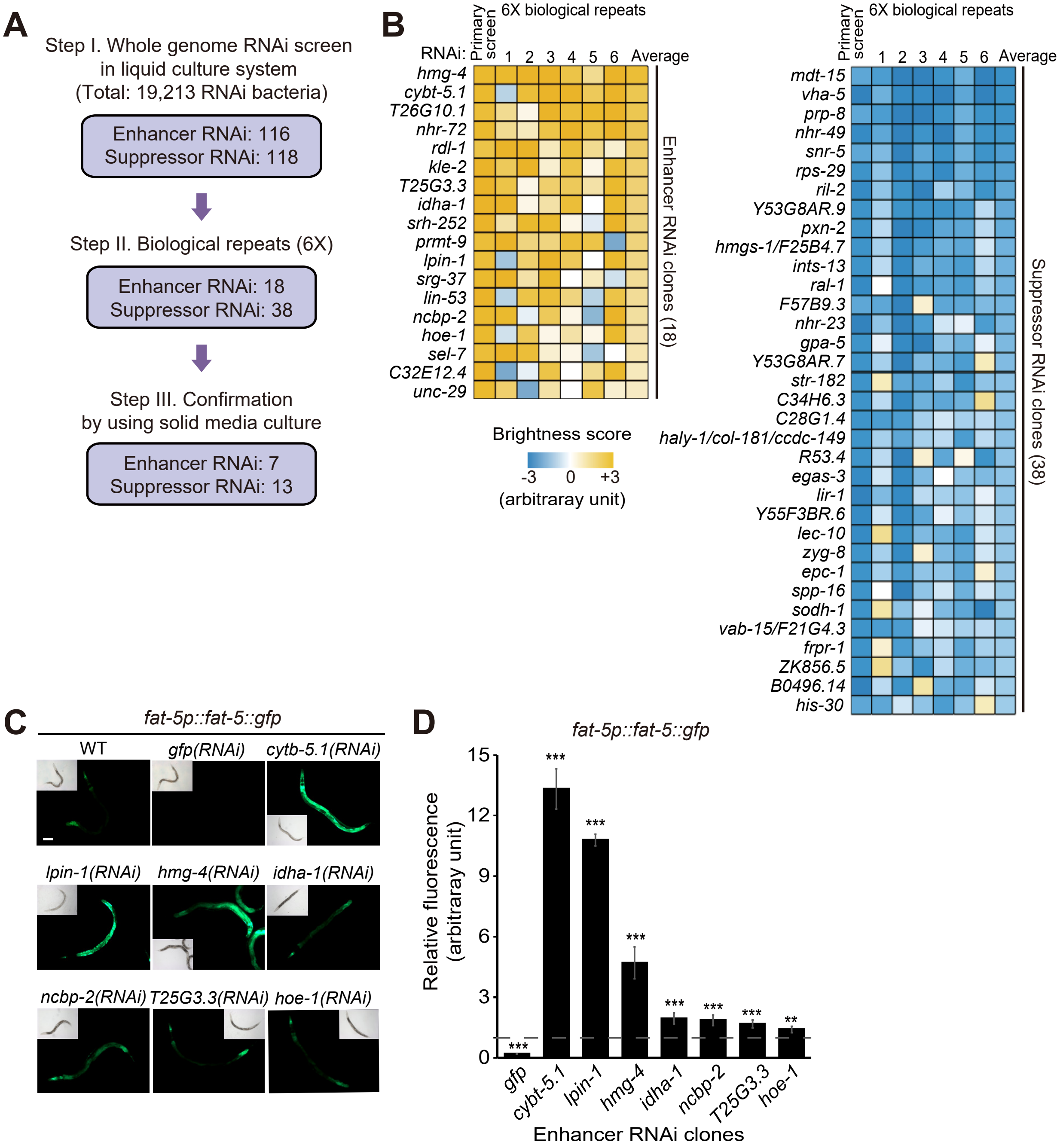
A
Step I. Whole genome RNAi screen in liquid culture system (Total: 19,213 RNAi bacteria)
Enhancer RNAi: 116
Suppressor RNAi: 118
Step II. Biological repeats (6X)
Enhancer RNAi: 18
Suppressor RNAi: 38
Step III. Confirmation by using solid media culture
Enhancer RNAi: 7
Suppressor RNAi: 13
B
RNAi:
Primary screen
6X biological repeats
1 2 3 4 5 6 Average
hmg-4
cybt-5.1
T26G10.1
nhr-72
rdl-1
kle-2
T25G3.3
idha-1
srh-252
prmt-9
lpin-1
srg-37
lin-53
ncbp-2
hoe-1
sel-7
C32E12.4
unc-29
Enhancer RNAi clones (18)
Brightness score
-3 0 +3
(arbitraray unit)
mdt-15
vha-5
prp-8
nhr-49
snr-5
rps-29
ril-2
Y53G8AR.9
pxn-2
hmgs-1/F25B4.7
ints-13
ral-1
F57B9.3
nhr-23
gpa-5
Y53G8AR.7
str-182
C34H6.3
C28G1.4
haly-1/col-181/ccdc-149
R53.4
egas-3
lir-1
Y55F3BR.6
lec-10
zyg-8
epc-1
spp-16
sodh-1
vab-15/F21G4.3
frpr-1
ZK856.5
B0496.14
his-30
Suppressor RNAi clones (38)
C
fat-5p::fat-5::gfp
WT
gfp(RNAi)
cytb-5.1(RNAi)
lpin-1(RNAi)
hmg-4(RNAi)
idha-1(RNAi)
ncbp-2(RNAi)
T25G3.3(RNAi)
hoe-1(RNAi)
D
fat-5p::fat-5::gfp
Relative fluorescence (arbitraray unit)
15
12
9
6
3
0
***
**
gfp
cybt-5.1
lpin-1
hmg-4
idha-1
ncbp-2
T25G3.3
hoe-1
Enhancer RNAi clones

Figure S4. A genome-wide RNAi screen using *fat-5p::fat-5::gfp*. (**A**) Overview of a genome-wide RNAi screen using *fat-5p::fat-5::gfp* reporter. Step I. We initially identified 116 enhancer and 118 suppressor RNAi clones that respectively increased and decreased the fluorescence levels of the *fat-5p::fat-5::gfp* in a liquid culture system. Step II. After performing the fluorescence measurement experiments 6 more times, we narrowed down the candidates to 18 enhancer and 38 suppressor RNAi clones that reproducibly altered the *fat-5p::fat-5::gfp* levels (Tables S3 and S4). Step III. Among the 18 enhancer and the 33 suppressor RNAi clones that did not show developmental defects or L1 arrest on solid media, seven and thirteen RNAi clones significantly increased and decreased the expression of *fat-5p::fat-5::gfp*, respectively (Tables S3 and S4: written in bold). Cut-off scores for each step are described in the Experimental Procedures. (**B**) Liquid-culture based RNAi experiments that identified 18 enhancer and 38 suppressor RNAi clones that reproducibly altered the *fat-5p::fat-5::gfp* levels from six repeats (Tables S3 and S4). (**C**) Representative images of *fat-5p::fat-5::gfp* worms upon treating with each of seven enhancer RNAi clones that increased the fluorescence levels in solid culture system (*control(RNAi)*: WT, *gfp(RNAi)*: control that decreased the fluorescence level). Scale bar: 100 μm. (**D**) The quantification of the GFP intensity in panel **C** ($n \geq 23$ from three independent experimental sets, two-tailed Student's *t*-test, ** $p < 0.01$, *** $p < 0.001$). Error bars represent standard error of the mean (SEM). Relative fluorescence levels were calculated by dividing the levels of fluorescence signals in specific RNAi-treated animals with those in WT worms.

Figure S5

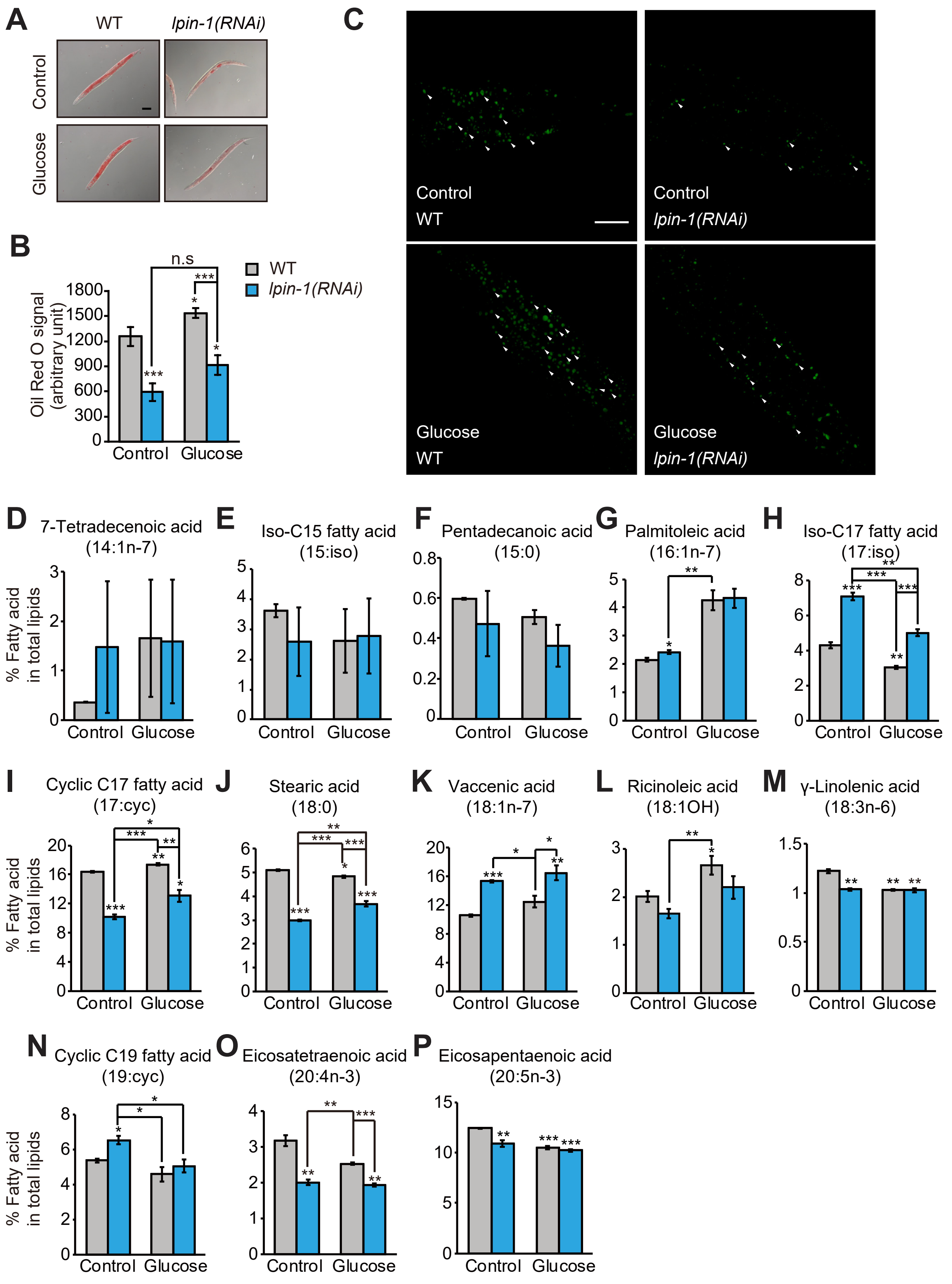
A
WT
lpin-1(RNAi)
Control
Glucose
B
Oil Red O signal (arbitrary unit)
n.s
WT
lpin-1(RNAi)
Control Glucose
C
Control WT
Control lpin-1(RNAi)
Glucose WT
Glucose lpin-1(RNAi)
D 7-Tetradecenoic acid (14:1n-7)
E Iso-C15 fatty acid (15:iso)
F Pentadecanoic acid (15:0)
G Palmitoleic acid (16:1n-7)
H Iso-C17 fatty acid (17:iso)
I Cyclic C17 fatty acid (17:cyc)
J Stearic acid (18:0)
K Vaccenic acid (18:1n-7)
L Ricinoleic acid (18:1OH)
M γ-Linolenic acid (18:3n-6)
N Cyclic C19 fatty acid (19:cyc)
O Eicosatetraenoic acid (20:4n-3)
P Eicosapentaenoic acid (20:5n-3)
% Fatty acid in total lipids

Figure S5. Changes in lipid levels or fatty acid composition caused by *lpin-1* RNAi and dietary glucose. (**A, B**) Oil red O staining of *control(RNAi)* (wild-type: WT) and *lpin-1(RNAi)* worms on control and glucose-rich diets. Representative images (**A**) and the quantification (**B**) of fat content levels in the intestine (n ≥ 26 from three independent experimental sets, two-tailed Student's *t*-test, * $p < 0.05$, *** $p < 0.001$). Error bars represent standard error of the mean (SEM). Scale bar: 100 μm. (**C**) Confocal images of the tail part of fixed Nile red-stained WT and *lpin-1(RNAi)* worms on control and glucose-rich diets. *lpin-1* RNAi decreased the number of lipid droplets in worms on control and glucose-rich diets, but did not appear to alter the distribution of lipid droplets. Arrowheads indicate the lipid droplets. Scale bar: 20 μm. (**D-P**) The levels of individual fatty acid in WT and *lpin-1(RNAi)* worms on control and glucose-rich diets. The levels of 7-tetradecenoic acid (14:1n-7) (**D**), iso-C15 fatty acid (15:iso) (**E**), pentadecanoic acid (15:0) (**F**), palmitoleic acid (16:1n-7) (**G**), iso-C17 fatty acid (17:iso) (**H**), cyclic C17 fatty acid (17:cyc) (**I**), stearic acid (18:0) (**J**), vaccenic acid (18:1n-7) (**K**), ricinoleic acid (18:1OH) (**L**), γ-linolenic acid (18:3n-6) (**M**), cyclic C19 fatty acid (19:cyc) (**N**), eicosatetraenoic acid (20:4n-3) (**O**), and eicosapentaenoic acid (20:5n-3) (**P**) (n = 3, two-tailed Student's *t*-test, * $p < 0.05$, ** $p < 0.01$, *** $p < 0.001$). Error bars represent SEM. See Figures 5F-5L for the levels of the other fatty acids that we measured and Table S5 for statistical analysis.

Figure S6

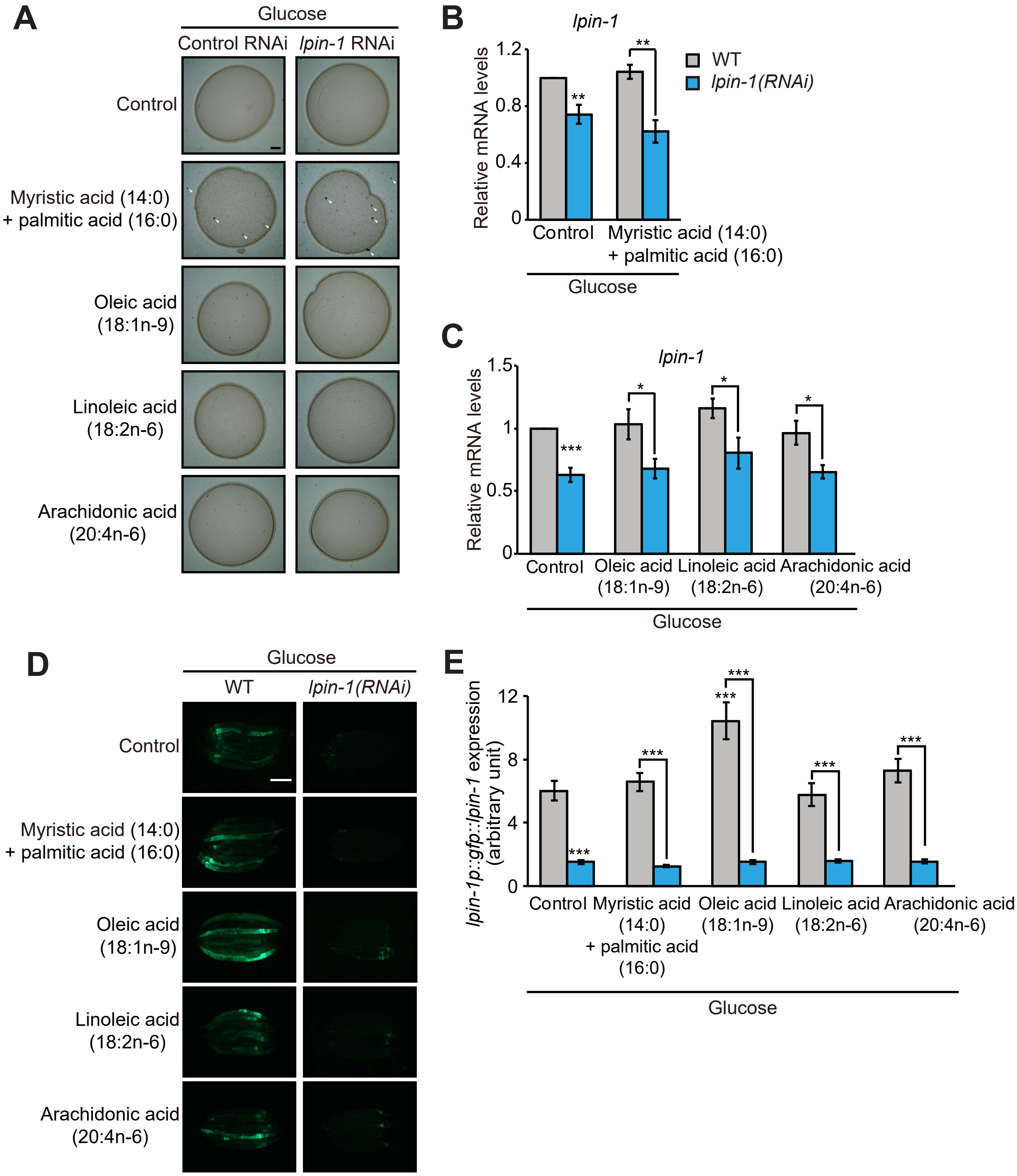
A
Glucose
Control RNAi
lpin-1 RNAi
Control
Myristic acid (14:0)
+ palmitic acid (16:0)
Oleic acid
(18:1n-9)
Linoleic acid
(18:2n-6)
Arachidonic acid
(20:4n-6)
B
lpin-1
Relative mRNA levels
1.2
0.8
0.4
0
WT
lpin-1(RNAi)
**
Control
Myristic acid (14:0)
+ palmitic acid (16:0)
Glucose
C
lpin-1
Relative mRNA levels
1.5
1
0.5
0
***
*
Control
Oleic acid
(18:1n-9)
Linoleic acid
(18:2n-6)
Arachidonic acid
(20:4n-6)
Glucose
D
Glucose
WT
lpin-1(RNAi)
Control
Myristic acid (14:0)
+ palmitic acid (16:0)
Oleic acid
(18:1n-9)
Linoleic acid
(18:2n-6)
Arachidonic acid
(20:4n-6)
E
lpin-1p::gfp::lpin-1 expression
(arbitrary unit)
12
8
4
0
***
Control
Myristic acid
(14:0)
+ palmitic acid
(16:0)
Oleic acid
(18:1n-9)
Linoleic acid
(18:2n-6)
Arachidonic acid
(20:4n-6)
Glucose

Figure S6. The images of bacteria-seeded plates and *lpin-1* RNAi efficacy in various fatty acid-treated conditions. (**A**) The images of empty vector control or *lpin-1* dsRNA-expressing bacteria-seeded plates containing indicated fatty acids. The *E. coli* lawn on a plate containing each of saturated fatty acid (SFA) mixture (myristic acid (14:0) and palmitic acid (16:0)), oleic acid (18:1n-9), linoleic acid (18:2n-6), and arachidonic acid (20:4n-6) appears to be similar to that on a control (ethanol) plate with additional 2% glucose treatments. One exception was crystal (arrowhead) formation caused by saturated fatty acid (SFA) mixture. We speculate that this may be caused by low solubility of SFAs (Khuwijitjaru, Adachi, & Matsuno, 2002). (**B, C**) *lpin-1* RNAi significantly decreased the mRNA levels of *lpin-1* in control (ethanol) and treatment with SFAs (myristic acid (14:0) and palmitic acid (16:0)) (**B**) (n = 5, two-tailed Student's *t*-test, ** $p < 0.01$), oleic acid (18:1n-9), linoleic acid (18:2n-6) and arachidonic acid (20:4n-6) conditions (**C**) (n = 7 for control, oleic acid (18:1n-9) and linoleic acid (18:2n-6)-treated conditions; n = 6 for arachidonic acid (20:4n-6)-treated conditions, two-tailed Student's *t*-test, * $p < 0.05$, *** $p < 0.001$) with additional 2% glucose treatments (gray bar: WT (*control(RNAi)*), blue bar: *lpin-1(RNAi)*). Primers targeting the 5' UTR of *lpin-1* were used for the qRT-PCR for *lpin-1* mRNA. Error bars represent standard error of the mean (SEM). (**D, E**) *lpin-1(RNAi)* reduced the fluorescence intensity of GFP::LPIN-1 under glucose-rich conditions. Representative images (**D**) and quantification (**E**) of the GFP intensity (n ≥ 55 from 4 independent experimental sets, two-tailed Student's *t*-test, *** $p < 0.001$). Error bars represent SEM. Scale bar: 200 μm.

Figure S7

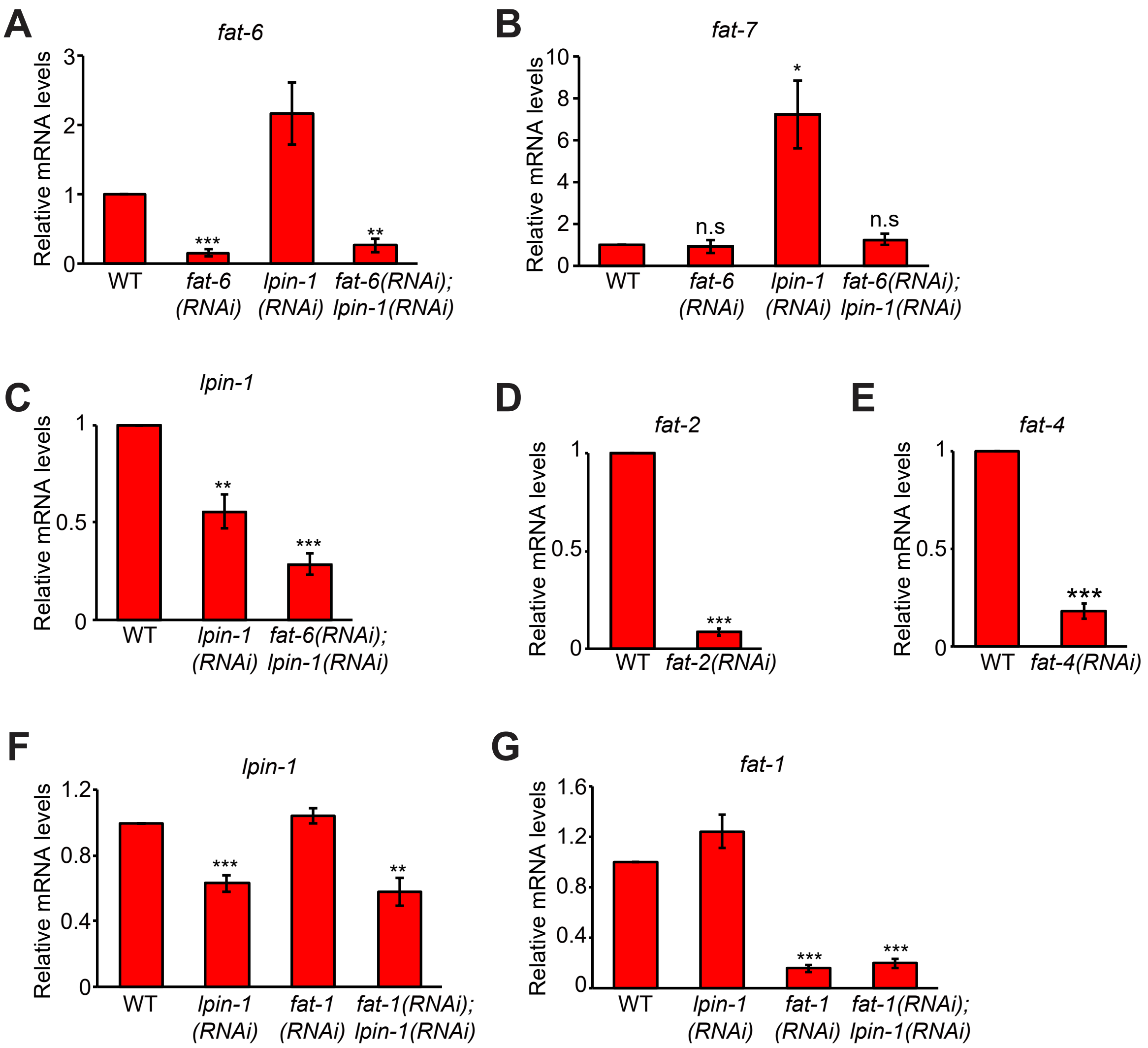
A
fat-6
Relative mRNA levels
3
2
1
0
***
**
WT
fat-6 (RNAi)
lpin-1 (RNAi)
fat-6(RNAi); lpin-1(RNAi)
B
fat-7
Relative mRNA levels
10
8
6
4
2
0
n.s
*
n.s
WT
fat-6 (RNAi)
lpin-1 (RNAi)
fat-6(RNAi); lpin-1(RNAi)
C
lpin-1
Relative mRNA levels
1
0.5
0
**
***
WT
lpin-1 (RNAi)
fat-6(RNAi); lpin-1(RNAi)
D
fat-2
Relative mRNA levels
1
0.5
0
***
WT
fat-2(RNAi)
E
fat-4
Relative mRNA levels
1
0.5
0
***
WT
fat-4(RNAi)
F
lpin-1
Relative mRNA levels
1.2
0.8
0.4
0
***
**
WT
lpin-1 (RNAi)
fat-1 (RNAi)
fat-1(RNAi); lpin-1(RNAi)
G
fat-1
Relative mRNA levels
1.6
1.2
0.8
0.4
0
***
***
WT
lpin-1 (RNAi)
fat-1 (RNAi)
fat-1(RNAi); lpin-1(RNAi)

Figure S7. Measurements of the efficiency of RNAi targeting *fat-1, fat-2, fat-4* or *fat-6* with or without *lpin-1* RNAi. (**A, B**) Relative mRNA levels of *fat-6* and *fat-7* in WT (*control(RNAi)*) on glucose-rich diets. RNAi targeting *fat-6* significantly decreased the relative mRNA levels of *fat-6* (**A**), but did not significantly affect those of *fat-7* (**B**) (n = 3, two-tailed Student's *t*-test, *** $p < 0.001$, * $p < 0.05$, n.s.: not significant). Although RNAi targeting *fat-6* is expected to target *fat-7* due to strong homology, the marginal effect of *fat-6* RNAi on *fat-7* mRNA levels is likely caused by the compensatory up-regulation of *fat-7* expression in response to genetic inhibition of *fat-6* (Brock, Browse, & Watts, 2006). Primers targeting the 3' UTR region of *fat-7* were used for *fat-7* mRNA qRT-PCR. (**C**) *lpin-1* RNAi efficacy in WT or *fat-6(RNAi)* animals. *lpin-1* RNAi significantly decreased the relative mRNA levels of *lpin-1* on glucose-rich diets. Primers targeting the 5' UTR of *lpin-1* were used for *lpin-1* mRNA qRT-PCR (n = 3, two-tailed Student's *t*-test, *** $p < 0.001$, ** $p < 0.01$). (**D, E**) *fat-2* RNAi and *fat-4* RNAi significantly decreased the mRNA levels of *fat-2* (**D**) and *fat-4* (**E**), respectively, on glucose-rich diets. (n = 3, two-tailed Student's *t*-test, *** $p < 0.001$). (**F, G**) Relative mRNA levels of *lpin-1* (**F**) and *fat-1* (**G**) in WT and *lpin-1(RNAi)* animals on glucose-rich diets. Treatment with *lpin-1* RNAi and *fat-1* RNAi significantly decreased the mRNA levels of *lpin-1* and *fat-1,* respectively (n = 4, two-tailed Student's *t*-test, *** $p < 0.001$, ** $p < 0.01$). Error bars represent SEM.

Figure S8

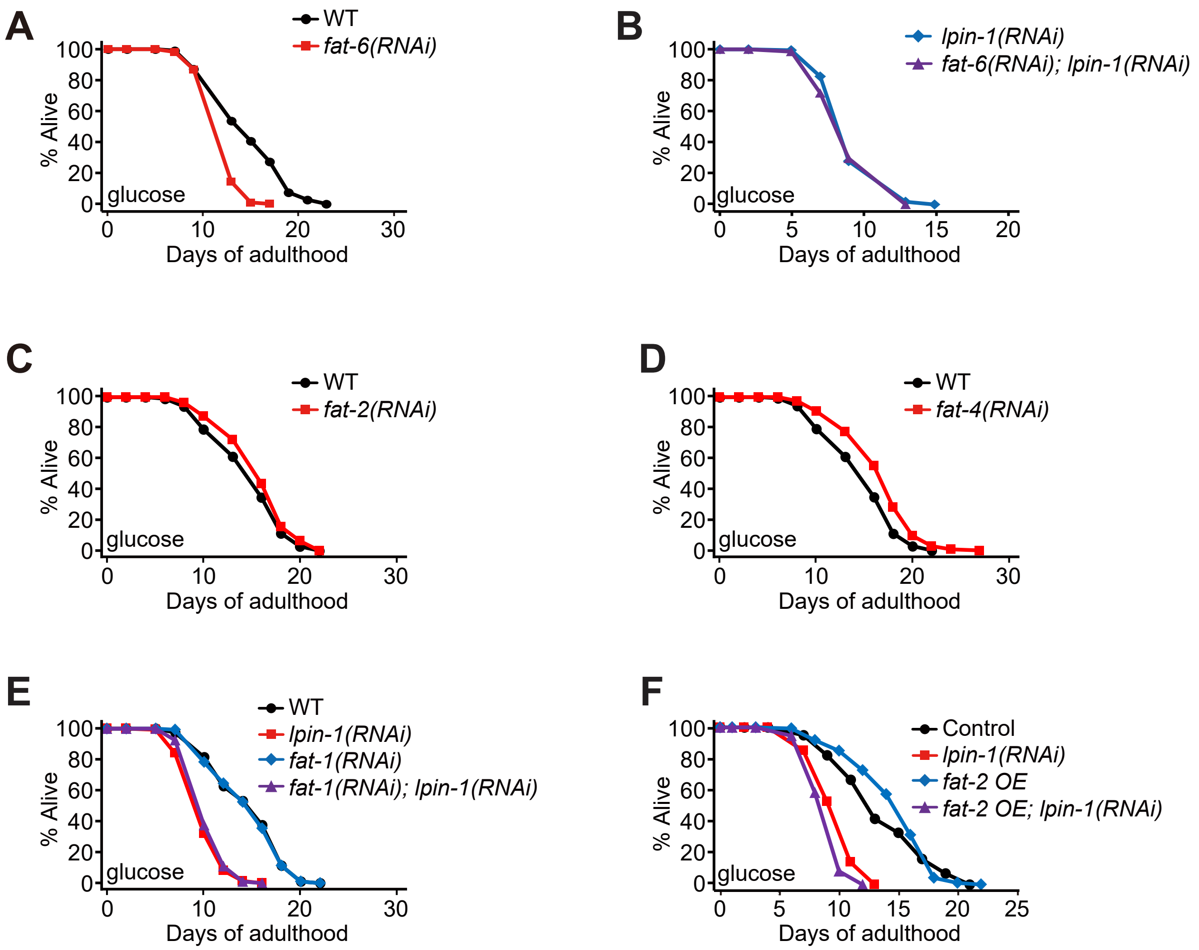
A
WT
fat-6(RNAi)
% Alive
glucose
Days of adulthood
B
lpin-1(RNAi)
fat-6(RNAi); lpin-1(RNAi)
% Alive
glucose
Days of adulthood
C
WT
fat-2(RNAi)
% Alive
glucose
Days of adulthood
D
WT
fat-4(RNAi)
% Alive
glucose
Days of adulthood
E
WT
lpin-1(RNAi)
fat-1(RNAi)
fat-1(RNAi); lpin-1(RNAi)
% Alive
glucose
Days of adulthood
F
Control
lpin-1(RNAi)
fat-2 OE
fat-2 OE; lpin-1(RNAi)
% Alive
glucose
Days of adulthood

Figure S8. The effects of RNAi targeting *fat-1*, *fat-2*, *fat-4* or *fat-6*, and *fat-2* overexpression with *lpin-1* RNAi on lifespan under glucose-rich conditions. (**A**) RNAi targeting *fat-6* decreased the lifespan of glucose-fed worms. (**B**) *fat-6* RNAi did not further decrease the short lifespan of *lpin-1(RNAi)* in glucose-rich diet conditions. (**C, D**) RNAi targeting *fat-2,* which encodes a fatty acid desaturase that catalyzes the synthesis of linoleic acid (18:2n-6; Figure 4A), or *fat-4,* which encodes a fatty acid desaturase that catalyzes the synthesis of arachidonic acid (20:4n-6; Figure 4A), did not shorten lifespan on glucose-enriched diets. (**E**) Knockdown of *fat-1,* which encodes an enzyme required for synthesizing metabolites that are located upstream of ω-6 PUFAs in fatty acid synthesis pathway (Figure 4A), did not change the lifespan of WT or *lpin-1(RNAi)* animals on glucose-rich diets. (**F**) Overexpression of *fat-2* did not restore the short lifespan of control (*odr-1p::rfp*) or *lpin-1(RNAi)* animals under glucose-rich conditions. See Table S7 for statistical analysis and additional repeats of the lifespan data shown in Figure S8.

Supporting Tables

Table S1. Summary of lifespan screen using *far-3p::gfp* enhancer RNAi clones.

	Gene ID	Gene name	Description	Average score from a genome-wide RNAi screen (Lee et al., 2015)	Lifespan Changes (%)		
					Control diets	Glucose diets	glucose specificity
1	*Y80D3A.5*	*cyp-42A1**	Cytochrome P450 family	5.4	-25.0%	-83.7%	-58.7%
2	*H37A05.1*	*lpin-1*	Phosphatidate phosphatase Lipin 1, Lipin 2, and Lipin 3	5.0	-5.6%	-45.8%	-40.2%
3	*F56B3.2*	*F56B3.2*	Unknown	8.9	-27.6%	-48.4%	-20.8%
4	*K06B4.4*	*K06B4.4*	Unknown	5.3	13.2%	-7.2%	-20.5%
5	*ZK112.2*	*ncl-1*	Alpha of tripartite motif-containing protein 3	6.3	-7.6%	-27.3%	-19.7%
6	*C12C8.3*	*lin-41*	Tripartite motif-containing protein 71	8.3	-48.6%	-66.9%	-18.4%
7	*W04G3.1*	*lpr-6*	Lipocalin-related protein	4.8	13.0%	3.0%	-10.0%
8	*C50B6.3*	*C50B6.3*	Unknown	5.3	2.9%	-6.8%	-9.7%
9	*C01C10.1*	*clc-2*	Claudin-like in Caenorhabditis	6.4	5.6%	-2.2%	-7.7%
10	*F53B7.3*	*F53B7.3*	Pre-mRNA-splicing factor ISY1 homolog	5.8	-20.7%	-25.7%	-5.0%

11	*M03F4.6*	*M03F4.6*	Unknown	6.8	-27.8%	-32.5%	-4.7%
12	*C18D11.3*	*C18D11.3*	Unknown	4.8	1.5%	-2.5%	-4.0%
13	*Y76A2B.3*	*acs-5*	Fatty acid CoA synthetase	8.5	2.0%	-0.9%	-2.8%
14	*F31E3.1*	*ceh-20*	Pre-B-cell leukemia transcription factor 2	4.8	5.9%	4.0%	-2.0%
15	*T01C3.8*	*mut-15*	Mutator	4.8	1.6%	-0.3%	-1.9%
16	*Y53F4B.14*	*Y53F4B.14*	Human PET100 ortholog (PET100 cytochrome c oxidase chaperone)	5.0	-3.5%	-5.1%	-1.6%
17	*R10D12.7*	*R10D12.7*	Unknown	5.0	6.2%	4.6%	-1.6%
18	*Y38C1BA.2*	*snn-1*	Synapsin-2 isoform IIa	4.8	0.5%	-0.6%	-1.2%
19	*F32D1.10*	*mcm-7*	DNA replication licensing factor MCM7	7.5	-19.7%	-20.9%	-1.2%
20	*T01G9.6*	*kin-10*	Casein kinase II subunit beta	7.4	-25.8%	-26.5%	-0.7%
21	*F48C1.1*	*aman-3*	Alpha-mannosidase 2	5.0	-0.7%	-1.1%	-0.5%
22	*R74.3*	*xbp-1*	X-box-binding protein 1	4.8	-12.7%	-10.3%	2.4%
23	*C01G10.1*	*C01G10.1*	Unknown	5.0	-2.7%	0.4%	3.1%
24	*C47E12.4*	*pyp-1*	Inorganic pyrophosphatase	7.5	-6.0%	-2.8%	3.2%
25	*T01E8.3*	*plc-3*	Phospholipase C gamma	6.4	-41.3%	-37.4%	3.9%
26	*T23B5.1*	*prmt-3*	Protein arginine N-methyltransferase 10	5.8	-8.4%	-1.9%	6.4%
27	*C04B4.3*	*lips-2*	Lipase-related protein	4.8	-5.9%	0.9%	6.8%

28	*F37E3.1*	*ncbp-1*	Nuclear cap-binding protein subunit 1	6.0	-45.7%	-38.2%	7.5%
29	*C29H12.3*	*rgs-3*	Regulator of G-protein signaling 8	6.0	-2.6%	5.4%	8.0%
30	*R11A8.7*	*R11A8.7*	Ankyrin repeat and KH domain-containing protein 1	6.5	-19.6%	-11.3%	8.3%
31	*T27B1.2*	*ztf-19*	Zinc finger protein 544	5.5	-22.2%	-10.1%	12.1%
32	*T09E8.2*	*him-17*	High incidence of males	4.9	-4.5%	8.8%	13.3%
33	*T22D1.10*	*ruvb-2*	RuvB-like 2	5.6	-19.7%	-5.4%	14.3%
34	*ZK858.4*	*mel-26*	Speckle-type POZ protein	7.4	5.2%	20.0%	14.9%
35	*T12F5.4*	*lin-59*	Probable histone-lysine N-methyltransferase ASH1L	5.0	-49.4%	-33.8%	15.6%
36	*T14B4.7*	*dpy-10*	Cuticle collagen protein	6.8	-14.5%	1.5%	16.0%
37	*Y110A7A.11*	*use-1*	Vesicle transport protein USE1	5.6	-44.7%	-28.4%	16.3%
38	*C54G10.3*	*pmp-3*	ABC transporter	5.0	-9.9%	6.8%	16.7%
39	*B0395.2*	*mboa-1*	Acyl-CoA:cholesterol ('sterol') O-acyltransferase	6.6	-0.2%	17.3%	17.5%
40	*H13N06.3*	*gob-1*	Trehalose-6-phosphatase	7.3	-49.8%	-32.2%	17.5%
41	*F44B9.3*	*cit-1.2*	Cyclin-K	5.1	-7.3%	10.6%	18.0%
42	*Y47G6A.20*	*rnp-6*	Poly(U)-binding-splicing factor PUF60	5.6	-40.5%	-22.3%	18.2%
43	*T19B10.2*	*T19B10.2*	Unknown	6.1	-19.6%	2.4%	22.0%

44	*F01F1.12*	*aldo-2*	Fructose-bisphosphate aldolase	6.3	-4.7%	17.6%	22.3%
45	*F30H5.1*	*unc-45*	Protein that contains an N-terminal TRP (tetratricopeptide repeat)	6.4	-30.8%	-8.2%	22.6%
46	*F29G9.4*	*fos-1*	Proto-oncogene c-Fos	6.1	-40.0%	-16.7%	23.3%
47	*K08A8.2*	*sox-2*	Transcription factor SOX-2	5.8	-32.2%	-8.3%	24.0%
48	*F07A11.6*	*din-1*	Msx2-interacting protein	4.8	-5.7%	19.7%	25.5%
49	*C14B9.4*	*plk-1*	Serine/threonine-protein kinase PLK2	6.3	-29.4%	-2.2%	27.2%
50	*K12D12.1*	*top-2*	DNA topoisomerase 2	5.8	-33.4%	-6.2%	27.2%
51	*T20B12.3*	*T20B12.3*	Nucleolar complex protein 4 homolog	6.3	-26.7%	4.0%	30.7%
52	*C27H6.2*	*ruvb-1*	RuvB-like 1	5.2	-33.5%	-1.2%	32.2%
53	*F26A10.2*	*F26A10.2*	MDS1 and EVI1 complex locus protein EVI1	5.3	-33.6%	0.9%	34.5%
54	*T05G5.3*	*cdk-1*	Cyclin-dependent kinase	4.9	-29.0%	8.4%	37.4%
55	*K08E3.6*	*cyk-4*	Rac GTPase-activating protein 1	6.7	-33.2%	5.6%	38.7%
56	*T10F2.4*	*prp-19*	Pre-mRNA-processing factor 19	6.3	-37.2%	8.8%	46.0%

Average scores from a genome-wide RNAi screen were calculated by dividing total scores made by three independent researchers with the number of counts (Lee et al., 2015). Among 170 enhancer RNAi clones, we selected 56 RNAi clones that had average scores over 4.8 for the lifespan screen.

Lifespan changes (%) indicate average lifespan changes calculated by comparing the lifespan of each RNAi-treated animals with that of

control RNAi-treated animals on control diets or on glucose-rich diets.

Lifespan assays were performed by at least two independent researchers.

Glucose specificity (%) was calculated by subtracting lifespan changes (%) on control diets from lifespan changes (%) on glucose diets.

The gene list was sorted by ascending order of glucose specificity (%).

**cyp-42A1* was not initially included in enhancer RNAi clones (Lee et al., 2015) but instead *Y80D3A.6* RNAi was included. Because *Y80D3A.6* is predicted as a dead gene (based on WormBase (https://wormbase.org/)), we tested which gene was targeted by *Y80D3A.6* RNAi by using BLAST and subsequently found that a portion of *cyp-42A1* was targeted. Therefore, we substituted *Y80D3A.6* with *cyp-42A1*.

Table S2. Lifespan screen data of *far-3p::gfp* enhancer RNAi-treated animals.

Trial	Strain/treatment	Mean lifespan ±SEM (days)	75th percentile	% change[Δ]	Number of animals that died/total	*p* value vs. control	Figures in text
#1	N2/Control RNAi	20.6±0.6	25		92/120		
	N2/Control RNAi glucose	13.2±0.4	17	-35.9%	114/120	*p*<0.0001	
	N2/*pod-2* RNAi*	18.7±0.5	23	-9.6%	82/120	0.0016	
	N2/*pod-2* RNAi glucose*	9.7±0.1	13	-53.0%<br>-26.7% (ctrl RNAi glc)	114/120	*p*<0.0001<br>*p*<0.0001(ctrl RNAi glc)	
	N2/*F56B3.2A* RNAi	14.7±0.4	17	-28.7%	114/120	*p*<0.0001	
	N2/*F56B3.2A* RNAi glucose	7.8±0.3	11	-62.1%<br>-40.9%(ctrl RNAi glc)	92/120	*p*<0.0001<br>*p*<0.0001(ctrl RNAi glc)	
	N2/*acs-5* RNAi	21.2±0.6	27	3.0%	86/120	0.2059	
	N2/*acs-5* RNAi glucose	12.9±0.3	17	-37.4%<br>-2.3%(ctrl RNAi glc)	105/120	*p*<0.0001<br>0.282(ctrl RNAi glc)	
	N2/*lin-41* RNAi	9.7±0.3	13	-53.2%	115/120	*p*<0.0001	
	N2/*lin-41* RNAi glucose	4.3±0.3	7	-79.3%<br>-67.6%(ctrl RNAi glc)	57/62	*p*<0.0001<br>*p*<0.0001(ctrl RNAi glc)	

	N2/*mcm-7* RNAi	17.7±0.6	23	-14.4%	64/100	*p*<0.0001	
	N2/*mcm-7* RNAi glucose	11.3±0.4	13	-45.3%<br>-14.6%(ctrl RNAi glc)	112/120	*p*<0.0001<br>0.0003(ctrl RNAi glc)	
	N2/*kin-10* RNAi	14.9±0.3	17	-27.9%	93/120	*p*<0.0001	
	N2/*kin-10* RNAi glucose	9.2±0.2	11	-55.2%<br>-30.1%(ctrl RNAi glc)	112/120	*p*<0.0001<br>*p*<0.0001(ctrl RNAi glc)	
#2	N2/Control RNAi	19.9±0.6	25		95/120		
	N2/Control RNAi glucose	14.2±0.4	18	-28.6%	105/120	*p*<0.0001	
	N2/*pod-2* RNAi*	20.4±0.6	25	2.3%	69/120	0.7719	
	N2/*pod-2* RNAi glucose*	9.8±0.2	-	-51.0%<br>-31.3%(ctrl RNAi glc)	91/120	*p*<0.0001<br>*p*<0.0001(ctrl RNAi glc)	
	N2/*F56B3.2A* RNAi	14.7±0.3	16	-26.5%	103/120	*p*<0.0001	
	N2/*F56B3.2A* RNAi glucose	6.3±0.3	9	-68.5%<br>-55.9%(ctrl RNAi glc)	111/120	*p*<0.0001<br>*p*<0.0001(ctrl RNAi glc)	
	N2/*acs-5* RNAi	20.1±0.5	23	1.0%	81/120	0.9166	
	N2/*acs-5* RNAi glucose	14.3±0.4	18	-28.2%<br>0.6%(ctrl RNAi glc)	103/120	*p*<0.0001<br>0.7369(ctrl RNAi glc)	
	N2/*lin-41* RNAi	11.2±0.3	14	-43.9%	89/120	*p*<0.0001	

	N2/*lin-41* RNAi glucose	4.8±0.2	9	-75.9%<br>-66.3%(ctrl RNAi glc)	92/120	*p*<0.0001<br>*p*<0.0001(ctrl RNAi glc)	
	N2/*mcm-7* RNAi	15±0.3	18	-25.0%	60/90	*p*<0.0001	
	N2/*mcm-7* RNAi glucose	10.4±0.4	12	-48.0%<br>-27.1%(ctrl RNAi glc)	102/120	*p*<0.0001<br>*p*<0.0001(ctrl RNAi glc)	
	N2/*kin-10* RNAi	15.2±0.3	18	-23.7%	85/120	*p*<0.0001	
	N2/*kin-10* RNAi glucose	11±0.2	14	-45.0%<br>-23.0%(ctrl RNAi glc)	84/120	*p*<0.0001<br>*p*<0.0001(ctrl RNAi glc)	
#1	N2/Control RNAi	20.1±0.4	23		114/150		
	N2/Control RNAi glucose	15.5±0.3	18	-23.1%	112/150	*p*<0.0001	
	N2/*pyp-1* RNAi	18.7±0.4	23	-6.8%	110/150	0.0354	
	N2/*pyp-1* RNAi glucose	15.3±0.2	18	-23.8%<br>-0.9%(ctrl RNAi glc)	119/150	*p*<0.0001<br>0.1344(ctrl RNAi glc)	
#2	N2/Control RNAi	19.9±0.5	25		89/120		
	N2/Control RNAi glucose	16.1±0.3	20	-19.1%	113/150	*p*<0.0001	
	N2/*pyp-1* RNAi	18.6±0.5	23	-6.6%	101/120	0..0644	
	N2/*pyp-1* RNAi glucose	15.3±0.2	18	-22.9%<br>-4.7%(ctrl RNAi glc)	138/150	*p*<0.0001<br>*p*<0.0001(ctrl RNAi glc)	

#1	N2/Control RNAi	18.5±0.4	22		112/150		
	N2/Control RNAi glucose	16.5±0.2	20	-10.9%	145/150	*p*<0.0001	
	N2/*F53B7.3* RNAi	15.6±0.3	20	-16.0%	75/131	*p*<0.0001	
	N2/*F53B7.3* RNAi glucose	9.8±0.2	14	-47.3%<br>-40.8%(ctrl RNAi glc)	123/141	*p*<0.0001<br>*p*<0.0001(ctrl RNAi glc)	
#2	N2/Control RNAi	20.8±0.5	24		109/150		
	N2/Control RNAi glucose	16.9±0.3	20	-18.9%	87/150	*p*<0.0001	
	N2/*F53B7.3* RNAi	15.5±0.5	17	-25.4%	56/60	*p*<0.0001	
	N2/*F53B7.3* RNAi glucose	15.1±0.3	20	-27.4%<br>-10.5%(ctrl RNAi glc)	80/150	*p*<0.0001<br>0.0001(ctrl RNAi glc)	
#1	N2/Control RNAi	20.3±0.4	24		90/100		
	N2/Control RNAi glucose	15.1±0.4	18	-25.8%	34/100	*p*<0.0001	
	N2/*ceh-20* RNAi	22.4±0.3	24	10.0%	89/100	0.0057	
	N2/*ceh-20* RNAi glucose	15.7±0.4	18	-22.9%<br>3.8%(ctrl RNAi glc)	47/100	*p*<0.0001<br>0.2451(ctrl RNAi glc)	
#2	N2/Control RNAi	20.6±0.4	24		106/120		
	N2/Control RNAi glucose	13.1±0.4	16	-36.4%	79/149	*p*<0.0001	
	N2/*ceh-20* RNAi	21±0.3	24	1.8%	121/150	0.9218	
	N2/*ceh-20* RNAi glucose	13.7±0.3	16	-33.8%	117/150	*p*<0.0001	

				4.1%(ctrl RNAi glc)		0.2445(ctrl RNAi glc)	
#1	N2/Control RNAi	23±0.6	29		90/120		
	N2/Control RNAi glucose	11.8±0.3	14	-48.9%	97/120	*p*<0.0001	
	N2/*pod-2* RNAi*	18.5±0.4	22	-19.5%	92/120	*p*<0.0001	
	N2/*pod-2* RNAi glucose*	9.6±0.2	12	-58.2%<br>-18.3%(ctrl RNAi glc)	110/120	*p*<0.0001<br>*p*<0.0001(ctrl RNAi glc)	
	N2/*mel-26* RNAi	22.3±0.6	26	-3.0%	95/120	0.4844	
	N2/*mel-26* RNAi glucose	16.3±0.4	20	-29.0%<br>38.8%(ctrl RNAi glc)	87/120	*p*<0.0001<br>*p*<0.0001(ctrl RNAi glc)	
	N2/*gob-1* RNAi	10.8±0.2	12	-52.8%	94/120	*p*<0.0001	
	N2/*gob-1* RNAi glucose	8±0.1	12	-65.1%<br>-31.7%(ctrl RNAi glc)	97/120	*p*<0.0001<br>*p*<0.0001(ctrl RNAi glc)	
	N2/*M03F4.6* RNAi	16±0.4	18	-30.3%	87/120	*p*<0.0001	
	N2/*M03F4.6* RNAi glucose	8.6±0.2	12	-62.4%<br>-26.6%(ctrl RNAi glc)	54/120	*p*<0.0001<br>*p*<0.0001(ctrl RNAi glc)	
	N2/*dpy-10* RNAi	18.3±0.5	22	-20.5%	77/120	*p*<0.0001	
	N2/*dpy-10* RNAi glucose	12.4±0.3	14	-46.3%<br>5.1%(ctrl RNAi glc)	95/120	*p*<0.0001<br>0.132(ctrl RNAi glc)	

	N2/*cyk-4* RNAi	14.3±0.4	16	-37.8%	63/90	*p*<0.0001	
	N2/*cyk-4* RNAi glucose	14.2±0.5	16	-38.3%<br>20.7%(ctrl RNAi glc)	65/90	*p*<0.0001<br>*p*<0.0001(ctrl RNAi glc)	
#2	N2/Control RNAi	23.5±0.6	26		78/120		
	N2/Control RNAi glucose	15±0.5	18	-36.3%	61/120	*p*<0.0001	
	N2/*pod-2* RNAi*	21.2±0.5	24	-10.0%	81/120	*p*<0.0001	
	N2/*pod-2* RNAi glucose*	10.3±0.1	14	-56.1%<br>-31.1%(ctrl RNAi glc)	69/120	*p*<0.0001<br>*p*<0.0001(ctrl RNAi glc)	
	N2/*mel-26* RNAi	26.6±0.9	35	13.3%	67/120	0.0005	
	N2/*mel-26* RNAi glucose	15.2±0.4	18	-35.5%<br>1.3%(ctrl RNAi glc)	68/120	*p*<0.0001<br>0.633(ctrl RNAi glc)	
	N2/*gob-1* RNAi	12.5±0.2	16	-46.7%	98/120	*p*<0.0001	
	N2/*gob-1* RNAi glucose	10.1±0.2	14	-57.2%<br>-32.8%(ctrl RNAi glc)	54/120	*p*<0.0001<br>*p*<0.0001(ctrl RNAi glc)	
	N2/*M03F4.6* RNAi	17.5±0.4	20	-25.3%	85/120	*p*<0.0001	
	N2/*M03F4.6* RNAi glucose	9.2±0.5	12	-60.8%<br>-38.4%(ctrl RNAi glc)	73/120	*p*<0.0001<br>*p*<0.0001(ctrl RNAi glc)	
	N2/*dpy-10* RNAi	21.5±0.7	26	-8.5%	50/120	0.0578	

	N2/*dpy-10* RNAi glucose	14.6±0.3	18	-37.7%<br>-2.1%(ctrl RNAi glc)	68/120	p<0.0001<br>0.4569(ctrl RNAi glc)	
	N2/*cyk-4* RNAi	15.8±0.4	18	-32.8%	66/120	p<0.0001	
	N2/*cyk-4* RNAi glucose	14±0.4	18	-40.4%<br>-6.5%(ctrl RNAi glc)	84/120	p<0.0001<br>0.1561(ctrl RNAi glc)	
#1	N2/Control RNAi	23.7±0.6	27		48/90		
	N2/Control RNAi glucose	15.7±0.4	19	-33.9%	69/117	p<0.0001	
	N2/*pod-2* RNAi*	20.7±0.6	25	-12.8%	77/117	0.0089	
	N2/*pod-2* RNAi glucose*	10.6±0.3	15	-55.4%<br>-32.5%(ctrl RNAi glc)	69/120	p<0.0001<br>p<0.0001(ctrl RNAi glc)	
	N2/*unc-45* RNAi	17.5±0.8	21	-26.2%	31/77	p<0.0001	
	N2/*unc-45* RNAi glucose	14.9±0.5	17	-37.0%<br>-4.6%(ctrl RNAi glc)	82/120	p<0.0001<br>0.3692(ctrl RNAi glc)	
	N2/*prp-19* RNAi	14±0.4	17	-40.7%	39/57	p<0.0001	
	N2/*prp-19* RNAi glucose	16.1±0.3	19	-32.0%<br>2.9%(ctrl RNAi glc)	100/120	p<0.0001<br>0.8698(ctrl RNAi glc)	
	N2/*aldo-2* RNAi	23±0.6	27	-3.0%	60/120	0.3919	
	N2/*aldo-2* RNAi glucose	16.1±0.3	19	-32.0%	88/120	p<0.0001<br>0.8661(ctrl RNAi	

				2.9%(ctrl RNAi glc)		glc)	
	N2/*T20B12.3* RNAi	18.8±0.7	23	-20.7%	61/108	0.0001	
	N2/*T20B12.3* RNAi glucose	16±0.5	21	-32.5%<br>2.1%(ctrl RNAi glc)	81/116	*p*<0.0001<br>0.2452(ctrl RNAi glc)	
	N2/*ncl-1* RNAi	22.6±0.7	27	-4.6%	49/120	0.5922	
	N2/*ncl-1* RNAi glucose	11.7±0.5	13	-50.4%<br>-25.0%(ctrl RNAi glc)	63/115	*p*<0.0001<br>*p*<0.0001(ctrl RNAi glc)	
#2	N2/Control RNAi	22.2±0.7	26		87/120		
	N2/Control RNAi glucose	12.8±0.4	16	-42.4%	100/120	*p*<0.0001	
	N2/*pod-2* RNAi*	16.9±0.4	20	-23.8%	99/120	*p*<0.0001	
	N2/*pod-2* RNAi glucose*	9.6±0.2	12	-56.6%<br>-24.6%(ctrl RNAi glc)	105/120	*p*<0.0001<br>*p*<0.0001(ctrl RNAi glc)	
	N2/*unc-45* RNAi	14.3±0.5	18	-35.4%	76/100	*p*<0.0001	
	N2/*unc-45* RNAi glucose	11.3±0.4	12	-49.2%<br>-11.8%(ctrl RNAi glc)	107/120	*p*<0.0001<br>0.0108(ctrl RNAi glc)	
	N2/*T10F2.4* RNAi	13.5±0.3	16	-39.1%	72/110	*p*<0.0001	
	N2/*T10F2.4* RNAi glucose	15.9±0.4	18	-28.1%<br>24.9%(ctrl RNAi glc)	108/120	*p*<0.0001<br>*p*<0.0001(ctrl RNAi glc)	

	N2/*aldo-2* RNAi	20.7±0.7	26	-6.4%	72/120	0.139	
	N2/*aldo-2* RNAi glucose	16.9±0.4	20	-23.8%<br>32.3%(ctrl RNAi glc)	110/120	*p*<0.0001<br>*p*<0.0001(ctrl RNAi glc)	
	N2/*T20B12.3* RNAi	16.9±0.6	22	-23.7%	77/120	*p*<0.0001	
	N2/*T20B12.3* RNAi glucose	13.4±0.6	18	-39.3%<br>5.4%(ctrl RNAi glc)	102/120	*p*<0.0001<br>0.22(ctrl RNAi glc)	
	N2/*ncl-1* RNAi	19.8±0.6	24	-10.7%	56/120	0.0039	
	N2/*ncl-1* RNAi glucose	9±0.3	10	-59.5%<br>-29.6%(ctrl RNAi glc)	114/120	*p*<0.0001<br>*p*<0.0001(ctrl RNAi glc)	
#1	N2/Control RNAi	18.5±0.6	23		93/120		
	N2/Control RNAi glucose	12.6±0.4	17	-32.0%	120/120	*p*<0.0001	
	N2/*pod-2* RNAi*	17.7±0.5	21	-4.7%	75/120	0.1194	
	N2/*pod-2* RNAi glucose*	8.9±0.1	13	-52.0%<br>-29.4%(ctrl RNAi glc)	117/120	*p*<0.0001<br>*p*<0.0001(ctrl RNAi glc)	
	N2/*mboa-1* RNAi	19.8±0.6	23	6.9%	82/120	0.2001	
	N2/*mboa-1* RNAi glucose	15.2±0.3	17	-18.0%<br>20.6%(ctrl RNAi glc)	110/120	*p*<0.0001<br>*p*<0.0001(ctrl RNAi glc)	
	N2/*R11A8.7* RNAi	16.3±0.3	19	-12.0%	77/120	0.0002	
	N2/*R11A8.7* RNAi glucose	10.8±0.3	13	-41.7%	118/120	*p*<0.0001	

				-14.3%(ctrl RNAi glc)		0.0002(ctrl RNAi glc)	
	N2/*clc-2* RNAi	19.9±0.8	23	7.5%	69/90	0.0854	
	N2/*clc-2* RNAi glucose	12.4±0.4	15	-33.3%<br>-1.9%(ctrl RNAi glc)	118/120	p<0.0001<br>0.7025(ctrl RNAi glc)	
	N2/*plc-3* RNAi	10.8±0.3	13	-42.0%	40/120	p<0.0001	
	N2/*plc-3* RNAi glucose	7.7±0.2	11	-58.6%<br>-39.0%(ctrl RNAi glc)	120/120	p<0.0001<br>p<0.0001(ctrl RNAi glc)	
#2	N2/Control RNAi	20.3±0.9	25		75/90		
	N2/Control RNAi glucose	13.3±0.4	17	-34.7%	116/120	p<0.0001	
	N2/*pod-2* RNAi*	19.4±0.5	22	-4.5%	79/90	0.1194	
	N2/*pod-2* RNAi glucose*	8.9±0.1	13	-56.1%<br>-32.9%(ctrl RNAi glc)	112/120	p<0.0001<br>p<0.0001(ctrl RNAi glc)	
	N2/*mboa-1* RNAi	18.9±0.5	22	-7.3%	107/120	0.2001	
	N2/*mboa-1* RNAi glucose	15.2±0.3	17	-25.5%<br>14.0%(ctrl RNAi glc)	115/120	p<0.0001<br>p<0.0001(ctrl RNAi glc)	
	N2/*R11A8.7* RNAi	14.8±0.3	17	-27.1%	103/120	0.0002	
	N2/*R11A8.7* RNAi glucose	12.2±0.4	15	-40.1%<br>-8.3%(ctrl RNAi glc)	112/120	p<0.0001<br>0.0002(ctrl RNAi glc)	

	N2/*clc-2* RNAi	21.1±0.8	28	3.7%	93/120	0.0854	
	N2/*clc-2* RNAi glucose	13±0.4	17	-36.2%<br>-2.4%(ctrl RNAi glc)	113/120	*p*<0.0001<br>0.7025(ctrl RNAi glc)	
	N2/*plc-3* RNAi	12.1±0.4	15	-40.6%	36/120	*p*<0.0001	
	N2/*plc-3* RNAi glucose	8.5±0.3	11	-58.0%<br>-35.7%(ctrl RNAi glc)	55/60	*p*<0.0001<br>*p*<0.0001(ctrl RNAi glc)	
#1	N2/Control RNAi	22.8±0.6	26		75/120		
	N2/Control RNAi glucose	13.7±0.3	17	-40.0%	88/120	*p*<0.0001	
	N2/*pod-2* RNAi*	22.8±0.7	28	0.0%	64/90	0.7737	
	N2/*pod-2* RNAi glucose*	11.6±0.2	14	-49.3%<br>-15.5%(ctrl RNAi glc)	93/120	*p*<0.0001<br>*p*<0.0001(ctrl RNAi glc)	
	N2/*ncbp-1* RNAi	13.4±0.3	17	-41.2%	40/90	*p*<0.0001	
	N2/*ncbp-1* RNAi glucose	9.5±0.3	12	-58.4%<br>-30.8%(ctrl RNAi glc)	88/116	*p*<0.0001<br>*p*<0.0001(ctrl RNAi glc)	
	N2/*rgs-3* RNAi	22.1±0.7	26	-3.2%	67/120	0.5958	
	N2/*rgs-3* RNAi glucose	13.9±0.4	17	-39.0%<br>1.7%(ctrl RNAi glc)	89/120	*p*<0.0001<br>0.5271(ctrl RNAi glc)	
	N2/*sox-2* RNAi	16.7±0.4	19	-26.8%	60/120	*p*<0.0001	

	N2/*sox-2* RNAi glucose	13.8±0.3	17	-39.5%<br>0.8%(ctrl RNAi glc)	74/120	$p<0.0001$<br>0.9209(ctrl RNAi glc)	
	N2/*prmt-3* RNAi	21±0.6	26	-8.0%	81/120	0.0628	
	N2/*prmt-3* RNAi glucose	13±0.3	17	-43.1%<br>-5.2%(ctrl RNAi glc)	101/120	$p<0.0001$<br>0.247(ctrl RNAi glc)	
	N2/*top-2* RNAi	16.7±0.5	19	-26.6%	31/63	$p<0.0001$	
	N2/*top-2* RNAi glucose	14.9±0.5	19	-34.8%<br>8.7%(ctrl RNAi glc)	91/120	$p<0.0001$<br>0.0124(ctrl RNAi glc)	
	N2/Control RNAi	25.1±0.8	30		55/120		
	N2/Control RNAi glucose	16.5±0.5	20	-34.2%	44/90	$p<0.0001$	
	N2/*pod-2* RNAi*	23.3±0.8	30	-7.1%	82/120	0.1045	
	N2/*pod-2* RNAi glucose*	12.3±0.4	15	-51.0 %<br>-25.5%(ctrl RNAi glc)	91/120	$p<0.0001$<br>$p<0.0001$(ctrl RNAi glc)	
#2	N2/*ncbp-1* RNAi	12.5±0.4	15	-50.1%	57/98	$p<0.0001$	
	N2/*ncbp-1* RNAi glucose	9±0.3	11	-64.2%<br>-45.6%(ctrl RNAi glc)	81/105	$p<0.0001$<br>$p<0.0001$(ctrl RNAi glc)	
	N2/*rgs-3* RNAi	24.6±1	30	-2.0%	55/120	0.7921	
	N2/*rgs-3* RNAi glucose	18±0.4	22	-28.2%	61/120	$p<0.0001$<br>0.0313(ctrl RNAi	

				9.1%(ctrl RNAi glc)		glc)	
	N2/*sox-2* RNAi	16.2±0.5	20	-35.3%	55/111	*p*<0.0001	
	N2/*sox-2* RNAi glucose	14.8±0.3	17	-41.3%<br>-10.7%(ctrl RNAi glc)	62/120	*p*<0.0001<br>0.0068(ctrl RNAi glc)	
	N2/*prmt-3* RNAi	22.9±0.9	28	-8.7%	59/120	0.1647	
	N2/*prmt-3* RNAi glucose	16.7±0.5	22	-33.3%<br>1.3%(ctrl RNAi glc)	68/120	*p*<0.0001<br>0.7718(ctrl RNAi glc)	
	N2/*top-2* RNAi	16.9±0.6	20	-32.8%	42/69	*p*<0.0001	
	N2/*top-2* RNAi glucose	15.3±0.6	20	-39.0%<br>-7.3%(ctrl RNAi glc)	71/120	*p*<0.0001<br>0.2472(ctrl RNAi glc)	
#1	N2/Control RNAi	25±0.7	28		78/120		
	N2/Control RNAi glucose	15.9±0.5	20	-36.6%	51/120	*p*<0.0001	
	N2/*pod-2* RNAi*	25.5±0.6	30	2.0%	89/120	0.9634	
	N2/*pod-2* RNAi glucose*	12.9±0.3	15	-48.5%<br>-18.8%(ctrl RNAi glc)	55/120	*p*<0.0001<br>*p*<0.0001(ctrl RNAi glc)	
	N2/*ruvb-2* RNAi	18.4±0.5	22	-26.6%	95/120	*p*<0.0001	
	N2/*ruvb-2* RNAi glucose	16.5±0.6	20	-33.9%<br>4.2%(ctrl RNAi glc)	48/120	*p*<0.0001<br>0.2398(ctrl RNAi glc)	

	N2/*rnp-6* RNAi	14.1±0.6	17	-43.6%	28/49	*p*<0.0001	
	N2/*rnp-6* RNAi glucose	13.4±0.3	15	-46.3%<br>-15.3%(ctrl RNAi glc)	76/120	*p*<0.0001<br>0.0002(ctrl RNAi glc)	
	N2/*use-1* RNAi	12.6±0.3	15	-49.5%	60/80	*p*<0.0001	
	N2/*use-1* RNAi glucose	12±0.3	15	-51.9%<br>-24.1%(ctrl RNAi glc)	100/120	*p*<0.0001<br>*p*<0.0001(ctrl RNAi glc)	
	N2/*ztf-19* RNAi	19.1±0.8	22	-23.7%	34/104	*p*<0.0001	
	N2/*ztf-19* RNAi glucose	14.6±0.5	17	-41.6%<br>-7.9%(ctrl RNAi glc)	71/120	*p*<0.0001<br>0.1057(ctrl RNAi glc)	
#2	N2/Control RNAi	21±0.6	25		64/90		
	N2/Control RNAi glucose	16.8±0.4	21	-20.3%	92/120	*p*<0.0001	
	N2/*pod-2* RNAi*	23.4±0.6	29	11.4%	73/120	0.0027	
	N2/*pod-2* RNAi glucose*	15.7±0.4	18	-25.2%<br>-6.1%(ctrl RNAi glc)	100/120	*p*<0.0001<br>0.0851(ctrl RNAi glc)	
	N2/*ruvb-2* RNAi	18.3±0.5	21	-12.8%	56/90	0.0023	
	N2/*ruvb-2* RNAi glucose	14.2±0.3	16	-32.3%<br>-15.1%(ctrl RNAi glc)	57/90	*p*<0.0001<br>*p*<0.0001(ctrl RNAi glc)	
	N2/*rnp-6* RNAi	13.2±0.6	16	-37.3%	21/50	*p*<0.0001	

	N2/*rnp-6* RNAi glucose	11.9±0.2	14	-43.6%<br>-29.2% (ctrl RNAi glc)	73/120	$p<0.0001$<br>$p<0.0001$ (ctrl RNAi glc)	
	N2/*use-1* RNAi	12.7±0.3	16	-39.8%	48/90	$p<0.0001$	
	N2/*use-1* RNAi glucose	11.3±0.2	14	-46.3%<br>-32.6% (ctrl RNAi glc)	51/90	$p<0.0001$<br>$p<0.0001$ (ctrl RNAi glc)	
	N2/*ztf-19* RNAi	16.7±0.6	21	-20.8%	49/90	$p<0.0001$	
	N2/*ztf-19* RNAi glucose	14.7±0.5	18	-30.1%<br>-12.3% (ctrl RNAi glc)	93/120	$p<0.0001$<br>0.0093 (ctrl RNAi glc)	
#1	N2/Control RNAi	21.2±0.6	26		101/120		
	N2/Control RNAi glucose	12.5±0.3	14	-41.1%	85/120	$p<0.0001$	
	N2/*pod-2* RNAi*	22.5±0.7	28	6.3%	75/90	0.3135	
	N2/*pod-2* RNAi glucose*	12±0.3	14	-43.6%<br>-4.2% (ctrl RNAi glc)	103/120	$p<0.0001$<br>$p<0.0001$ (ctrl RNAi glc)	
	N2/*plk-1* RNAi	16.8±0.4	20	-20.9%	80/103	$p<0.0001$	
	N2/*plk-1* RNAi glucose	13.2±0.5	18	-37.8%<br>5.7% (ctrl RNAi glc)	93/120	$p<0.0001$<br>0.1324 (ctrl RNAi glc)	
	N2/*fos-1* RNAi	14.3±0.5	18	-32.7%	88/120	$p<0.0001$	
	N2/*fos-1* RNAi glucose	10.4±0.4	12	-51.0%	95/120	$p<0.0001$<br>0.0002 (ctrl RNAi	

				-16.8%(ctrl RNAi glc)		glc)	
	N2/*T19B10.2* RNAi	18±0.5	20	-15.0%	70/80	0.0002	
	N2/*T19B10.2* RNAi glucose	15.8±0.3	18	-25.5%<br>26.5%(ctrl RNAi glc)	88/120	*p*<0.0001<br>*p*<0.0001(ctrl RNAi glc)	
#2	N2/Control RNAi	25.4±0.8	34		106/120		
	N2/Control RNAi glucose	14.6±0.4	21	-42.5%	113/120	*p*<0.0001	
	N2/*pod-2* RNAi*	24.6±0.8	29	-3.3%	97/120	0.352	
	N2/*pod-2* RNAi glucose*	13.4±0.4	17	-47.2%<br>-8.2%(ctrl RNAi glc)	113/120	*p*<0.0001<br>0.0565(ctrl RNAi glc)	
	N2/*plk-1* RNAi	16.9±0.7	21	-33.3%	65/75	*p*<0.0001	
	N2/*plk-1* RNAi glucose	13.4±0.6	17	-47.4%<br>-8.5%(ctrl RNAi glc)	112/120	*p*<0.0001<br>0.5428(ctrl RNAi glc)	
	N2/*fos-1* RNAi	13.4±0.6	17	-47.3%	94/120	*p*<0.0001	
	N2/*fos-1* RNAi glucose	12.2±0.5	14	-52.0%<br>-16.6%(ctrl RNAi glc)	110/120	*p*<0.0001<br>0.0021(ctrl RNAi glc)	
	N2/*T19B10.2* RNAi	19.2±0.4	24	-24.3%	98/120	*p*<0.0001	
	N2/*T19B10.2* RNAi glucose	11.4±0.4	17	-55.0%<br>-21.8%(ctrl RNAi glc)	116/120	*p*<0.0001<br>*p*<0.0001(ctrl RNAi glc)	

	N2/Control RNAi	22.6±0.7	27		113/120		
	N2/Control RNAi glucose	12.6±0.4	14	-44.2%	117/120	*p*<0.0001	
	N2/*pod-2* RNAi*	23.9±0.5	27	5.9%	106/120	0.705	
	N2/*pod-2* RNAi glucose*	10.3±0.2	12	-54.6%<br>-18.7%(ctrl RNAi glc)	114/120	*p*<0.0001<br>*p*<0.0001(ctrl RNAi glc)	
	N2/*ruvb-1* RNAi	14.3±0.4	16	-36.7%	92/120	*p*<0.0001	
	N2/*ruvb-1* RNAi glucose	13.2±0.5	16	-41.7%<br>4.5%(ctrl RNAi glc)	108/120	*p*<0.0001<br>0.3112(ctrl RNAi glc)	
#1	N2/*lin-59* RNAi	11.2±0.3	14	-50.6%	72/120	*p*<0.0001	
	N2/*lin-59* RNAi glucose	7.4±0.2	10	-67.3%<br>-41.4%(ctrl RNAi glc)	97/120	*p*<0.0001<br>*p*<0.0001(ctrl RNAi glc)	
	N2/*aman-3* RNAi	21.3±0.7	27	-5.6%	100/120	0.1735	
	N2/*aman-3* RNAi glucose	12.5±0.4	15	-44.8%<br>-1.0%(ctrl RNAi glc)	110/120	*p*<0.0001<br>0.9788(ctrl RNAi glc)	
	N2/*Y53F4B.14* RNAi	21.9±0.9	27	-3.2%	81/90	0.9177	
	N2/*Y53F4B.14* RNAi glucose	11.5±0.3	15	-49.0%<br>-8.7%(ctrl RNAi glc)	110/120	*p*<0.0001<br>0.0526(ctrl RNAi glc)	
#2	N2/Control RNAi	23.4±0.7	30		89/120		

	N2/Control RNAi glucose	16.3±0.6	19	-30.5%	69/119	$p<0.0001$	
	N2/*pod-2* RNAi*	23.3±0.7	28	-0.5%	93/120	0.7756	
	N2/*pod-2* RNAi glucose*	15.6±0.4	19	-33.1%<br>-3.8%(ctrl RNAi glc)	39/119	$p<0.0001$<br>0.8692(ctrl RNAi glc)	
	N2/*ruvb-1* RNAi	16.7±0.5	19	-28.7%	90/120	$p<0.0001$	
	N2/*ruvb-1* RNAi glucose	18.1±0.5	21	-22.5%<br>11.5%(ctrl RNAi glc)	69/119	$p<0.0001$<br>0.0204(ctrl RNAi glc)	
	N2/*lin-59* RNAi	12.1±0.4	15	-48.2%	45/120	$p<0.0001$	
	N2/*lin-59* RNAi glucose	12±0.7	15	-48.7%<br>-26.2%(ctrl RNAi glc)	22/117	$p<0.0001$<br>0.0004(ctrl RNAi glc)	
	N2/*aman-3* RNAi	24.4±0.7	28	4.3%	73/120	0.5786	
	N2/*aman-3* RNAi glucose	16.1±0.5	19	-31.4%<br>-1.2%(ctrl RNAi glc)	69/118	$p<0.0001$<br>0.7085(ctrl RNAi glc)	
	N2/*Y53F4B.14* RNAi	22.5±0.7	28	-3.8%	77/120	0.2612	
	N2/*Y53F4B.14* RNAi glucose	16±0.5	19	-31.6%<br>-1.6%(ctrl RNAi glc)	69/120	$p<0.0001$<br>0.5889(ctrl RNAi glc)	
#1	N2/Control RNAi	18.6±0.6	24		102/120		
	N2/Control RNAi glucose	12.6±0.4	14	-32.5%	119/120	$p<0.0001$	

	N2/*pod-2* RNAi*	19±0.6	24	2.3%	108/120	0.7185	
	N2/*pod-2* RNAi glucose*	10.1±0.2	12	-45.7%<br>-19.6% (ctrl RNAi glc)	120/120	*p*<0.0001<br>*p*<0.0001 (ctrl RNAi glc)	
	N2/*F26A10.2* RNAi	13.2±0.4	16	-29.1%	79/102	*p*<0.0001	
	N2/*F26A10.2* RNAi glucose	12.3±0.5	16	-33.9%<br>-2.1% (ctrl RNAi glc)	105/109	*p*<0.0001<br>0.7831 (ctrl RNAi glc)	
	N2/*C50B6.3* RNAi	20.5±0.7	27	10.4%	97/120	0.036	
	N2/*C50B6.3* RNAi glucose	10.9±0.3	12	-41.7%<br>-13.7% (ctrl RNAi glc)	120/120	*p*<0.0001<br>0.0012 (ctrl RNAi glc)	
	N2/*K06B4.4* RNAi	22.1±0.8	27	18.8%	93/120	0.0003	
	N2/*K06B4.4* RNAi glucose	11.6±0.4	14	-37.6%<br>-7.6% (ctrl RNAi glc)	110/120	*p*<0.0001<br>0.1219 (ctrl RNAi glc)	
#2	N2/Control RNAi	21.9±0.7	28		97/120		
	N2/Control RNAi glucose	13.1±0.4	15	-40.2%	117/120	*p*<0.0001	
	N2/*pod-2* RNAi*	22.5±0.7	28	2.7%	103/120	0.6546	
	N2/*pod-2* RNAi glucose*	10.3±0.2	13	-53.0%<br>-21.4% (ctrl RNAi glc)	116/120	*p*<0.0001<br>*p*<0.0001 (ctrl RNAi glc)	
	N2/*F26A10.2* RNAi	13.9±0.5	15	-36.6%	101/120	*p*<0.0001	

	N2/*F26A10.2* RNAi glucose	14.7±0.5	20	-32.9%<br>12.2%(ctrl RNAi glc)	113/120	*p*<0.0001<br>0.0068(ctrl RNAi glc)	
	N2/*C50B6.3* RNAi	20.9±0.6	26	-4.6%	103/120	0.212	
	N2/*C50B6.3* RNAi glucose	13.1±0.4	17	-40.2%<br>0.1%(ctrl RNAi glc)	116/120	*p*<0.0001<br>0.9686(ctrl RNAi glc)	
	N2/*K06B4.4* RNAi	23.6±0.7	30	7.7%	96/120	0.0967	
	N2/*K06B4.4* RNAi glucose	12.2±0.4	13	-44.3%<br>-6.9%(ctrl RNAi glc)	113/120	*p*<0.0001<br>0.0799(ctrl RNAi glc)	
#1	N2/Control RNAi	22.5±0.6	26		105/119		
	N2/Control RNAi glucose	10.8±0.2	14	-51.9%	91/120	*p*<0.0001	
	N2/*pod-2* RNAi*	24.1±0.6	29	6.9%	102/120	0.0973	
	N2/*pod-2* RNAi glucose*	9.4±0.2	12	-58.4%<br>-13.5%(ctrl RNAi glc)	79/90	*p*<0.0001<br>*p*<0.0001(ctrl RNAi glc)	
	N2/*R10D12.7* RNAi	23.3±0.8	29	3.4%	65/80	0.4844	
	N2/*R10D12.7* RNAi glucose	11.3±0.3	14	-49.8%<br>4.4%(ctrl RNAi glc)	97/120	*p*<0.0001<br>0.1646(ctrl RNAi glc)	
	N2/*pmp-3* RNAi	17.9±0.2	22	-20.4%	103/120	*p*<0.0001	
	N2/*pmp-3* RNAi glucose	11.6±0.3	14	-48.4%	100/120	*p*<0.0001<br>0.0545(ctrl RNAi	

				7.3% (ctrl RNAi glc)		glc)	
	N2/*C01G10.1* RNAi	22±0.5	26	-2.2%	93/120	0.4347	
	N2/*C01G10.1* RNAi glucose	10.5±0.2	12	-53.3%<br>-2.9% (ctrl RNAi glc)	102/120	*p*<0.0001<br>0.183 (ctrl RNAi glc)	
#2	N2/Control RNAi	24.8±0.8	32		99/120		
	N2/Control RNAi glucose	13.4±0.4	20	-45.9%	115/120	*p*<0.0001	
	N2/*pod-2* RNAi*	26.9±0.7	32	8.5%	97/120	0.254	
	N2/*pod-2* RNAi glucose*	10.9±0.3	15	-56.1%<br>-18.9% (ctrl RNAi glc)	67/75	*p*<0.0001<br>0.0001 (ctrl RNAi glc)	
	N2/*R10D12.7* RNAi	27±0.8	35	9.0%	97/120	0.0455	
	N2/*R10D12.7* RNAi glucose	14±0.5	20	-43.3%<br>4.8% (ctrl RNAi glc)	114/120	*p*<0.0001<br>0.2468 (ctrl RNAi glc)	
	N2/*pmp-3* RNAi	20.3±0.5	24	-18.0%	113/120	*p*<0.0001	
	N2/*pmp-3* RNAi glucose	15±0.4	20	-39.5%<br>11.9% (ctrl RNAi glc)	109/120	*p*<0.0001<br>0.0141 (ctrl RNAi glc)	
	N2/*C01G10.1* RNAi	24±0.7	30	-3.2%	92/120	0.3487	
	N2/*C01G10.1* RNAi glucose	13.9±0.5	20	-43.9%<br>3.7% (ctrl RNAi glc)	116/120	*p*<0.0001<br>0.3102 (ctrl RNAi glc)	

#1	N2/Control RNAi	17.8±0.3	20		112/120		
	N2/Control RNAi glucose	15.7±0.3	18	-11.8%	70/120	0.0001	
	N2/*lpin-1* RNAi	16.3±0.2	20	-8.1%	111/120	*p*<0.0001	
	N2/*lpin-1* RNAi glucose	9.1±0.2	13	-48.7%<br>-41.9%(ctrl RNAi glc)	80/120	*p*<0.0001<br>*p*<0.0001(ctrl RNAi glc)	
#2	N2/Control RNAi	17.4±0.5	21		62/120		Figure 1B
	N2/Control RNAi glucose	14.7±0.3	17	-15.7%	100/120	*p*<0.0001	Figure 1B
	N2/*lpin-1* RNAi	16.9±0.3	19	-3.2%	116/120	0.0901	Figure 1B
	N2/*lpin-1* RNAi glucose	7.4±0.2	10	-57.7%<br>-49.8%(ctrl RNAi glc)	68/120	*p*<0.0001<br>*p*<0.0001(ctrl RNAi glc)	Figure 1B
#1	N2/Control RNAi	22.6±0.6	25		72/120		
	N2/Control RNAi glucose	13.5±0.4	14	-40.4%	39/120	*p*<0.0001	
	N2/*pod-2* RNAi*	21.5±0.4	25	-4.6%	96/120	0.1349	
	N2/*pod-2* RNAi glucose*	9.9±0.1	10	-55.9%<br>-26.1%(ctrl RNAi glc)	71/120	*p*<0.0001<br>*p*<0.0001(ctrl RNAi glc)	
	N2/*cdk-1* RNAi	16.7±0.2	17	-25.9%	110/120	*p*<0.0001	
	N2/*cdk-1* RNAi glucose	17.4±0.4	21	-23.1%<br>29.0%(ctrl RNAi glc)	59/120	*p*<0.0001<br>*p*<0.0001(ctrl RNAi glc)	

	N2/*him-17* RNAi	21.3±0.4	25	-5.5%	84/120	0.0157	
	N2/*him-17* RNAi glucose	15.4±0.4	17	-31.6%<br>14.7%(ctrl RNAi glc)	52/120	*p*<0.0001<br>0.0013(ctrl RNAi glc)	
	N2/*din-1* RNAi	20.5±0.4	23	-9.1%	79/120	0.0016	
	N2/*din-1* RNAi glucose	17.4±0.5	23	-22.8%<br>29.4%(ctrl RNAi glc)	82/120	*p*<0.0001<br>*p*<0.0001(ctrl RNAi glc)	
	N2/*xbp-1* RNAi	19.2±0.3	21	-14.7%	74/120	*p*<0.0001	
	N2/*xbp-1* RNAi glucose	12.9±0.3	14	-42.7%<br>-3.9%(ctrl RNAi glc)	42/120	*p*<0.0001<br>0.4175(ctrl RNAi glc)	
	N2/*C18D11.3* RNAi	21.8±0.5	25	-3.5%	72/120	0.1833	
	N2/*C18D11.3* RNAi glucose	13.3±0.4	17	-41.2%<br>-1.3%(ctrl RNAi glc)	49/120	*p*<0.0001<br>0.3498(ctrl RNAi glc)	
#2	N2/Control RNAi	23±0.8	27		90/120		
	N2/Control RNAi glucose	16.8±0.5	19	-26.8%	35/120	*p*<0.0001	
	N2/*pod-2* RNAi*	21.9±0.7	27	-5.0%	79/120	0.1922	
	N2/*pod-2* RNAi glucose*	19.7±0.9	25	-14.4%<br>16.9%(ctrl RNAi glc)	68/120	0.0046<br>0.3169(ctrl RNAi glc)	
	N2/*cdk-1* RNAi	16.7±0.4	19	-27.5%	106/120	*p*<0.0001	

	N2/*cdk-1* RNAi glucose	18.9±0.6	23	-17.9%<br>12.1%(ctrl RNAi glc)	34/85	0.0172<br>0.0092(ctrl RNAi glc)	
	N2/*him-17* RNAi	22.2±0.7	27	-3.5%	89/120	0.2707	
	N2/*him-17* RNAi glucose	17.3±0.3	19	-24.7%<br>2.9%(ctrl RNAi glc)	58/120	*p*<0.0001<br>0.2801(ctrl RNAi glc)	
	N2/*din-1* RNAi	21.1±0.6	25	-8.4%	111/120	0.0231	
	N2/*din-1* RNAi glucose	21.2±0.4	25	-7.9%<br>25.8%(ctrl RNAi glc)	63/120	0.1704<br>*p*<0.0001(ctrl RNAi glc)	
	N2/*xbp-1* RNAi	20.5±0.6	21	-10.7%	105/120	0.0148	
	N2/*xbp-1* RNAi glucose	14±0.2	15	-39.0%<br>-16.7%(ctrl RNAi glc)	58/115	*p*<0.0001<br>*p*<0.0001(ctrl RNAi glc)	
	N2/*C18D11.3* RNAi	24.5±0.7	30	6.5%	93/120	0.2401	
	N2/*C18D11.3* RNAi glucose	16.2±0.4	19	-29.4%<br>-3.6%(ctrl RNAi glc)	40/120	*p*<0.0001<br>0.3333(ctrl RNAi glc)	
#1	N2/Control RNAi	22.4±0.7	28		101/120		
	N2/Control RNAi glucose	15.5±0.6	20	-30.7%	55/120	*p*<0.0001	
	N2/*snn-1* RNAi	22.6±0.6	26	0.9%	70/120	0.5325	
	N2/*snn-1* RNAi glucose	16.1±0.4	18	-28.2%	55/120	*p*<0.0001<br>0.4927(ctrl RNAi	

				3.6%(ctrl RNAi glc)		glc)	
	N2/*mut-15* RNAi	22.9±0.7	26	2.1%	71/120	0.7752	
	N2/*mut-15* RNAi glucose	15.5±0.4	18	-30.7%<br>0.0%	61/120	*p*<0.0001<br>0.7535(ctrl RNAi glc)	
	N2/*lpr-6* RNAi	24.2±0.7	28	8.1%	104/120	0.0563	
	N2/*lpr-6* RNAi glucose	17.1±0.4	20	-23.5%<br>10.4%	73/120	*p*<0.0001<br>0.0286(ctrl RNAi glc)	
	N2/*lips-2* RNAi	21.3±0.5	24	-4.7%	67/120	0.0133	
	N2/*lips-2* RNAi glucose	15.1±0.5	18	-32.6%<br>-2.8%(ctrl RNAi glc)	53/120	*p*<0.0001<br>0.6462(ctrl RNAi glc)	
	N2/Control RNAi	21.8±0.7	27		68/90		
	N2/Control RNAi glucose	17.3±0.4	21	-20.7%	54/120	*p*<0.0001	
	N2/*snn-1* RNAi	21.9±0.8	27	0.2%	80/120	0.5854	
#2	N2/*snn-1* RNAi glucose	16.5±0.3	19	-24.6%<br>-4.9%(ctrl RNAi glc)	57/110	*p*<0.0001<br>0.0291(ctrl RNAi glc)	
	N2/*mut-15* RNAi	22.1±0.7	25	1.1%	44/60	0.8766	
	N2/*mut-15* RNAi glucose	17.2±0.5	19	-21.2%<br>-0.6%(ctrl RNAi glc)	25/90	0.0004<br>0.652(ctrl RNAi glc)	

	N2/*lpr-6* RNAi	25.8±0.9	30	18.0%	68/120	0.0002	
	N2/*lpr-6* RNAi glucose	16.6±0.3	19	-24.2%<br>-4.4%(ctrl RNAi glc)	56/120	*p*<0.0001<br>0.0332(ctrl RNAi glc)	
	N2/*lips-2* RNAi	20.3±0.7	23	-7.1%	42/90	0.042	
	N2/*lips-2* RNAi glucose	18.1±0.6	21	-17.1%<br>4.6%(ctrl RNAi glc)	32/90	0.0024<br>0.1708(ctrl RNAi glc)	
#1	N2/Control RNAi	20.9±0.6	26		70/90		Figure 1C
	N2/Control RNAi glucose	16.5±0.4	19	-21.0%	61/120	*p*<0.0001	Figure 1C
	N2/*pod-2* RNAi*	19±0.4	24	-9.0%	88/120	0.001	
	N2/*pod-2* RNAi glucose*	10.9±0.2	13	-47.7%<br>-33.8%(ctrl RNAi glc)	59/120	*p*<0.0001<br>*p*<0.0001(ctrl RNAi glc)	
	N2/*cyp-42A1* RNAi	15.4±0.3	17	-26.2%	95/120	*p*<0.0001	Figure 1C
	N2/*cyp-42A1* RNAi glucose	2.4±0.1	2	-88.6%<br>-85.5%(ctrl RNAi glc)	120/120	*p*<0.0001<br>*p*<0.0001(ctrl RNAi glc)	Figure 1C
	N2/*cit-1.2* RNAi	19.7±0.4	23	-5.6%	89/120	0.0058	
	N2/*cit-1.2* RNAi glucose	17.8±0.4	21	-14.8%<br>7.8%(ctrl RNAi glc)	67/120	*p*<0.0001<br>0.0171(ctrl RNAi glc)	

#2	N2/Control RNAi	22.5±0.5	26		115/120		
	N2/Control RNAi glucose	14.2±0.4	20	-37.0%	110/120	*p*<0.0001	
	N2/*pod-2* RNAi*	19.1±0.5	22	-15.1%	112/120	*p*<0.0001	
	N2/*pod-2* RNAi glucose*	10.4±0.2	13	-54.0%<br>-27.0%(ctrl RNAi glc)	110/120	*p*<0.0001<br>*p*<0.0001(ctrl RNAi glc)	
	N2/*cyp-42A1* RNAi	18.8±0.4	22	-16.7%	118/120	*p*<0.0001	
	N2/*cyp-42A1* RNAi glucose	2.9±0.1	4	-87.3%<br>-79.9%(ctrl RNAi glc)	120/120	*p*<0.0001<br>*p*<0.0001(ctrl RNAi glc)	
	N2/*cit-1.2* RNAi	20.5±0.4	23	-9.1%	112/120	0.0001	
	N2/*cit-1.2* RNAi glucose	16.1±0.4	19	-28.5%<br>13.5%(ctrl RNAi glc)	119/120	*p*<0.0001<br>0.001(ctrl RNAi glc)	
#1	N2/Control RNAi	19.9±0.6	24		101/120		Figure 1D-H
	N2/Control RNAi glucose	16.7±0.6	22	-16.1%	58/120	*p*<0.0001	Figure 1D-H
	N2/*plk-1* RNAi	14.5±0.2	15	-27.1%	89/120	*p*<0.0001	Figure 1D
	N2/*plk-1* RNAi glucose	15.1±0.5	19	-24.1%<br>-9.5%(ctrl RNAi glc)	61/120	*p*<0.0001<br>0.0501(ctrl RNAi glc)	Figure 1D
	N2/*T20B12.3* RNAi	13.7±0.3	15	-31.3%	82/120	*p*<0.0001	Figure 1E
	N2/*T20B12.3* RNAi glucose	17.4±0.7	22	-12.8%<br>4.0%(ctrl RNAi glc)	52/120	0.0014<br>0.4475(ctrl RNAi	Figure 1E

						glc)	
	N2/*sox-2* RNAi	13.8±0.3	15	-30.5%	81/120	*p*<0.0001	
	N2/*sox-2* RNAi glucose	14.1±0.7	17	-29.2%<br>-15.6%(ctrl RNAi glc)	36/120	*p*<0.0001<br>0.006(ctrl RNAi glc)	
	N2/*top-2* RNAi	12.2±0.2	13	-38.8%	93/120	*p*<0.0001	Figure 1F
	N2/*top-2* RNAi glucose	14.7±0.5	19	-26.1%<br>-11.9%(ctrl RNAi glc)	61/120	*p*<0.0001<br>0.0086(ctrl RNAi glc)	Figure 1F
	N2/*ruvb-1* RNAi	12.8±0.3	15	-35.7%	100/120	*p*<0.0001	Figure 1G
	N2/*ruvb-1* RNAi glucose	16.1±0.4	19	-19.3%<br>-3.8%(ctrl RNAi glc)	70/120	*p*<0.0001<br>0.3699(ctrl RNAi glc)	Figure 1G
	N2/*cdk-1* RNAi	13.5±0.2	15	-32.4%	109/120	*p*<0.0001	Figure 1H
	N2/*cdk-1* RNAi glucose	15.2±0.3	17	-23.9%<br>-9.2%(ctrl RNAi glc)	68/120	*p*<0.0001<br>0.0768(ctrl RNAi glc)	Figure 1H
#2	N2/Control RNAi	19.2±0.7	24		81/90		
	N2/Control RNAi glucose	16.7±0.8	22	-12.7%	45/120	0.0184	
	N2/*plk-1* RNAi	12.2±0.3	13	-36.5%	95/120	*p*<0.0001	
	N2/*plk-1* RNAi glucose	17.3±0.8	22	-9.8%<br>3.4%(ctrl RNAi glc)	29/85	0.0916<br>0.6884(ctrl RNAi glc)	

	N2/*T20B12.3* RNAi	13.2±0.4	17	-31.1%	76/90	$p<0.0001$	
	N2/*T20B12.3* RNAi glucose	17.5±0.6	22	-8.8%<br>4.5%(ctrl RNAi glc)	63/120	0.0599<br>0.5234(ctrl RNAi glc)	
	N2/*sox-2* RNAi	12.2±0.3	14	-36.3%	88/105	$p<0.0001$	
	N2/*sox-2* RNAi glucose	15.4±0.9	22	-19.4%<br>-7.6%(ctrl RNAi glc)	24/80	0.0053<br>0.3701(ctrl RNAi glc)	
	N2/*top-2* RNAi	12.3±0.3	13	-35.6%	54/75	$p<0.0001$	
	N2/*top-2* RNAi glucose	14.3±0.6	18	-25.2%<br>-14.3%(ctrl RNAi glc)	57/120	$p<0.0001$<br>0.0246(ctrl RNAi glc)	
	N2/*ruvb-1* RNAi	12.9±0.3	14	-32.8%	105/120	$p<0.0001$	
	N2/*ruvb-1* RNAi glucose	13.8±0.5	17	-27.7%<br>-17.2%(ctrl RNAi glc)	84/120	$p<0.0001$<br>0.0019(ctrl RNAi glc)	
	N2/*cdk-1* RNAi	13.4±0.3	14	-30.2%	83/90	$p<0.0001$	
	N2/*cdk-1* RNAi glucose	17±0.5	18	-11.3%<br>1.6%(ctrl RNAi glc)	49/120	0.2368<br>0.2925(ctrl RNAi glc)	
#1	N2/Control RNAi	19.9±0.6	25		71/120		Figure 1I
	N2/Control RNAi glucose	16.5±0.4	19	-17.2%	55/120	0.0001	Figure 1I
	N2/*cyk-4* RNAi	14.2±0.3	17	-28.3%	99/120	$p<0.0001$	Figure 1I

	N2/*cyk-4* RNAi glucose	15.9±0.3	17	-20.1%<br>-3.6%[ctrl RNAi glc]	86/120	*p*<0.0001<br>0.0264[ctrl RNAi glc]	Figure 1I
	N2/*F26A10.2* RNAi	14.3±0.3	17	-28.0%	91/120	*p*<0.0001	
	N2/*F26A10.2* RNAi glucose	14.3±0.6	17	-27.9%<br>-12.9%[ctrl RNAi glc]	46/120	*p*<0.0001<br>0.0045[ctrl RNAi glc]	
	N2/*din-1* RNAi	17.7±0.7	21	-11.1%	92/120	0.0721	
	N2/*din-1* RNAi glucose	18.4±0.5	21	-7.6%<br>11.6%[ctrl RNAi glc]	65/120	0.0087<br>0.0008[ctrl RNAi glc]	
	N2/*prp-19* RNAi	13.5±0.3	14	-31.9%	104/120	*p*<0.0001	
	N2/*prp-19* RNAi glucose	15.7±0.3	17	-20.8%<br>-4.4%[ctrl RNAi glc]	68/120	*p*<0.0001<br>0.0497[ctrl RNAi glc]	
	N2/*pmp-3* RNAi	20.4±0.5	25	2.8%	99/120	0.5689	
	N2/*pmp-3* RNAi glucose	15.5±0.4	19	-21.8%<br>-5.5%[ctrl RNAi glc]	66/120	*p*<0.0001<br>0.1532[ctrl RNAi glc]	
#2	N2/Control RNAi	19.2±0.5	25		100/120		Figure 1J
	N2/Control RNAi glucose	13.7±0.3	16	-28.9%	72/120	*p*<0.0001	Figure 1J
	N2/*cyk-4* RNAi	12.7±0.3	15	-33.9%	80/90	*p*<0.0001	
	N2/*cyk-4* RNAi glucose	15.3±0.4	18	-20.6%	49/80	*p*<0.0001<br>0.0062[ctrl RNAi	

				11.6%$^{\text{(ctrl RNAi glc)}}$		glc)	
	N2/*F26A10.2* RNAi	11.4±0.4	12	-40.7%	100/120	$p<0.0001$	
	N2/*F26A10.2* RNAi glucose	14.5±0.5	18	-24.4%<br>6.3%$^{\text{(ctrl RNAi glc)}}$	55/120	$p<0.0001$<br>0.158$^{\text{(ctrl RNAi glc)}}$	
	N2/*din-1* RNAi	20.3±0.6	25	5.8%	103/120	0.0213	
	N2/*din-1* RNAi glucose	15.3±0.6	20	-20.2%<br>12.2%$^{\text{(ctrl RNAi glc)}}$	78/120	$p<0.0001$<br>0.0295$^{\text{(ctrl RNAi glc)}}$	
	N2/*prp-19* RNAi	12.1±0.2	12	-37.0%	84/100	$p<0.0001$	Figure 1J
	N2/*prp-19* RNAi glucose	15.3±0.4	18	-20.4%<br>11.9%$^{\text{(ctrl RNAi glc)}}$	79/120	$p<0.0001$<br>0.0006$^{\text{(ctrl RNAi glc)}}$	Figure 1J
	N2/*pmp-3* RNAi	18.5±0.4	22	-3.8%	106/120	0.182	
	N2/*pmp-3* RNAi glucose	15.5±0.4	18	-19.2%<br>13.6%$^{\text{(ctrl RNAi glc)}}$	85/120	$p<0.0001$<br>0.0001$^{\text{(ctrl RNAi glc)}}$	
	N2/Control RNAi	20.2±0.4	24		113/125		
	N2/Control RNAi glucose	17.3±0.6	20	-14.3%	53/125	$p<0.0001$	
#1	N2/*cyp-42A1* RNAi	12.8±0.2	14	-36.6%	109/120	$p<0.0001$	
	N2/*cyp-42A1* RNAi glucose	2.2±0.1	2	-89.1%<br>-87.3%$^{\text{(ctrl RNAi glc)}}$	120/120	$p<0.0001$<br>$p<0.0001^{\text{(ctrl RNAi glc)}}$	

#2	N2/Control RNAi	19.3±0.4	22		77/150		
	N2/Control RNAi glucose	15.3±0.6	21	-20.4%	73/150	*p*<0.0001	
	N2/*cyp-42A1* RNAi	12±0.3	14	-37.6%	112/150	*p*<0.0001	
	N2/*cyp-42A1* RNAi glucose	2.6±0.1	4	-86.6%<br>-83.1% (ctrl RNAi glc)	166/167	*p*<0.0001<br>*p*<0.0001(ctrl RNAi glc)	

Lifespan data from the same experimental sets were indicated by bold lines and biological repeats were separated by bold dashed lines and stating trial numbers.

All *p* values were calculated by using log-rank test.

Percent change (%) and *p* values indicate comparison of each condition with control RNAi on control diets within the same experimental dataset.

ctrl RNAi glc: percent change (%) and *p* values were calculated by comparing with control RNAi on glucose diets within the same experimental dataset.

* indicates experimental set of *pod-2* RNAi, which was used as a positive control that specifically decreased lifespan under glucose-rich conditions (Lee et al., 2015).

‘Figures in text’ indicates lifespan dataset that was used in designated Figures.

Table S3. **The list of *fat-5p::fat-5::gfp* enhancer RNAi clones.**

Gene ID	Gene name	Gene description	Average score (6X repeats)
T20B12.8	***hmg-4***	**FACT (facilitates chromatin transcription) complex subunit SSRP1**	**2.5**
C31E10.7	***cybt-5.1***	**Cytochrome b5 ortholog**	**2.1**
T26G10.1	*T26G10.1*	Putative ATP-dependent RNA helicase	2.1
C17A2.8	*nhr-72*	Nuclear receptor family	2.1
C46A5.8	*rdl-1*	Human RD3L (retinal degeneration 3-like) and RD3 ortholog	1.8
C29E4.2	*kle-2*	Human NCAPH2 (non-SMC condensin II complex subunit H2) ortholog	1.8
T25G3.3	***T25G3.3***	***S. cerevisiae* NMD3 ortholog**	**1.7**
F43G9.1	***idha-1***	**Probable isocitrate dehydrogenase subunit alpha**	**1.6**
T19C9.3	*srh-252*	Serpentine receptor, class H	1.6
T23B5.1	*prmt-9*	Human PRMT9 (protein arginine methyltransferase 9) and PRMT7 ortholog	1.5
H37A05.1	***lpin-1***	**Phosphatidic acid phosphatase Lipin 1, Lipin 2, and Lipin 3**	**1.4**
K04C1.1	*srg-37*	G-protein coupled receptor (GPCR) of the serpentine receptor class g	1.2
K07A1.12	*lin-53*	Class B synMuv protein containing a 7 WD-repeat similar to the mammalian homolog RbAp48	1.2
F26A3.2	***ncbp-2***	**Nuclear cap-binding protein subunit 2**	**1.1**
E04A4.4	***hoe-1***	**Two isoforms of a putative metal-dependent hydrolase orthologous to human ELAC2**	**1.0**

K04G11.2	*sel-7*	Suppressor/enhancer of Lin-12	0.9
C32E12.4	*C32E12.4*	Enriched in muscle cell and in the PVD, OLL, and AFD neurons	0.8
T08G11.5	*unc-29*	Neuronal acetylcholine receptor subunit alpha-4	0.7

Enhancer RNAi clones targeting nineteen genes were sorted in descending order by average scores of six replicates from the liquid culture system (Figure S4A, step II).

Seven enhancer RNAi clones that increased *fat-5p::fat-5::gfp* expression on solid media were highlighted in bold (Figure S4A, step III).

Table S4. **The list of *fat-5p::fat-5::gfp* suppressor RNAi clones.**

**Gene ID**	**Gene name**	**Gene description**	**Average score (6X repeats)**
R12B2.5	***mdt-15***	**Mediator subunit orthologous to human MED15**	**-2.6**
F35H10.4	***vha-5***	**V-type proton ATPase subunit a**	**-2.5**
C50C3.6	*prp-8*	Pre-mRNA-splicing factor 8 homolog	-2.5
K10C3.6A	***nhr-49***	**HNF4 (hepatocyte nuclear factor 4) family of NHRs**	**-2.5**
ZK652.1	*snr-5*	Probable small nuclear ribonucleoprotein F	-2.2
B0412.4	*rps-29*	Human RPS29 (ribosomal protein S29) ortholog	-2.2
C14C10.3	*ril-2*	RNAi-Induced longevity	-2.0
Y53G8AR.9	***Y53G8AR.9***	**Human ZC3H10 (zinc finger CCCH-type containing 10) ortholog**	**-1.9**
K09C8.5	***pxn-2***	**Human PXDN (peroxidasin) ortholog**	**-1.9**

F25B4.6	*hmgs-1*	Human 3-hydroxy-3-methylglutaryl CoA synthase ortholog	-1.7
F25B4.7	*F25B4.7*	Human SLC25A31 (solute carrier family 25 member 31) ortholog	-1.7
R02D3.4	*ints-13*	Human INTS13 (integrator complex subunit 13) ortholog	-1.7
Y53G8AR.3	***ral-1***	**Human RAS like proto-oncogene B and RAS like proto-oncogene A ortholog**	**-1.7**
F57B9.3	***F57B9.3***	**Human EIF4A1 (eukaryotic translation initiation factor 4A1) and EIF4A2 ortholog**	**-1.7**
C01H6.5	*nhr-23*	Nuclear receptor ROR-beta	-1.6
F53B1.7	*gpa-5*	GNA12 (G protein subunit alpha 12) and GNA13 (G protein subunit alpha 13)	-1.5
Y53G8AR.7	*Y53G8AR.7*	Human MFSD8 (major facilitator superfamily domain containing 8) ortholog	-1.5
C12D8.12	*str-182*	Seven transmembrane receptor	-1.3
C34G6.3	***C34G6.3***	**C34G6.3 is enriched in the germ line and male**	**-1.3**
C28G1.4	*C28G1.4*	Human ZNF142 (zinc finger protein 142) ortholog	-1.3
F47B10.2	*haly-1*	Histidine ammonia lyase	-1.3
W03G11.1	*col-181*	Collagen	-1.3
F29G6.2	*ccdc-149*	Human CCDC149 (coiled-coil domain containing 149) ortholog	-1.3
R53.4	*R53.4*	Putative ATP synthase subunit f, mitochondrial	-1.3
Y69H2.2	***egas-3***	**Human SCNN (Sodium channels epithelial) family ortholog**	**-1.3**
F18A1.3A	*lir-1*	LIR-1C protein; LIn-26 related	-1.3

Y55F3BR.6	*Y55F3BR.6*	Human HSPB6 (heat shock protein family B (small) member 6) ortholog	-1.2
W01A11.4	*lec-10*	Galectin	-1.2
Y79H2A.11	*zyg-8*	Human DCLK1 (doublecortin like kinase 1) ortholog	-1.2
Y111B2A.11	*epc-1*	Human EPC1 (enhancer of polycomb 1) and EPC2 ortholog	-1.2
F32D8.9	*spp-16*	Saposin-like protein family	-1.2
K12G11.3	***sodh-1***	**Alcohol dehydrogenase 1**	**-1.2**
R07B1.1	*vab-15*	Homeobox protein VAB-15	-1.2
F21G4.3	***F21G4.3***	**F21G4.3 is enriched in the amphid sheath cell**	**-1.2.**
C02B8.5	***frpr-1***	**Homolog of the functionally active Fmrf Receptor (FR) of *D. melanogaster***	**-1.1**
ZK856.5	*ZK856.5*	Human SMPDL3A (sphingomyelin phosphodiesterase acid like 3A) and SMPDL3B ortholog	-1.1
B0496.14	***B0496.14***	**Non-coding RNA**	**-1.1**
F35H10.10	*his-30*	Histone H2A	-1.1

Suppressor RNAi clones targeting thirty eight genes were listed in ascending order by average scores of 6 replicates from the liquid culture system (Figure S4A, Step II).

Thirteen suppressor RNAi clones that decreased *fat-5p::fat-5::gfp* expression on solid media were highlighted in bold (Figure S4A, Step III). Gray boxes indicate that RNAi bacteria targeting multiple genes, which were confirmed through sequencing.

Table S5. Levels of fatty acids measured by using gas chromatography/mass spectrometry.

**Fatty acids**	**Conditions**	**% Fatty acids in total lipids±SEM**	***p*-values**	**log2(fold change)±SEM in Figure 5E**
Myristic acid (14:0)	Control RNAi	0.8±0.0		
	lpin-1 RNAi	0.4±0.0	0.0007	
	Control RNAi glucose	1.5±0.0	0.0001	-1.1±0.1
	lpin-1 RNAi glucose	1.1±0.0	0.0036<br>0.0002(ctrl RNAi glc)	1.6±0.1
7-Tetradecenoic acid (14:1 n-7)	Control RNAi	0.4±0.0		
	lpin-1 RNAi	1.5±1.3	0.4508	
	Control RNAi glucose	1.6±1.2	0.3397	0.2±1.7
	lpin-1 RNAi glucose	1.6±1.3	0.3842<br>0.9732(ctrl RNAi glc)	0.9±0.5
Iso-C15 fatty acid (15:iso)	Control RNAi	3.6±0.2		
	lpin-1 RNAi	2.6±1.1	0.4243	
	Control RNAi glucose	2.6±1.1	0.4033	-1.1±1.2
	lpin-1 RNAi glucose	2.8±1.2	0.5412<br>0.9226(ctrl RNAi glc)	0.0±0.1
Pentadecanoic acid (15:0)	Control RNAi	0.6±0.0		
	lpin-1 RNAi	0.5±0.2	0.4946	
	Control RNAi glucose	0.5±0.0	0.0564	-0.5±0.5
	lpin-1 RNAi glucose	0.4±0.1	0.0898<br>0.2687(ctrl RNAi glc)	-0.3±0.1
Palmitic acid	Control RNAi	3.7±0.1		

(16:0)	*lpin-1* RNAi	1.8±0.1	p<0.0001	
	Control RNAi glucose	7.0±0.218	0.0001	-1.0±0.0
	lpin-1 RNAi glucose	4.5±0.1	0.0034<br>0.0003 (ctrl RNAi glc)	1.3±0.1
Cis-7 hexadecenoic acid (16:1 n-9)	Control RNAi	0.9±0.0		
	lpin-1 RNAi	0.6±0.1	0.0242	
	Control RNAi glucose	1.1±0.1	0.0548	-0.5±0.1
	lpin-1 RNAi glucose	0.8±0.1	0.6629<br>0.0755 (ctrl RNAi glc)	0.4±0.2
Palmitoleic acid (16:1 n-7)	Control RNAi	2.1±0.1		
	lpin-1 RNAi	2.4±0.1	0.0504	
	Control RNAi glucose	4.3±0.4	0.0043	0.2±0.0
	lpin-1 RNAi glucose	4.3±0.3	0.0032<br>0.8737 (ctrl RNAi glc)	0.8±0.2
Iso-C17 fatty acid (17:iso)	Control RNAi	4.3±0.2		
	lpin-1 RNAi	7.1±0.2	0.0007	
	Control RNAi glucose	3.0±0.1	0.0030	0.7±0.0
	lpin-1 RNAi glucose	5.0±0.2	0.0492<br>0.0006 (ctrl RNAi glc)	-0.5±0.1
Cyclic C17 fatty acid (17:cyc)	Control RNAi	16.4±0.1		
	lpin-1 RNAi	10.2±0.3	p<0.0001	
	Control RNAi glucose	17.4±0.2	0.0064	-0.7±0.0
	lpin-1 RNAi glucose	13.1±0.8	0.0144<br>0.0060 (ctrl RNAi	0.4±0.1

			glc)	
Stearic acid (18:0)	Control RNAi	5.1±0.0		
	lpin-1 RNAi	3.0±0.0	p<0.0001	
	Control RNAi glucose	4.8±0.0	0.0115	-0.8±0.0
	lpin-1 RNAi glucose	3.7±0.1	0.0002<br>0.0006(ctrl RNAi glc)	0.3±0.1
Oleic acid, (18:1 n-9)	Control RNAi	3.2±0.1		
	lpin-1 RNAi	1.9±0.1	0.0005	
	Control RNAi glucose	3.6±0.1	0.0522	-0.7±0.1
	lpin-1 RNAi glucose	3.4±0.2	0.3915<br>0.3723(ctrl RNAi glc)	0.8±0.0
Vaccenic acid (18:1 n-7)	Control RNAi	10.6±0.2		
	lpin-1 RNAi	15.3±0.2	p<0.0001	
	Control RNAi glucose	12.5±0.8	0.0839	0.5±0.0
	lpin-1 RNAi glucose	16.5±1.0	0.0051<br>0.0391(ctrl RNAi glc)	0.1±0.1
Ricinoleic acid (18:1OH)	Control RNAi	2.0±0.1		
	lpin-1 RNAi	1.7±0.1	0.0825	
	Control RNAi glucose	2.7±0.2	0.0435	-0.3±0.1
	lpin-1 RNAi glucose	2.2±0.2	0.4980<br>0.1946(ctrl RNAi glc)	0.4±0.1
Linoleic acid (18:2n-6)	Control RNAi	7.8±0.2		
	lpin-1 RNAi	9.6±0.2	0.0036	
	Control RNAi glucose	6.8±0.2	0.0223	0.3±0.1

	lpin-1 RNAi glucose	8.6±0.2	0.0366<br>0.0040(ctrl RNAi glc)	-0.1±0.1
γ-Linolenic acid (18:3 n-6)	Control RNAi	1.2±0.0		
	lpin-1 RNAi	1.0±0.0	0.0022	
	Control RNAi glucose	1.0±0.0	0.0017	-0.2±0.0
	lpin-1 RNAi glucose	1.0±0.0	0.0029<br>0.9131(ctrl RNAi glc)	0.0±0.0
Cyclic C19 fatty acid (19:cyc)	Control RNAi	5.4±0.1		
	lpin-1 RNAi	6.5±0.3	0.0139	
	Control RNAi glucose	4.6±0.4	0.1468	0.3±0.0
	lpin-1 RNAi glucose	5.0±0.4	0.4710<br>0.4653(ctrl RNAi glc)	-0.4±0.1
Dihomo-γ-linolenic acid (20:3n-6)	Control RNAi	2.5±0.1		
	lpin-1 RNAi	3.4±0.0	0.0007	
	Control RNAi glucose	1.9±0.1	0.0077	0.5±0.0
	lpin-1 RNAi glucose	2.3±0.0	0.1784<br>0.0060(ctrl RNAi glc)	-0.6±0.0
Eicosatetraenoic acid (20:4 n-3)	Control RNAi	1.3±0.0		
	lpin-1 RNAi	3.9±0.2	0.0003	
	Control RNAi glucose	0.9±0.0	0.0008	1.6±0.1
	lpin-1 RNAi glucose	2.1±0.1	0.0008<br>0.0002(ctrl RNAi glc)	-0.8±0.1
Arachidonic acid	Control RNAi	3.2±0.2		

(20:4n-6)	*lpin-1* RNAi	2.0±0.1	0.0025	
	Control RNAi glucose	2.5±0.0	0.0155	-0.7±0.1
	lpin-1 RNAi glucose	1.9±0.0	0.0016<br>0.0003(ctrl RNAi glc)	-0.1±0.1
Eicosapentaenoic acid<br>20:5 n-3	Control RNAi	12.4±0.1		
	lpin-1 RNAi	10.9±0.3	0.0071	
	Control RNAi glucose	10.5±0.2	0.0004	-0.2±0.0
	lpin-1 RNAi glucose	10.2±0.1	0.0001<br>0.2271(ctrl RNAi glc)	-0.1±0.0

The levels of fatty acids (%) indicate the averages of proportions of the specific fatty acids in total lipids.

Standard error of mean (SEM) was calculated from three biological replicates.

All *p* values were calculated by using two-tailed Student's *t*-test.

p values show comparison of each condition with control RNAi on control diets from triplicates.

ctrl RNAi glc: *p* values were calculated by comparing with control RNAi on glucose diets from triplicates.

Fold change in log2 scale for *lpin-1* RNAi was calculated by dividing the levels of fatty acids (%) in *lpin-1* RNAi-treated worms on control diets by those in control RNAi-treated worms on control diets.

Fold change in log2 scale for *lpin-1* RNAi glucose was calculated by dividing the levels of fatty acids (%) in *lpin-1* RNAi-treated worms on glucose diets by those in *lpin-1* RNAi-treated worms on control diets.

Bold dashed lines separate individual fatty acids.

Table S6. Statistical analysis and additional repeats of lifespan assays upon feeding with fatty acids.

Trial	Strain/treatment (fatty acid(s))	Mean lifespan ±SEM (days)	75th percentile	% change[Δ]	Number of animals that died/total	*p* value vs. control	Figures in text
#1	N2/control RNAi glucose	11.7±0.3	13		83/90		Figure 6A
	N2/control RNAi glucose (myristic acid (14:0) + palmitic acid (16:0))	9.6±0.3	11	-18.4%	115/117	$p<0.0001$	Figure 6A
	N2/*lpin-1* RNAi glucose	9.7±0.2	11	-17.1%	115/120	$p<0.0001$	Figure 6B
	N2/*lpin-1* RNAi glucose (myristic acid (14:0) + palmitic acid (16:0))	7.3±0.2	8	-38.2%<br>-25%(*lpin-1* RNAi glc)	114/120	$p<0.0001$<br>$p<0.0001$(*lpin-1* RNAi glc)	Figure 6B
#2	N2/control RNAi glucose	13.1±0.3	14		100/120		
	N2/control RNAi glucose (myristic acid (14:0) + palmitic acid (16:0))	12±0.3	14	-8.3%	96/120	0.0375	
	N2/*lpin-1* RNAi glucose	11.2±0.1	12	-14.7%	110/116	$p<0.0001$	
	N2/*lpin-1* RNAi glucose (myristic acid (14:0) + palmitic acid (16:0))	10.8±0.2	12	-17.5%<br>-3.2%(*lpin-1* RNAi glc)	107/120	$p<0.0001$<br>0.477(*lpin-1* RNAi glc)	
#1	N2/control RNAi glucose	11.9±0.3	14		84/90		Figure 6C

	N2/control RNAi glucose (oleic acid (18:1n-9))	10.6±0.2	12	-11.1%	100/120	0.0011	Figure 6C
	N2/control RNAi glucose (linoleic acid, (18:2n-6))	14.4±0.3	16	20.3%	96/120	*p*<0.0001	
	N2/*lpin-1* RNAi glucose	10.2±0.1	10	-14.4%	116/120	*p*<0.0001	Figure 6D
	N2/*lpin-1* RNAi glucose (oleic acid (18:1n-9))	10.1±0.1	10	-15.2% -1.0%(*lpin-1* RNAi glc)	110/120	*p*<0.0001 0.5131(*lpin-1* RNAi glc)	Figure 6D
	N2/*lpin-1* RNAi glucose (linoleic acid, (18:2n-6))	12.1±0.3	14	1.5% 18.6%(*lpin-1* RNAi glc)	107/120	0.7866 *p*<0.0001(*lpin-1* RNAi glc)	
#2	N2/control RNAi glucose	14.3±0.4	17		99/120	*p*<0.0001	Figure 6E
	N2/control RNAi glucose (oleic acid (18:1n-9))	11.5±0.4	14	-19.9%	105/120	*p*<0.0001	
	N2/control RNAi glucose (linoleic acid (18:2n-6))	14.3±0.4	17	-0.2%	97/117	0.9611	Figure 6E
	N2/*lpin-1* RNAi glucose	10.2±0.1	11	-28.5%	102/120	*p*<0.0001	Figure 6F
	N2/*lpin-1* RNAi glucose (oleic acid (18:1n-9))	10.0±0.1	11	-30.2% -2.3%(*lpin-1* RNAi glc)	98/110	*p*<0.0001 0.1957(*lpin-1* RNAi glc)	
	N2/*lpin-1* RNAi glucose (linoleic acid, (18:2n-6))	11.6±0.3	14	-19.1% 13.2%(*lpin-1* RNAi glc)	106/120	*p*<0.0001 *p*<0.0001(*lpin-1* RNAi glc)	Figure 6F
#1	N2/control RNAi glucose	13.7±0.4	14		92/120		Figure 6G
	N2/*lpin-1* RNAi glucose	10.6±0.2	12	-22.3%	107/120	*p*<0.0001	Figure 6H
	N2/control RNAi glucose (arachidonic acid, (20:4n-6))	11.4±0.3	14	-16.7%	106/120	*p*<0.0001	Figure 6G
	N2/*lpin-1* RNAi glucose (arachidonic acid, (20:4n-6))	13.7±0.3	14	0.2% 29.0%(*lpin-1* RNAi glc)	93/120	0.7546 *p*<0.0001(*lpin-1* RNAi glc)	Figure 6H

#2	N2/control RNAi glucose	12.9±0.3	16		107/120		
	N2/*lpin-1* RNAi glucose	10.6±0.2	12	-17.8%	111/120	$p<0.0001$	
	N2/control RNAi glucose (arachidonic acid, (20:4n-6))	13.8±0.3	16	6.5%	106/120	0.0388	
	N2/*lpin-1* RNAi glucose (arachidonic acid, (20:4n-6))	12.7±0.2	16	-1.8%<br>19.5%(*lpin-1* RNAi glc)	116/120	0.4346<br>$p<0.0001$ (*lpin-1* RNAi glc)	

Lifespan data from the same experimental sets were indicated by bold lines and biological repeats were separated by bold dashed lines and stating trial numbers.

All *p* values were calculated by using log-rank test.

Percent change (%) and *p* values indicate comparison of each condition with ethanol (EtOH: solvent control)-treated control RNAi condition on glucose-rich diets within the same experimental dataset.

lpin-1 RNAi glc: percent change (%) and *p* values were calculated by comparing with EtOH-treated *lpin-1(RNAi)* worms on glucose diets within the same experimental dataset.

'Figures in text' indicates lifespan dataset that was used in designated Figures.

Table S7. Statistical analysis and additional repeats of lifespan assays with RNAi and transgene of fatty acid desaturases.

**Trial**	**Strain/treatment (fatty acid(s))**	**Mean lifespan ±SEM (days)**	**75th percentile**	**% change$^{\Delta}$**	**Number of animals that died/total**	***p* value vs. control**	**Figures in text**

#1	N2/control RNAi glucose	15.1±0.4	19		107/120		Figure S8A
	N2/*fat-6* RNAi (1/2) glucose	12.7±0.2	13	-15.9%	113/120	*p*<0.0001	Figure S8A
	N2/*lpin-1* RNAi (1/2) glucose	9.8±0.2	13	-35.1%	115/120	*p*<0.0001	Figure S8B
	N2/*fat-6/lpin-1* RNAi glucose	9.6±0.2	13	-36.4%<br>-2.0%(*lpin-1* RNAi glc)	108/120	*p*<0.0001<br>0.3944(*lpin-1* RNAi glc)	Figure S8B
	N2/*fat-2* RNAi (1/2) glucose	15.3±0.4	19	1.32%	102/120	0.7277	
	N2/*fat-4* RNAi (1/2) glucose	15.2±0.3	19	0.7%	105/120	0.9799	
#2	N2/control RNAi glucose	15.1±0.4	18				Figure S8C, D
	N2/*fat-6* RNAi (1/2) glucose	13.9±0.2	16	-7.9%	116/120	0.0001	
	N2/*lpin-1* RNAi (1/2) glucose	10±0.2	10	-33.8%	106/120	*p*<0.0001	
	N2/*fat-6/lpin-1* RNAi glucose	10.6±0.2	13	-29.8%<br>6.0%(*lpin-1* RNAi glc)	110/120	*p*<0.0001<br>0.0394(*lpin-1* RNAi glc)	
	N2/*fat-2* RNAi (1/2) glucose	16.1±0.3	18	6.6%	100/119	0.0654	Figure S8C
	N2/*fat-4* RNAi (1/2) glucose	16.9±0.3	20	11.9%	109/120	0.0002	Figure S8D
#1	N2/control RNAi glucose	14.9±0.3	18		105/120		Figure S8E
	N2/*lpin-1* RNAi glucose	10.3±0.2	12	-30.9%	105/120	*p*<0.0001	Figure S8E
	N2/*fat-1* RNAi glucose	14.8±0.3	18	-0.7%	106/120	0.8586	Figure S8E
	N2/*fat-1/lpin-1* RNAi glucose	10.8±0.2	12	-27.5%<br>4.9%(*lpin-1* RNAi glc)	112/120	*p*<0.0001<br>0.1524 (*lpin-1* RNAi glc)	Figure S8E
#2	N2/control RNAi glucose	13±0.4	16		117/120		
	N2/*lpin-1* RNAi glucose	9.7±0.2	12	-25.4%	114/120	*p*<0.0001	
	N2/*fat-1* RNAi glucose	13.1±0.3	16	0.8%	92/120	0.6709	

	N2/*fat-1/lpin-1* RNAi glucose	9.8±0.2	12	-24.6% 1.0%(lpin-1 RNAi glc)	115/120	*p*<0.0001 0.929(lpin-1 RNAi glc)	
#1	*odr-1p::rfp*/control RNAi glucose	13.8±0.4	17		100/120		Figure S8F
	odr-1p::rfp/*lpin-1* RNAi glucose	10±0.2	11	-27.5%	109/120	*p*<0.0001	Figure S8F
	fat-2p::fat-2::gfp/control RNAi glucose	14.8±0.3	18	7.2%	109/120	0.1554	Figure S8F
	fat-2p::fat-2::gfp /*lpin-1* RNAi glucose	9.2±0.2	10	-33.3% -8.0%(lpin-1 RNAi glc)	94/100	*p*<0.0001 *p*<0.0001(lpin-1 RNAi glc)	Figure S8F
#2	*odr-1p::rfp*/control RNAi glucose	13±0.3	15		114/120		
	odr-1p::rfp/*lpin-1* RNAi glucose	9.7±0.1	11	-25.4%	109/120	*p*<0.0001	
	fat-2p::fat-2::gfp/control RNAi glucose	13.7±0.3	16	5.4%	108/119	0.2637	
	fat-2p::fat-2::gfp /*lpin-1* RNAi glucose	9.2±0.2	10	-29.2% -5.15%(lpin-1 RNAi glc)	65/75	*p*<0.0001 0.0002(lpin-1 RNAi glc)	

Lifespan data from the same experimental sets were indicated by bold lines and biological repeats were separated by bold dashed lines and stating trial numbers.

All *p* values were calculated by using log-rank test.

Percent change (%) and *p* values were calculated by comparing each condition with control RNAi on control diets within the same experimental dataset.

lpin-1 RNAi glc: percent change (%) and *p* values were calculated by comparing with N2 or *odr-1p::rfp* treated with *lpin-1* RNAi on glucose diets within the same experimental dataset.

(1/2) indicates RNAi bacteria mixed with control RNAi bacteria (1:1 ratio)

‘Figures in text’ indicates lifespan dataset that was used in designated Figures.

Supporting References

Ashrafi, K., Chang, F. Y., Watts, J. L., Fraser, A. G., Kamath, R. S., Ahringer, J., & Ruvkun, G. (2003). Genome-wide RNAi analysis of *Caenorhabditis elegans* fat regulatory genes. *Nature, 421*(6920), 268-272. doi:10.1038/nature01279

Brock, T. J., Browse, J., & Watts, J. L. (2006). Genetic regulation of unsaturated fatty acid composition in *C. elegans*. *PLoS Genet, 2*(7), e108. doi:10.1371/journal.pgen.0020108

Consortium, T. U. (2019). UniProt: a worldwide hub of protein knowledge. *Nucleic Acids Res, 47*(D1), D506-d515. doi:10.1093/nar/gky1049

Dobin, A., Davis, C. A., Schlesinger, F., Drenkow, J., Zaleski, C., Jha, S., . . . Gingeras, T. R. (2013). STAR: ultrafast universal RNA-seq aligner. *Bioinformatics, 29*(1), 15-21. doi:10.1093/bioinformatics/bts635

Falcon, S., & Gentleman, R. (2007). Using GOstats to test gene lists for GO term association. *Bioinformatics, 23*(2), 257-258. doi:10.1093/bioinformatics/btl567

Gems, D., & Riddle, D. L. (2000). Defining wild-type life span in *Caenorhabditis elegans*. *J Gerontol A Biol Sci Med Sci, 55*(5), B215-219. doi:10.1093/gerona/55.5.b215

Han, S. K., Lee, D., Lee, H., Kim, D., Son, H. G., Yang, J. S., . . . Kim, S. (2016). OASIS 2: online application for survival analysis 2 with features for the analysis of maximal lifespan and healthspan in aging research. *Oncotarget, 7*(35), 56147-56152. doi:10.18632/oncotarget.11269

Hoogewijs, D., Houthoofd, K., Matthijssens, F., Vandesompele, J., & Vanfleteren, J. R. (2008).

Selection and validation of a set of reliable reference genes for quantitative sod gene expression analysis in *C. elegans*. *BMC Mol Biol, 9*, 9. doi:10.1186/1471-2199-9-9

Kaletsky, R., Yao, V., Williams, A., Runnels, A. M., Tadych, A., Zhou, S., . . . Murphy, C. T. (2018). Transcriptome analysis of adult *Caenorhabditis elegans* cells reveals tissue-specific gene and isoform expression. *PLoS Genet, 14*(8), e1007559. doi:10.1371/journal.pgen.1007559

Khuwijitjaru, P., Adachi, S., & Matsuno, R. (2002). Solubility of saturated fatty acids in water at elevated temperatures. *Biosci Biotechnol Biochem, 66*(8), 1723-1726. doi:10.1271/bbb.66.1723

Kosugi, S., Hasebe, M., Tomita, M., & Yanagawa, H. (2009). Systematic identification of cell cycle-dependent yeast nucleocytoplasmic shuttling proteins by prediction of composite motifs. *Proc Natl Acad Sci U S A, 106*(25), 10171-10176. doi:10.1073/pnas.0900604106

Larkin, M. A., Blackshields, G., Brown, N. P., Chenna, R., McGettigan, P. A., McWilliam, H., . . . Higgins, D. G. (2007). Clustal W and Clustal X version 2.0. *Bioinformatics, 23*(21), 2947-2948. doi:10.1093/bioinformatics/btm404

Lee, D., An, S. W. A., Jung, Y., Yamaoka, Y., Ryu, Y., Goh, G. Y. S., . . . Lee, S. V. (2019). MDT-15/MED15 permits longevity at low temperature via enhancing lipidostasis and proteostasis. *PLoS Biol, 17*(8), e3000415. doi:10.1371/journal.pbio.3000415

Lee, D., Jeong, D. E., Son, H. G., Yamaoka, Y., Kim, H., Seo, K., . . . Lee, S. J. (2015). SREBP and MDT-15 protect *C. elegans* from glucose-induced accelerated aging by preventing accumulation of saturated fat. *Genes Dev, 29*(23), 2490-2503. doi:10.1101/gad.266304.115

Lehner, B., Tischler, J., & Fraser, A. G. (2006). RNAi screens in *Caenorhabditis elegans* in a 96-

well liquid format and their application to the systematic identification of genetic interactions. *Nat Protoc, 1*(3), 1617-1620. doi:10.1038/nprot.2006.245

Li, B., & Dewey, C. N. (2011). RSEM: accurate transcript quantification from RNA-Seq data with or without a reference genome. *BMC Bioinformatics, 12,* 323. doi:10.1186/1471-2105-12-323

Liu, W., Xie, Y., Ma, J., Luo, X., Nie, P., Zuo, Z., . . . Ren, J. (2015). IBS: an illustrator for the presentation and visualization of biological sequences. *Bioinformatics, 31*(20), 3359-3361. doi:10.1093/bioinformatics/btv362

Love, M. I., Huber, W., & Anders, S. (2014). Moderated estimation of fold change and dispersion for RNA-seq data with DESeq2. *Genome Biol, 15*(12), 550. doi:10.1186/s13059-014-0550-8

Mariol, M. C., Walter, L., Bellemin, S., & Gieseler, K. (2013). A rapid protocol for integrating extrachromosomal arrays with high transmission rate into the *C. elegans* genome. *J Vis Exp*(82), e50773. doi:10.3791/50773

O'Rourke, E. J., Kuballa, P., Xavier, R., & Ruvkun, G. (2013). omega-6 Polyunsaturated fatty acids extend life span through the activation of autophagy. *Genes Dev, 27*(4), 429-440. doi:10.1101/gad.205294.112

Pathare, P. P., Lin, A., Bornfeldt, K. E., Taubert, S., & Van Gilst, M. R. (2012). Coordinate regulation of lipid metabolism by novel nuclear receptor partnerships. *PLoS Genet, 8*(4), e1002645. doi:10.1371/journal.pgen.1002645

Peterfy, M., Phan, J., Xu, P., & Reue, K. (2001). Lipodystrophy in the *fld* mouse results from mutation of a new gene encoding a nuclear protein, lipin. *Nat Genet, 27*(1), 121-124. doi:10.1038/83685

Pino, E. C., Webster, C. M., Carr, C. E., & Soukas, A. A. (2013). Biochemical and high throughput microscopic assessment of fat mass in *Caenorhabditis elegans*. *J Vis Exp*(73). doi:10.3791/50180

Reue, K., & Zhang, P. (2008). The lipin protein family: dual roles in lipid biosynthesis and gene expression. *FEBS Lett, 582*(1), 90-96. doi:10.1016/j.febslet.2007.11.014

Schneider, C. A., Rasband, W. S., & Eliceiri, K. W. (2012). NIH Image to ImageJ: 25 years of image analysis. *Nat Methods, 9*(7), 671-675.

Steinbaugh, M. J., Narasimhan, S. D., Robida-Stubbs, S., Moronetti Mazzeo, L. E., Dreyfuss, J. M., Hourihan, J. M., . . . Blackwell, T. K. (2015). Lipid-mediated regulation of SKN-1/Nrf in response to germ cell absence. *Elife, 4*. doi:10.7554/eLife.07836

Stiernagle, T. (2006). Maintenance of *C. elegans*. *WormBook*, 1-11. doi:10.1895/wormbook.1.101.1

Supek, F., Bosnjak, M., Skunca, N., & Smuc, T. (2011). REVIGO summarizes and visualizes long lists of gene ontology terms. *PLoS One, 6*(7), e21800. doi:10.1371/journal.pone.0021800

Yang, F., Vought, B. W., Satterlee, J. S., Walker, A. K., Jim Sun, Z. Y., Watts, J. L., . . . Naar, A. M. (2006). An ARC/Mediator subunit required for SREBP control of cholesterol and lipid homeostasis. *Nature, 442*(7103), 700-704. doi:10.1038/nature04942

Yang, J. S., Nam, H. J., Seo, M., Han, S. K., Choi, Y., Nam, H. G., . . . Kim, S. (2011). OASIS: online application for the survival analysis of lifespan assays performed in aging research. *PLoS One, 6*(8), e23525. doi:10.1371/journal.pone.0023525

Zhang, Y., Chen, D., Smith, M. A., Zhang, B., & Pan, X. (2012). Selection of reliable reference genes in *Caenorhabditis elegans* for analysis of nanotoxicity. *PLoS One, 7*(3), e31849.

doi:10.1371/journal.pone.0031849
